# Supplementary material for: Structural characterization of KKT4, an unconventional microtubule-binding kinetochore protein
Source: Structure. 2021 Sep 2;29(9):1014–1028.e8. doi: 10.1016/j.str.2021.04.004 (PMC8443799; doi:10.1016/j.str.2021.04.004)
Supplement: Document S1. Figures S1–S8 [file mmc1.pdf]

Structure, Volume *29*

Supplemental Information

Structural characterization of KKT4, an unconventional microtubule-binding kinetochore protein

Patryk Ludzia, Edward D. Lowe, Gabriele Marcianò, Shabaz Mohammed, Christina Redfield, and Bungo Akiyoshi

Figure S1

A

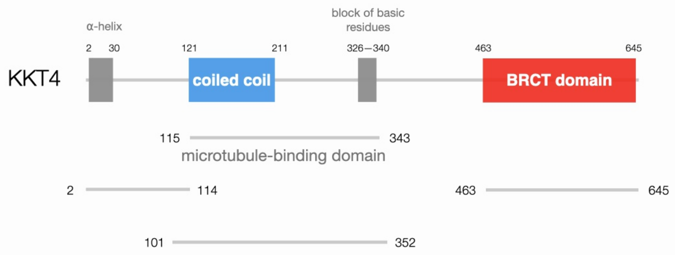

B

Protein	Concentration [μM]	Monomer molecular weight [kDa]	Observed molecular weight [kDa]	Estimated oligomerisation state
SNAP-6HIS-3FLAG-KKT4$^{2–645}$	20/17.5/10/6/4	92	334/275/240/195/170	Tetramer-dimer mixture
KKT4$^{463–645}$	100	20	19	Monomer
KKT4$^{101–352}$	35	28	55	Dimer
KKT4$^{2–114}$	1000/800/500/350/170	11	37.9/34.6/31.5/32.8/28.1	Tetramer–trimer or tetramer-dimer mixture

C

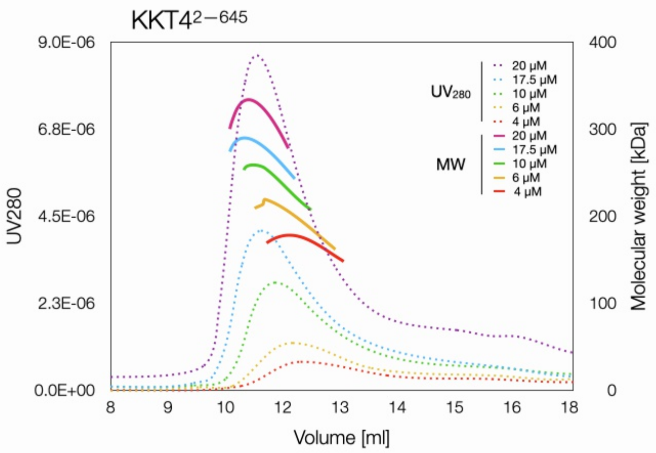

D

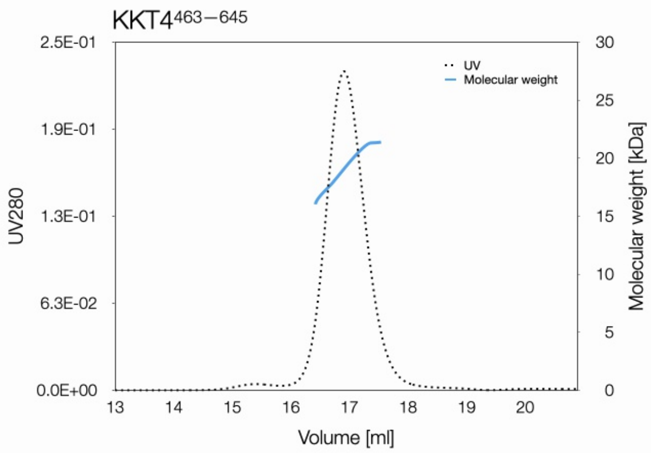

E

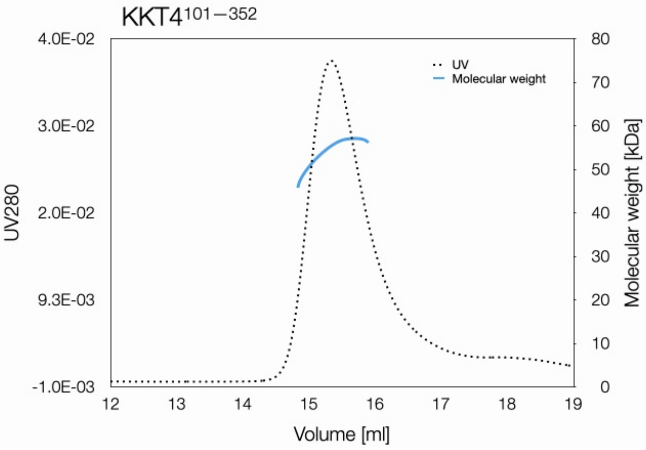

F

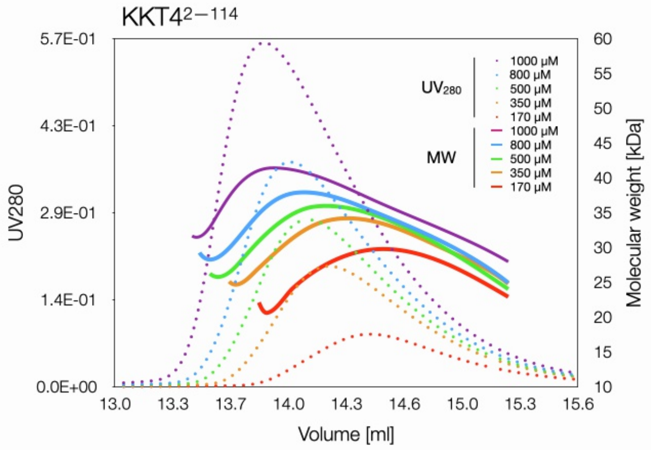

Figure S1. SEC-MALS analysis of KKT4 fragments, Related to Figure 1

A. Cartoon representation of KKT4 fragments used in SEC-MALS experiments

B. Summary table showing analysis of SEC-MALS results. Superose 6 HR10/300 was used for $KKT4^{2–645}$ and Superdex 200 HR10/300 was used for $KKT4^{463–645}$, $KKT4^{101–352}$ and $KKT4^{2–114}$. Reported sample concentration is the concentration before injecting the protein into the column. The actual concentration of the protein during the MALS experiment is ~10 fold lower due to the dilution factor during the SEC run.

C. SEC-MALS elution profile of SNAP-3FLAG-6HIS-$KKT4^{2–645}$ at different concentrations. The UV signal is plotted against the elution volume (dotted lines). Molecular weight is indicated as a thick line for each peak. The molecular weight at the highest concentration is estimated to be around 334 kDa, indicating a tetramer. The equilibrium shifts towards lower molecular weights, reaching 170 kDa (dimer) at the lowest concentration tested.

D. SEC-MALS elution profile of $KKT4^{463–645}$. The molecular weight is estimated to be around 19 kDa, indicating a monomer.

E. SEC-MALS elution profile of $KKT4^{101–352}$. The molecular weight is estimated to be around 55 kDa, indicating a dimer.

F. SEC-MALS elution profile of $KKT4^{2–114}$ samples at different concentrations. The lower the protein concentration, the larger the elution volume of the protein. This suggests that the oligomerisation state of $KKT4^{2–114}$ is concentration dependent, ranging from tetramer (high concentration) to trimer (low concentration).

Figure S2

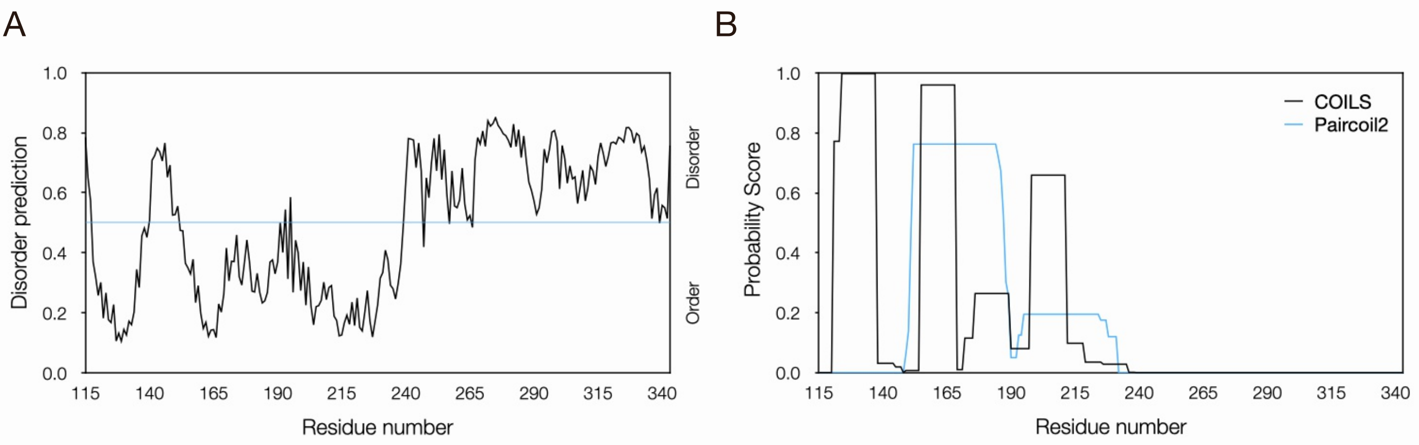

Figure S2. Structure predictions for *T. brucei* KKT4, Related to Figure 1

A. Disorder prediction for KKT4$^{115–343}$ using DisEMBL (Linding et al., 2003). The N-terminus of the microtubule-binding domain is predicted to be mainly ordered (118–239), while the C-terminus is predicted to be mostly disordered. The blue horizontal line at 0.5 indicates the boundary between predicted order (<0.5) and disorder (>0.5).

B. Coiled coil prediction for KKT4$^{115–343}$ using COILS (window of 14 residues) (Lupas et al., 1991) and Paircoil2 (window of 21/28 residues) (McDonnell et al., 2006). COILS predicts coiled coils from 121 to 211 with breaks between 138–154 and 169–189, while Paircoil2 does not predict coiled coil at the N-terminus of the domain between residues 121–137.

Figure S3

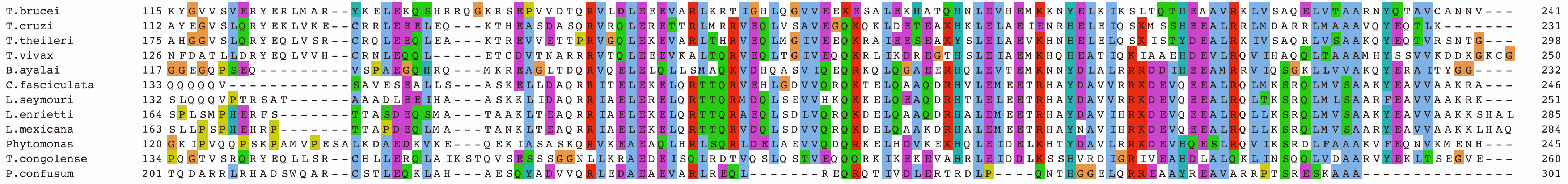

Figure S3. Multiple sequence alignment of the N-terminal part of KKT4 microtubule-binding domain, Related to Figure 2

KKT4 protein sequences from several kinetoplastid species were aligned using MAFFT (Katoh et al., 2019) and visualised with the CLUSTALX colouring scheme in Jalview (Waterhouse et al., 2009).

Figure S4

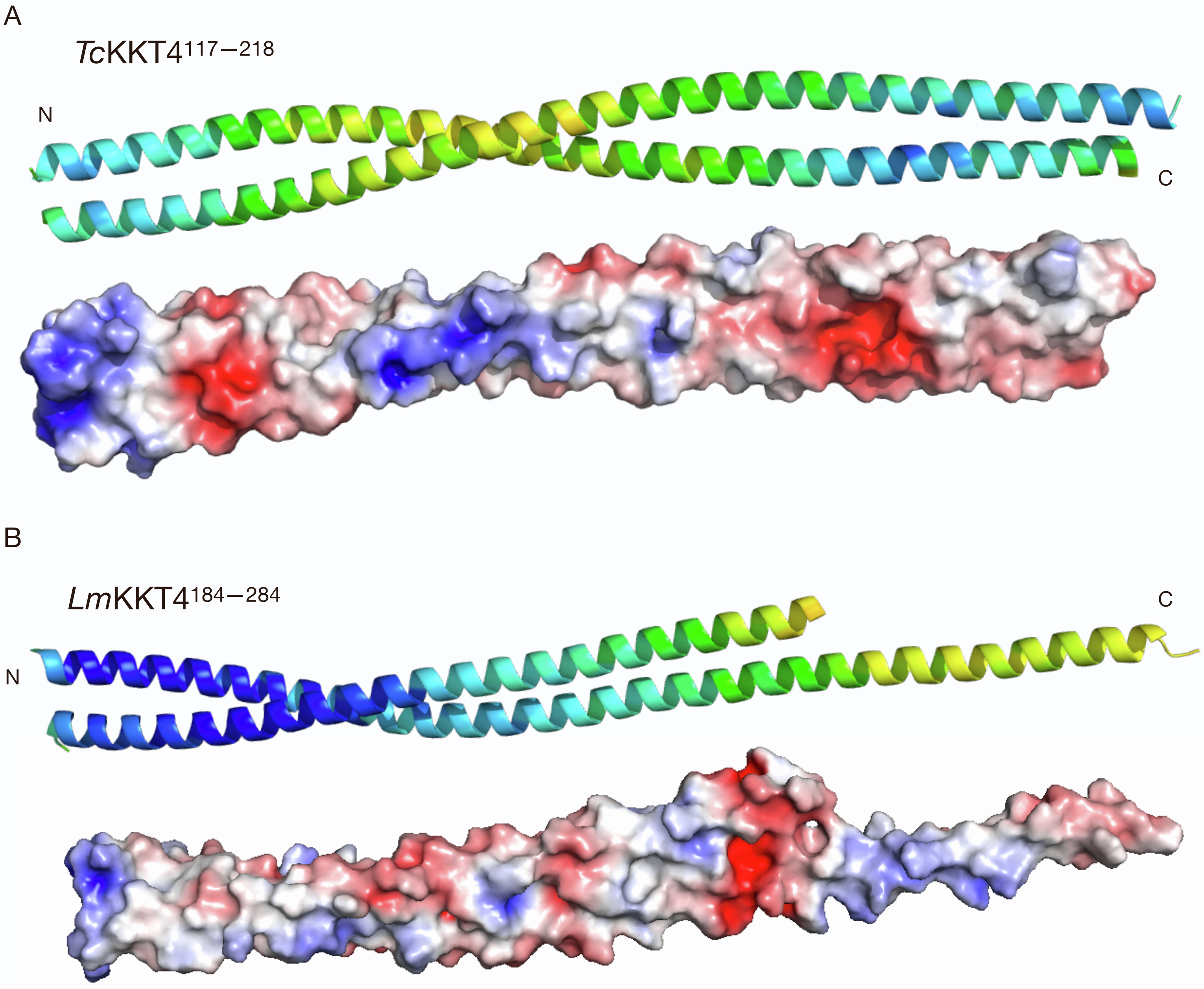

Figure S4. Ribbon models colour-coded by B-factors and the electrostatic surface potentials of *Tc*KKT4$^{117–218}$ and *Lm*KKT4$^{184–284}$, Related to Figure 2

B-factors for Cα atoms have been represented for *Tc*KKT4$^{117-218}$ (A) and *Lm*KKT4$^{184-284}$ (B) using a blue to red spectrum indicating low to high values, respectively. The figures were rendered using PyMol (DeLano, 2002). Below the ribbon models, a surface electrostatic potential is shown. Red to blue, -5 kbT to +5 kbT, as calculated by APBS electrostatic plugin in Pymol (Jurrus et al., 2018).

Figure S5

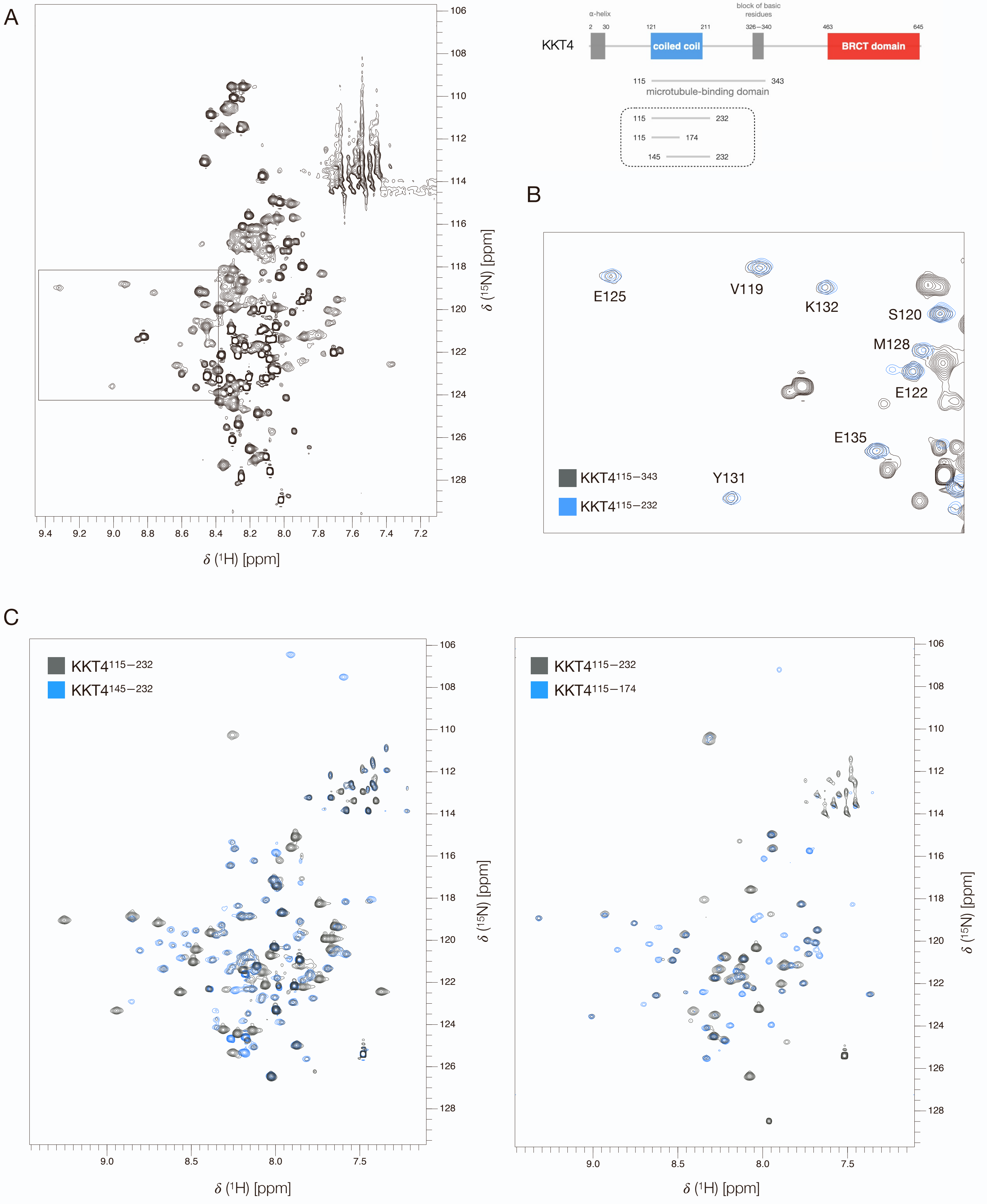
A
B
C
KKT4
α-helix
2 30
121 211
coiled coil
block of basic residues
326−340
463 645
BRCT domain
115 343
microtubule-binding domain
115 232
115 174
145 232
E125
V119
K132
S120
M128
E122
E135
Y131
KKT4115−343
KKT4115−232
KKT4145−232
KKT4115−174
δ (1H) [ppm]
δ (15N) [ppm]

Figure S5. ^{1}H-^{15}N BEST-TROSY spectra of KKT4 fragments. , Related to Figure 3

A. 750 MHz ^{1}H-^{15}N BEST-TROSY spectrum of KKT4$^{115–343}$. The spectrum is contoured so that both weak and strong peaks are visible. Peaks in the region of 111–114 ppm and upfield of ~7.6 ppm are artefacts in the BEST-TROSY arising from incomplete cancellation of signals from the side chain amides of asparagine and glutamine.

B. Overlay of a small region of the ^{1}H-^{15}N BEST-TROSY spectra of KKT4$^{115–343}$ (indicated with a box in (A) and KKT4$^{115–232}$. N-terminal peaks overlay well between the two constructs, suggesting that the structure of the KKT4 N-terminus is similar in both fragments.

C. Overlay of 750 MHz ^{1}H-^{15}N BEST-TROSY spectra of KKT4$^{115–232}$ and KKT4$^{115–174}$ (right panel) or KKT4$^{115–232}$ and KKT4$^{145–232}$ (left panel). Most of the peaks in the spectra of KKT4$^{115–174}$ and KKT4$^{145–232}$ overlay with peaks observed for KKT4$^{115–232}$, indicating that the dissection approach used was justified. Peaks that do not overlay arise from the different C-terminal sequence in KKT4$^{115–174}$ and N-terminal sequence in KKT4$^{145–232}$. Some peaks visible in the spectra of the shorter KKT4 fragments are not visible in the longer construct, KKT4$^{115–232}$.

Figure S6

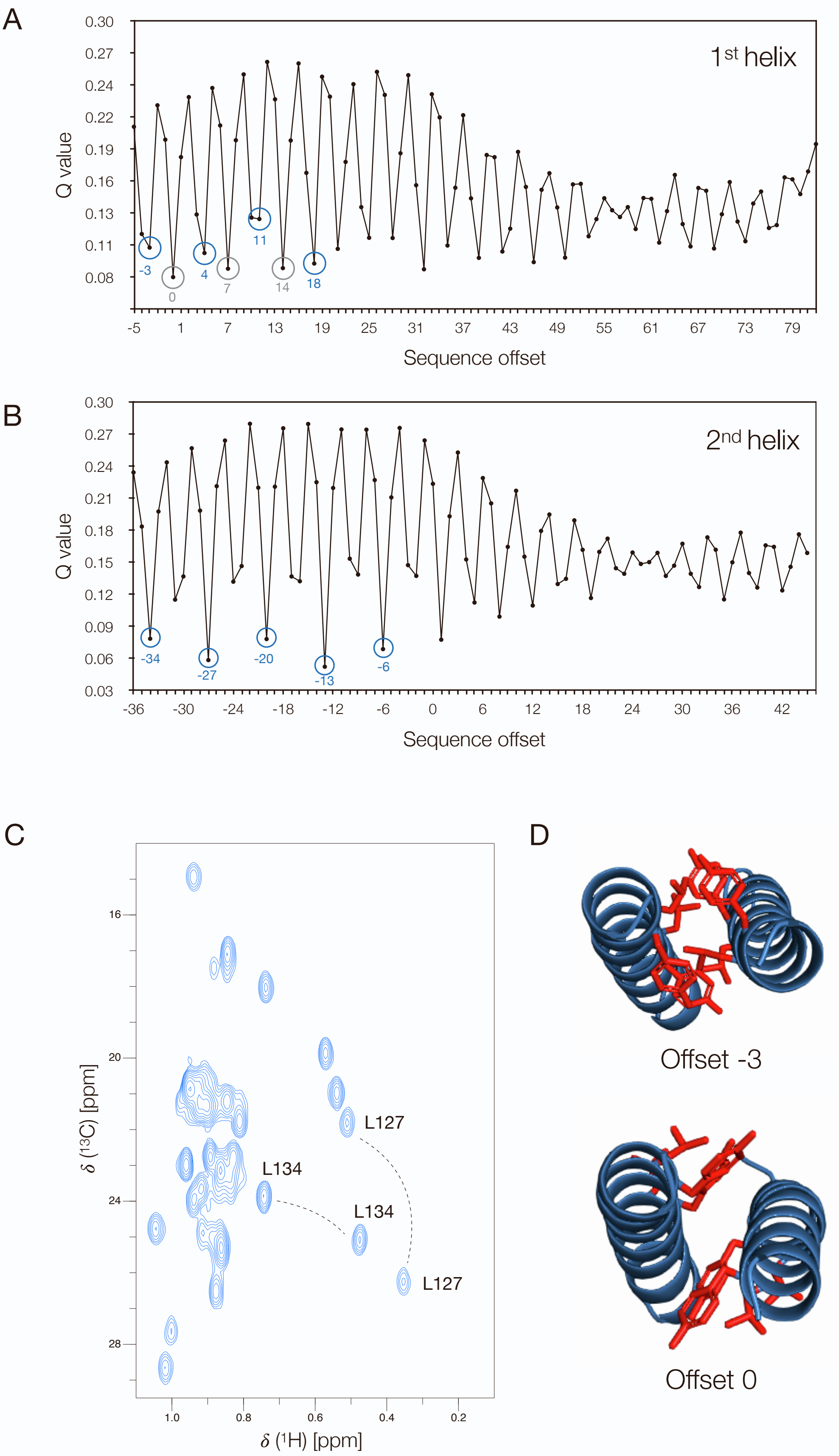
A
1st helix
Q value
Sequence offset
-3
0
4
7
11
14
18
B
2nd helix
Q value
Sequence offset
-34
-27
-20
-13
-6
C
δ (13C) [ppm]
δ (1H) [ppm]
L127
L134
L134
L127
D
Offset -3
Offset 0

Figure S6. Fitting the experimental RDC data using the *Tc*KKT4$^{117–218}$ crystal structure, Related to Figure 3

The experimental RDCs for residues 126–135 from the 1st helix (A) and 154–170 from the 2nd helix (B) from *T. brucei* KKT4$^{115–174}$ were fitted to the calculated RDCs based on the *T. cruzi* KKT4 X-ray structure. The relative alignment of the *T. brucei* and *T. cruzi* sequences was varied and the quality of the fit (Q value) of the experimental to predicted RDCs was measured. The Q value obtained is plotted as a function of the sequence offset (the number that is added to the *T. brucei* sequence before comparison with RDCs calculated for the *T. cruzi* sequence). For an ideal coiled-coil structure, the Q value should vary over a heptad repeat but the fits should be identical from one heptad repeat to the next. In contrast, for a more variable structure, where the interaction of the two helices varies along the length of the coiled coil, the quality of the fit may change along the sequence. For both helices, several good fits were found when the residues from *T. brucei* were aligned with the first half of the *T. cruzi* sequence, corresponding to the regular coiled-coil structure found between residues 121 and 176. Poorer agreement was obtained when the *T. brucei* helices were aligned with the C-terminal half of the *T. cruzi* structure, which shows less supercoiling. For the 2nd helix (residues 154–170, shown in (B) low Q values are found, at intervals of 7 residues (indicated by blue circles), when the residues from *T. brucei* were aligned with the residues in the N-terminal half of the *T. cruzi* sequence. The sequence alignment of these two proteins (Figure 2A) in the region of the 2nd helix suggests an offset of -6 residues, which is consistent with the fits of the RDC data, but offsets of -13, -20, -27 and -34 also produce good fits (indicated by blue circles). Predictions using the COILS server suggest that the hydrophobic residues identified as occupying the *a*/*d* positions in the *T. brucei* heptad align with the *a/d* residues in the *T. cruzi* structure for offsets of -6, -13, etc. For the 1st helix (residues 126–135, shown in (A)) low Q values are found for two sets of residues, at intervals of 7 residues. The lowest Q values are for sequence offsets of 0, 7, 14 (indicated by grey circles) but low Q values are also found for offsets of -3, 4, 11, 18 (indicated by blue circles); this type of degeneracy has been observed previously for the coiled-coil domain of cGK1α^{9-44} (Schnell et al., 2005). (C) The ^{1}H-^{13}C HSQC of KKT4$^{115-174}$ shows that both of the methyl groups of L127 and one of the methyl groups from L134 have ^{1}H peaks that are shifted upfield (to the right) of the peaks arising from most other L/V/I residues (around 0.7–1.05 ppm). These upfield shifts are due to the interaction of L127 and L134 with the aromatic rings of Y124 and Y131 and can be used to resolve the degeneracy between the two possible sequence offsets. Homology models for the 1st helix of *T. brucei* KKT4$^{115–174}$ have been built with Modeller using the *T. cruzi* X-ray structure and sequence alignments based on sequence offsets of -3 and 0 residues; these are shown in (D) with the side chains of Y124/L127/Y131/L134 shown as red sticks. The -3 sequence offset places Y124/Y131 and L127/L134 in positions *a* and *d* of the heptad, respectively, where they interact closely. The 0 sequence offset places these pairs of residues in positions *d* and *g*; L127/L134 in the *g* position are not closely packed at the inter-helix interface. ^{1}H chemical shifts for the L127 and L134 methyl groups were then predicted for the two alternative coiled-coil models. Only the homology model generated with a sequence offset of -3 residues predicts upfield shifts for the methyl groups of L127 and L134 due to their close proximity to Y124 and Y131 within the same helix and between helices in the packing interface. The sequence alignment of the *T. brucei* and *T. cruzi* proteins (Figure 2A) in the region of the 1st helix suggests an offset of -3 residues, which is consistent with the RDC data and chemical shift analysis of the homology models.

Figure S7

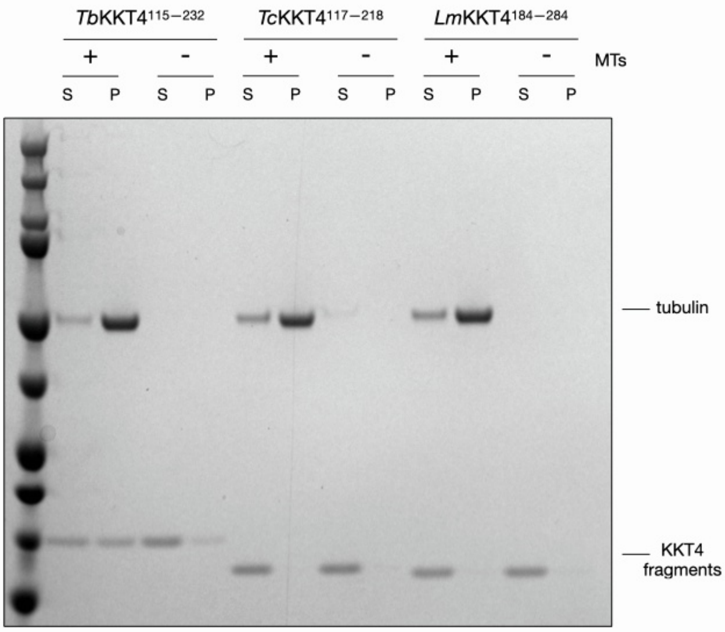

Figure S7. Microtubule-binding activity of KKT4 coiled coils, Related to Figure 5

Microtubule co-sedimentation assay of *Tb*KKT4$^{115-232}$, *Tc*KKT4$^{117-218}$ and *Lm*KKT4$^{184-284}$. Unlike *T. brucei* KKT4, coiled-coil regions from *T. cruzi* and *L. mexicana* did not co-sediment with taxol-stabilised microtubules at the concentration tested. S and P correspond to supernatant and pellet fractions, respectively.

Figure S8

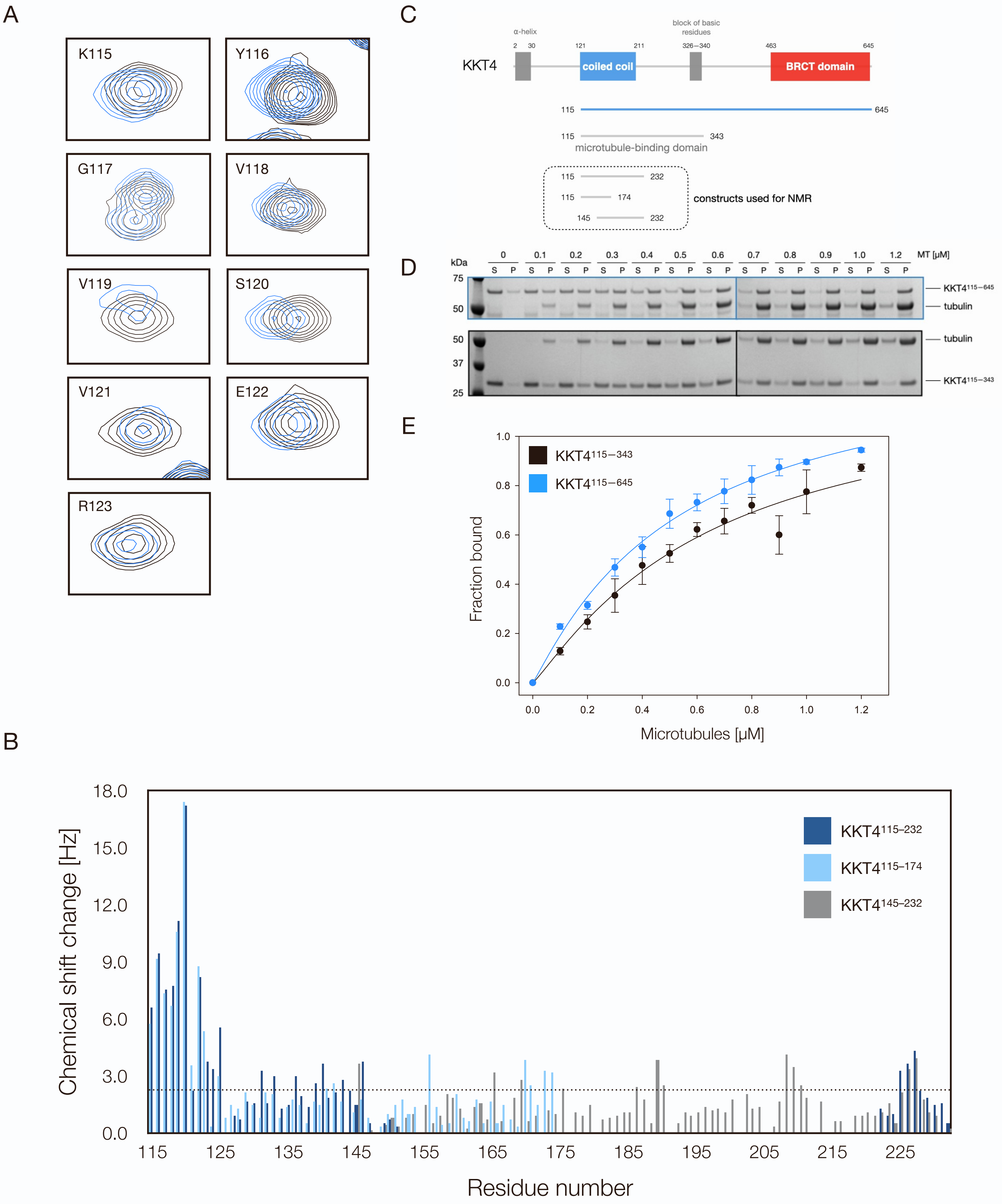

A
K115
Y116
G117
V118
V119
S120
V121
E122
R123
C
α-helix
block of basic residues
KKT4
2
30
121
211
326−340
463
645
coiled coil
BRCT domain
115
645
115
343
microtubule-binding domain
115
232
115
174
145
232
constructs used for NMR
D
kDa
0
0.1
0.2
0.3
0.4
0.5
0.6
0.7
0.8
0.9
1.0
1.2
MT [μM]
S
P
75
50
50
37
25
KKT4115−645
tubulin
tubulin
KKT4115−343
E
KKT4115−343
KKT4115−645
Fraction bound
Microtubules [μM]
0.0
0.2
0.4
0.6
0.8
1.0
1.2
B
Chemical shift change [Hz]
18.0
15.0
12.0
9.0
6.0
3.0
0.0
KKT4115–232
KKT4115–174
KKT4145–232
115
125
135
145
155
165
175
185
195
205
215
225
Residue number

Figure S8. N-terminus of microtubule-binding domain directly interacts with BRCT domain, Related to Figure 7

A. Magnification of peaks that showed chemical shift changes upon addition of the KKT4 BRCT domain to the sample. Peaks coming from ^{15}N-KKT4$^{115–232}$ and ^{15}N-KKT4$^{115–232}$/KKT4^{BRCT} spectra are coloured in black and blue respectively.

B. Chemical shift changes observed upon addition of KKT4^{BRCT} to KKT4$^{115–232}$ (dark blue), KKT4$^{115–174}$ (light blue), or KKT4$^{145–232}$ (grey) have been plotted against the KKT4 sequence. The dotted line represents the average value (2.2 Hz) of all chemical shift changes observed.

C. Cartoon representation showing KKT4 fragments used for NMR to study the interaction between the BRCT domain and the coiled coil-region. KKT4$^{115–645}$, analysed in panel D and E is shown as a blue line.

D. Microtubule co-sedimentation assay of KKT4$^{115−645}$ and KKT4$^{115−343}$, showing that the presence of the BRCT domain does not significantly affect the affinity to microtubules. S and P correspond to supernatant and pellet fractions, respectively.

E. Plot showing the fraction bound against the concentration of microtubules between KKT4$^{115–645}$ and KKT4$^{115–343}$. Error bars are standard deviations from three independent measurements.
