# Supplementary material for: Structural characterization of KKT4, an unconventional microtubule-binding kinetochore protein
Source: Structure. 2021 Sep 2;29(9):1014–1028.e8. doi: 10.1016/j.str.2021.04.004 (PMC8443799; doi:10.1016/j.str.2021.04.004)
Supplement: Document S2. Article plus supplemental information [file mmc8.pdf]

Structure

Article

Structural characterization of KKT4, an unconventional microtubule-binding kinetochore protein

Graphical abstract

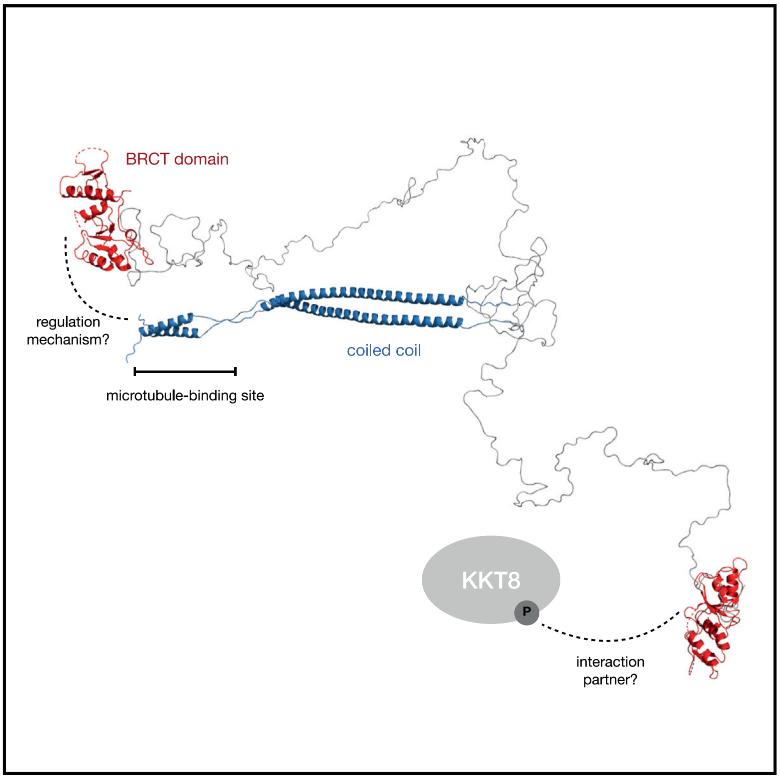

Highlights

- Structures of microtubule-binding and BRCT domains in KKT4 are reported
- The microtubule-binding domain consists of a coiled coil and a disordered tail
- KKT4 interacts with microtubules via a basic surface at the coiled-coil N terminus
- KKT4 has a phosphopeptide-binding BRCT domain

Authors

Patryk Ludzia, Edward D. Lowe, Gabriele Marcianò, Shabaz Mohammed, Christina Redfield, Bungo Akiyoshi

Correspondence

christina.redfield@bioch.ox.ac.uk (C.R.), bungo.akiyoshi@bioch.ox.ac.uk (B.A.)

In brief

KKT4 is a unique microtubule-binding kinetochore protein in trypanosomes. Structural analyses by Ludzia and colleagues show that its microtubule-binding domain consists of a coiled coil and a positively charged disordered tail. They also demonstrate that the C terminus of KKT4 is a phosphopeptide-binding BRCT domain.

Ludzia et al., 2021, Structure *29*, 1014–1028
September 2, 2021 © 2021 The Author(s). Published by Elsevier Ltd.
https://doi.org/10.1016/j.str.2021.04.004

CellPress

Structure

Article

Structural characterization of KKT4, an unconventional microtubule-binding kinetochore protein

Patryk Ludzia,[1] Edward D. Lowe,[1] Gabriele Marcianò,[1] Shabaz Mohammed,[1] Christina Redfield,[1,*] and Bungo Akiyoshi[1,2,*]
[1]Department of Biochemistry, University of Oxford, Oxford OX1 3QU, UK
[2]Lead contact
*Correspondence: christina.redfield@bioch.ox.ac.uk (C.R.), bungo.akiyoshi@bioch.ox.ac.uk (B.A.)
https://doi.org/10.1016/j.str.2021.04.004

SUMMARY

The kinetochore is the macromolecular machinery that drives chromosome segregation by interacting with spindle microtubules. Kinetoplastids (such as *Trypanosoma brucei*), a group of evolutionarily divergent eukaryotes, have a unique set of kinetochore proteins that lack any significant homology to canonical kinetochore components. To date, KKT4 is the only kinetoplastid kinetochore protein that is known to bind microtubules. Here we use X-ray crystallography, NMR spectroscopy, and crosslinking mass spectrometry to characterize the structure and dynamics of KKT4. We show that its microtubule-binding domain consists of a coiled-coil structure followed by a positively charged disordered tail. The structure of the C-terminal BRCT domain of KKT4 reveals that it is likely a phosphorylation-dependent protein-protein interaction domain. The BRCT domain interacts with the N-terminal region of the KKT4 microtubule-binding domain and with a phosphopeptide derived from KKT8. Taken together, these results provide structural insights into the unconventional kinetoplastid kinetochore protein KKT4.

INTRODUCTION

Every time a cell divides, it must duplicate and segregate its genetic material accurately into two daughter cells. A key structure involved in chromosome segregation in eukaryotes is the kinetochore, a macromolecular protein complex that assembles onto centromeric DNA and interacts with spindle microtubules during mitosis and meiosis (Mcintosh, 2016). Microtubules are dynamic polymers that change in length by addition or removal of tubulin subunits at the tips (Desai and Mitchison, 1997). Accurate chromosome segregation requires that kinetochores form robust attachments to the dynamic microtubule tips. In addition, kinetochores need to destabilize erroneous attachments to ensure that sister kinetochores bind microtubules emanating from opposite poles (Nicklas, 1997; Biggins, 2013; Cheeseman, 2014; Musacchio and Desai, 2017). Revealing the molecular basis of kinetochore-microtubule attachments and their regulation is key to understanding the mechanism of chromosome segregation.

Commonly studied eukaryotes have a number of microtubule-binding kinetochore proteins, including the Ndc80, Dam1, Ska, and SKAP-Astrin complexes (Cheeseman et al., 2001, 2006; Hanisch et al., 2006; Schmidt et al., 2012; Abad et al., 2014; Friese et al., 2016; Kern et al., 2017). Some of these components are widely conserved among eukaryotes (Meraldi et al., 2006, Van Hooff et al., 2017). However, none of these or other canonical structural kinetochore components has been identified in kinetoplastids, an evolutionarily divergent group of unicellular flagellated eukaryotes, such as parasitic trypanosomatids (e.g., *Trypanosoma brucei*, *Trypanosoma cruzi*, and *Leishmania* species) and free-living bodonids (e.g., *Bodo saltans*) (Berriman et al., 2005; Cavalier-Smith, 2010). Instead, a number of unique kinetochore proteins have been identified in *T. brucei*, namely 24 kinetoplastid kinetochore proteins (KKT1–20, KKT22–25) and 12 KKT-interacting proteins (KKIP1–12) (Akiyoshi and Gull, 2014, Nerusheva and Akiyoshi, 2016, D'archivio and Wickstead, 2017, Brusini et al., 2019, Nerusheva et al., 2019). These proteins do not appear orthologous to canonical kinetochore proteins, suggesting that kinetoplastids use a distinct set of proteins to build up unique kinetochores. We previously identified KKT4 as a microtubule-binding kinetochore protein in *T. brucei* (throughout this manuscript we refer to KKT4 from *Trypanosoma brucei* unless stated otherwise) (Llauro et al., 2018). KKT4 directly binds to microtubules and maintains load-bearing attachments to both growing and shortening microtubule tips *in vitro*. Microtubule-binding activities were also found in KKT4 from other kinetoplastids, suggesting that KKT4 plays an important role in the kinetoplastid family (Llauro et al., 2018). Using microtubule co-sedimentation assays, we defined KKT4$^{115-343}$ as the microtubule-binding domain in *T. brucei*. To date, there is no structural information available for KKT4. It therefore remains unknown how KKT4 forms attachments to microtubules

 © 2021 The Author(s). Published by Elsevier Ltd.
This is an open access article under the CC BY license (http://creativecommons.org/licenses/by/4.0/).

CellPress
OPEN ACCESS

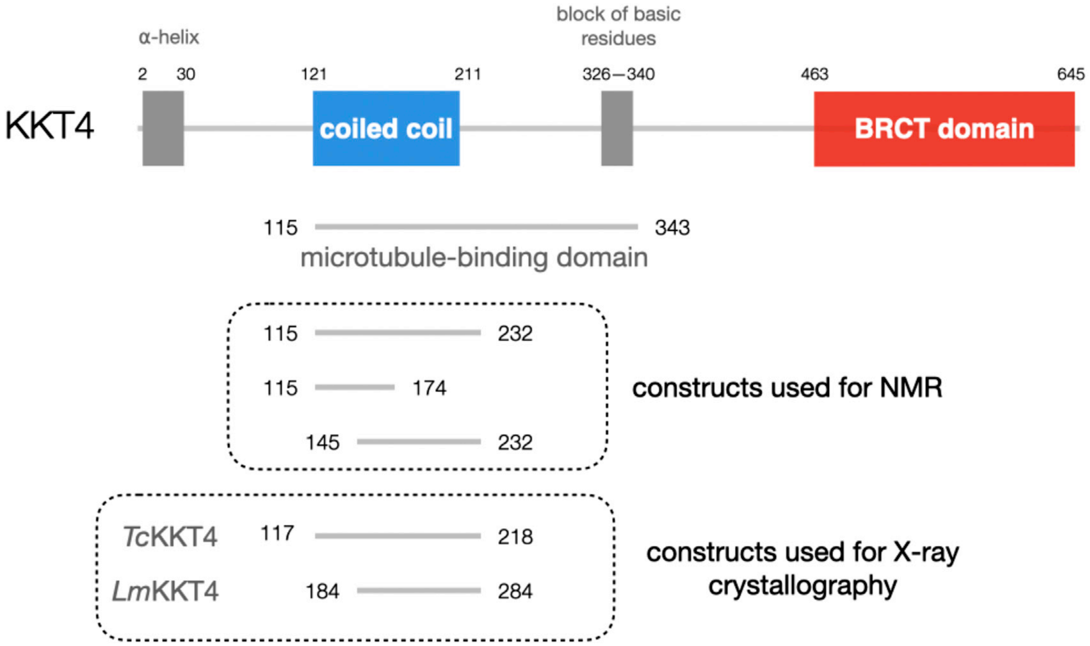

Figure 1. Domain organization for *T. brucei* KKT4
KKT4 has the following predicted regions conserved among kinetoplastid species: an N-terminal α helix, a coiled-coil region and a block of basic residues in the microtubule-binding domain, and a BRCT domain at the C terminus. The KKT4 fragments used for X-ray crystallography and NMR studies are shown in dashed boxes. See also Figures S1 and S2 and Table S6.

and whether its microtubule-binding activity is regulated. Interestingly, KKT4 has a putative BRCA1 C-terminal (BRCT) domain, which is not present in any known kinetochore protein in other eukaryotes (Figure 1) (Akiyoshi and Gull, 2014). The function of this putative BRCT domain is also unknown.

Here, we have used X-ray crystallography and NMR spectroscopy to obtain structural information for KKT4. Its microtubule-binding domain consists of a coiled-coil structure followed by a positively charged disordered tail. A crystal structure of the C-terminal BRCT domain reveals a putative phosphopeptide-binding pocket, which binds a phosphopeptide derived from KKT8. Overall, these analyses show that the KKT4 structure is distinct from any known microtubule-binding kinetochore protein.

RESULTS

KKT4 forms oligomers

Our previous single-molecule experiments suggested that KKT4 was mostly monomeric but had a tendency to oligomerize even at a low nanomolar concentration (Llauro et al., 2018). To determine the oligomerization state of KKT4, we used size-exclusion chromatography coupled with multi-angle light scattering (SEC-MALS) (Wen et al., 1996) (Figure S1). Analysis of the full-length protein revealed that KKT4 is mostly tetrameric at higher concentrations and dimeric at lower concentrations (Figures S1B and S1C). To identify the region(s) responsible for oligomerization, smaller fragments of KKT4 were analyzed. The following predicted structural regions of KKT4 are well conserved among kinetoplastids: N-terminal α helix, coiled coil and block of basic residues within the microtubule-binding domain, and the C-terminal BRCT domain (Figures 1 and S2) (Llauro et al., 2018). We found that KKT4$^{463–645}$, a putative BRCT domain, behaved as a monomer (Figures S1B and S1D), while KKT4$^{101–352}$, containing the microtubule-binding domain, behaved as a dimer (Figures S1B and S1E). KKT4$^{2–114}$, like the full-length protein, showed characteristics of a tetramer at higher concentrations but of a trimer at lower concentrations (Figures S1B and S1F). Thus, the N-terminal region is responsible for the formation of the KKT4 tetramer. It is likely that KKT4$^{2–114}$ is in a dimer-tetramer equilibrium (rather than trimer-tetramer) because the microtubule-binding domain is a dimer and the full-length protein is likely to be a dimer of dimers. These results suggest that KKT4 has multiple regions that promote oligomerization.

Crystal structures of *T. cruzi* KKT4$^{117–218}$ and *L. mexicana* KKT4$^{184–284}$

To gain structural insights into the microtubule-binding domain of KKT4, we tried to crystalize KKT4$^{115–343}$. Despite extensive attempts, no suitable crystals were obtained. It is likely that the predicted disordered region in the C-terminal part of KKT4$^{115–343}$ prevented the formation of diffraction-quality crystals (Figure S2A). We next designed additional constructs that lack the predicted disordered tail. Although we still failed to obtain diffraction-quality crystals from *T. brucei*, a crystal structure of KKT4$^{117–218}$ from *T. cruzi* was solved at 1.9 Å resolution (Table 1). *Tc*KKT4$^{117–218}$ is homologous to KKT4$^{120–224}$ in *T. brucei* (Figures 2A and S3). The structure consists of two ~150 Å long parallel α helices organized in a left-handed coiled-coil dimer (Figure 2B); the helical structure starts at L118 and ends at D215. The coiled coil consists of eight regular heptad repeats starting at Y121 and ending at K176. Analysis

Table 1. Data collection, refinement statistics[a]

Data collection	*T. cruzi* KKT4$^{117–218}$	*L. mexicana* KKT4$^{184–284}$	*T. brucei* KKT4$^{463–645}$
Beamline	Diamond Light Source I03	Diamond Light Source I24	Diamond Light Source I24
Wavelength (Å)	0.9760	0.91587	0.96861
Space group (Z)	$P\ 1\ 2_1\ 1$	$P\ 1\ 2_1\ 1$	$P\ 2_1\ 2_1\ 2_1$
Unit cell			
a, b, c (Å)	33.62, 25.31, 136.88	31.31, 37.71, 122.39	46.37, 61.63, 67.78
α, β, γ(°)	90, 96.81, 90	90, 92.17, 90	90, 90, 90
Resolution range (Å)	67.96–1.90 (1.97–1.90)	61.15–1.90 (1.97–1.90)	45.60–1.57 (1.63–1.57)
Unique reflections	16,812 (368)	22,522 (2,211)	27,032 (2,137)
Completeness (%)	90.4 (20.1)	98.3 (97.4)	96.8 (74.7)
Multiplicity	6.1 (4.0)	6.5 (5.3)	10.0 (3.8)
I/σI	6.1 (0.1)	7.1 (0.6)	14.9 (3.1)
R_{merge}	0.095 (7.495)	0.117 (2.537)	0.089 (0.549)
CC½	0.998 (0.274)	0.997 (0.352)	0.997 (0.496)
Wilson B factor (Å^2)	24.4	17.6	15.7
Refinement			
No. of reflections	16,788 (368)	22,502 (2,211)	26,945 (2,109)
R_{work}	0.241 (0.347)	0.199 (0.253)	0.173 (0.219)
R_{free}	0.258 (0.478)	0.232 (0.264)	0.192 (0.303)
No. of atoms	1,871	1,816	1,494
Protein	1,670	1,488	1,270
Solvent	201	328	224
RMSD			
Bonds (Å)	0.006	0.014	0.011
Angles (°)	0.97	1.45	1.46
Ramachandran plot (%)			
Favored	100.00	100.00	98.68
Allowed	0.00	0.00	1.32
Outliers	0.00	0.00	0.00
Average B factor (Å^2)	36.0	30.0	22.0

[a]Statistics for the highest-resolution shell are shown in parentheses. RMSD, root-mean-square deviation.

with TWISTER (Strelkov and Burkhard, 2002) shows that the six central heptads are characterized by an inter-helical distance of 4.84 ± 0.06 Å and a coiled-coil pitch (the periodicity of the coiled coil) of 136 ± 26 Å; this pitch is close to the theoretical value of 135 Å for a coiled coil. If the first and eighth heptad are included, the pitch increases to 163 ± 60 Å, indicating less supercoiling in these terminal heptads. After K176, the helices move apart (inter-helical distance increases to 5.66 ± 0.15 Å) and the coiled-coil pitch increases significantly, indicating a loss of supercoiling.

To test structural conservation in other kinetoplastid species, we attempted to solve the crystal structure of the KKT4 coiled-coil fragment from *L. mexicana*. We were unable to obtain diffraction-quality crystals of fragments that had the N-terminal residues of the microtubule-binding domain (115–140 in *T. brucei*). Instead, we crystalized and solved a 1.9 Å structure of *Lm*KKT4$^{184–284}$ (Table 1), which corresponds to residues 141–244 in *T. brucei* (Figure S3). We note that the expressed protein contained an additional 23 residues from the expression vector at its C terminus due to a cloning error (see the STAR Methods for details). Like *Tc*KKT4$^{117–218}$, *Lm*KKT4$^{184–284}$ has helices arranged in a parallel coiled-coil fold (Figure 2C). *Lm*KKT4$^{184–284}$ has six regular heptad repeats from the N terminus to Q224, and after that point the helices move apart and lose their supercoiling as observed for *Tc*KKT4$^{117–218}$. The two chains in *Lm*KKT4$^{184–284}$ differ in length, with helices spanning T184 to the C terminus in one chain and T184 to R257 in the other. The region beyond I245, where the helices have moved away from each other, had high B factors in both chains, suggesting enhanced flexibility (Figure S4B). The electron density of the shorter helix disappears at Q258, most likely due to the disordered nature of the protein backbone in this region. Interestingly, the end of the longer helix, which contains extra residues from the expression vector, makes seemingly stabilizing contacts with other molecules in the crystal lattice, which potentially explains why our attempts to obtain diffraction-quality crystals for the proper construct ending at Q284 failed. A DALI search for structural homologs of *Tc*KKT4$^{117–218}$ and *Lm*KKT4$^{184–284}$ identified similarity to a number of coiled-coil proteins (Tables S1 and S2). Superposition of the coiled coils from *T. cruzi* and *L. mexicana* revealed a good structural match with a root-mean-square

CellPress
OPEN ACCESS

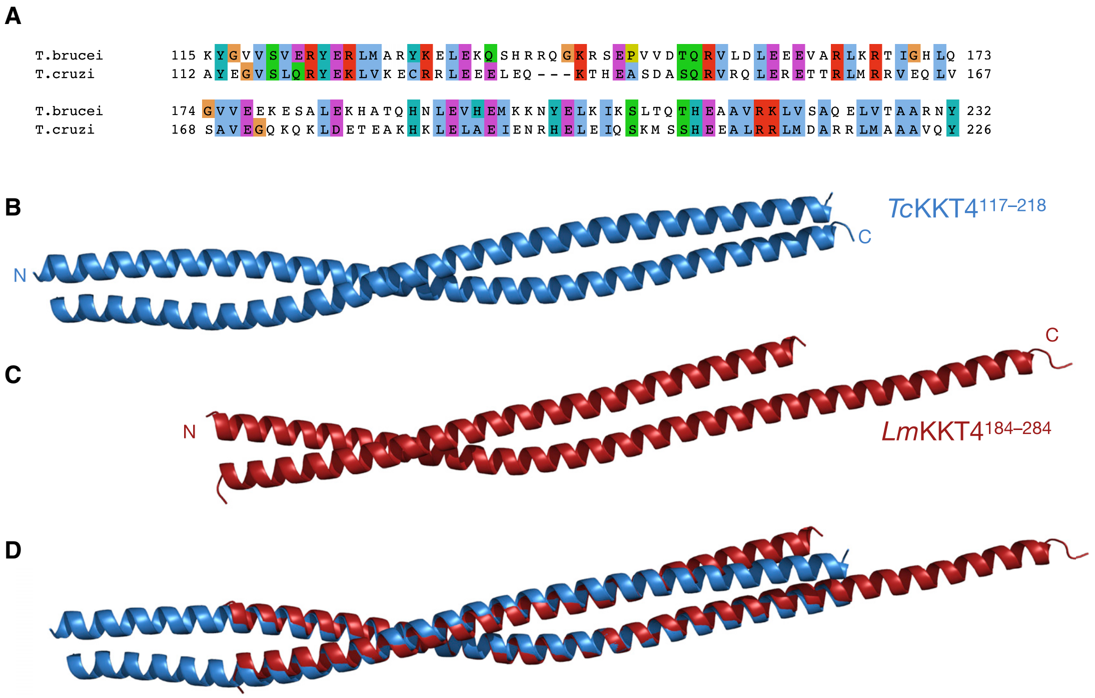

Figure 2. Crystal structures of *T. cruzi* KKT4$^{117–218}$ and *L. mexicana* KKT4$^{184–284}$ reveal parallel coiled coils

(A) Sequence alignment of the KKT4 microtubule-binding domains of *T. brucei* and *T. cruzi* (Sylvio X10) with the CLUSTALX coloring scheme in Jalview (Waterhouse et al., 2009). Ribbon models of the *T. cruzi* (Sylvio X10) KKT4$^{117–218}$ (B) and *L. mexicana* KKT4$^{184–284}$ (C) backbones. The N and C termini are indicated by N and C, respectively. Superposition of the two structures (D), using the *align* function in PyMOL (Delano, 2002), gives an RMSD of 1.03 Å. See also Figures S3 and S4 and Tables S1 and S2.

deviation (RMSD) of 1.03 Å (for 123 Cα) (Figure 2D), confirming the conservation of the coiled-coil structure in these species.

The C-terminal part of the KKT4 microtubule-binding domain is disordered

Due to the failure to crystalize *T. brucei* KKT4, we employed NMR spectroscopy to probe the structure and dynamics of its microtubule-binding domain. The 2D ^{1}H-^{15}N correlation spectrum of ^{15}N-KKT4$^{115–343}$ showed a large variation in peak intensities (Figure S5A); this suggests a mixture of structured and disordered regions. The strongest peaks in the spectrum of KKT4$^{115–343}$ belong to residues 115–118 in the N terminus and 231–343 in the C-terminal half of the fragment (Ludzia et al., 2020). Weaker peaks belong to residues 119–150, while no peaks were observed for residues 151–230 (Ludzia et al., 2020).

Backbone chemical shifts are sensitive indicators of secondary structure (Spera and Bax, 1991; Wishart et al., 1991; Beger and Bolton, 1997; Cornilescu et al., 1999). Analysis of ^{1}Hα, ^{1}HN, ^{13}Cα, ^{13}Cβ, ^{13}CO, and ^{15}N chemical shifts using TALOS-N (Shen and Bax, 2013) predicted no stable secondary structure for residues 231–343 (data not shown). The secondary structure propensity (SSP) score, which is more suitable for identifying structural propensities in disordered proteins (Marsh et al., 2006), also found no SSP greater than 0.25 (Figure 3A), from residues 231–343 in KKT4$^{115–343}$.

To probe the dynamics of the C terminus of KKT4$^{115–343}$, the {^{1}H}-^{15}N heteronuclear NOE, which is sensitive to backbone motions on a timescale (picosecond) faster than the overall tumbling of the molecule (nanosecond), was measured (Kay et al., 1989). A {^{1}H}-^{15}N NOE ratio of less than 0.6, indicating a flexible backbone, was found for all residues from 231 to 343 (Figure 3B). Taken together, the NMR data confirmed disorder in the C-terminal part of KKT4$^{115–343}$.

The N-terminal part of the KKT4 microtubule-binding domain is structured

The N-terminal region of KKT4$^{115–343}$ is predicted to adopt a coiled-coil structure (Figure S2B). To gain insights into the structure and dynamics of this region of KKT4, NMR data were collected for a shorter fragment (KKT4$^{115–232}$) lacking the flexible C-terminal region. However, the spectrum of KKT4$^{115–232}$ also lacked peaks from residues 151–221; this is likely due to the elongated structure of a coiled coil, which would tumble in a non-uniform way and result in broad ^{1}HN-^{15}N signals (Mackay et al., 1996; Schnell et al., 2005). To overcome this problem, two shorter overlapping constructs were used for further NMR analysis (Figure 1): KKT4$^{115–174}$, the minimal microtubule-binding domain that retains reduced microtubule-binding activities (Llauro et al., 2018), and KKT4$^{145–232}$ that was identified as a stable fragment in trypsin digests of KKT4$^{115–343}$ (Ludzia et al., 2020). The 2D spectra of these constructs contained peaks for all residues (Ludzia et al., 2020), and comparison with the spectrum of KKT4$^{115–232}$ (Figure S5C) indicates that these shorter constructs retain the structural and dynamical properties observed in the longer fragment. Analysis of the chemical shifts of KKT4$^{115–174}$ and KKT4$^{145–232}$ using TALOS-N revealed

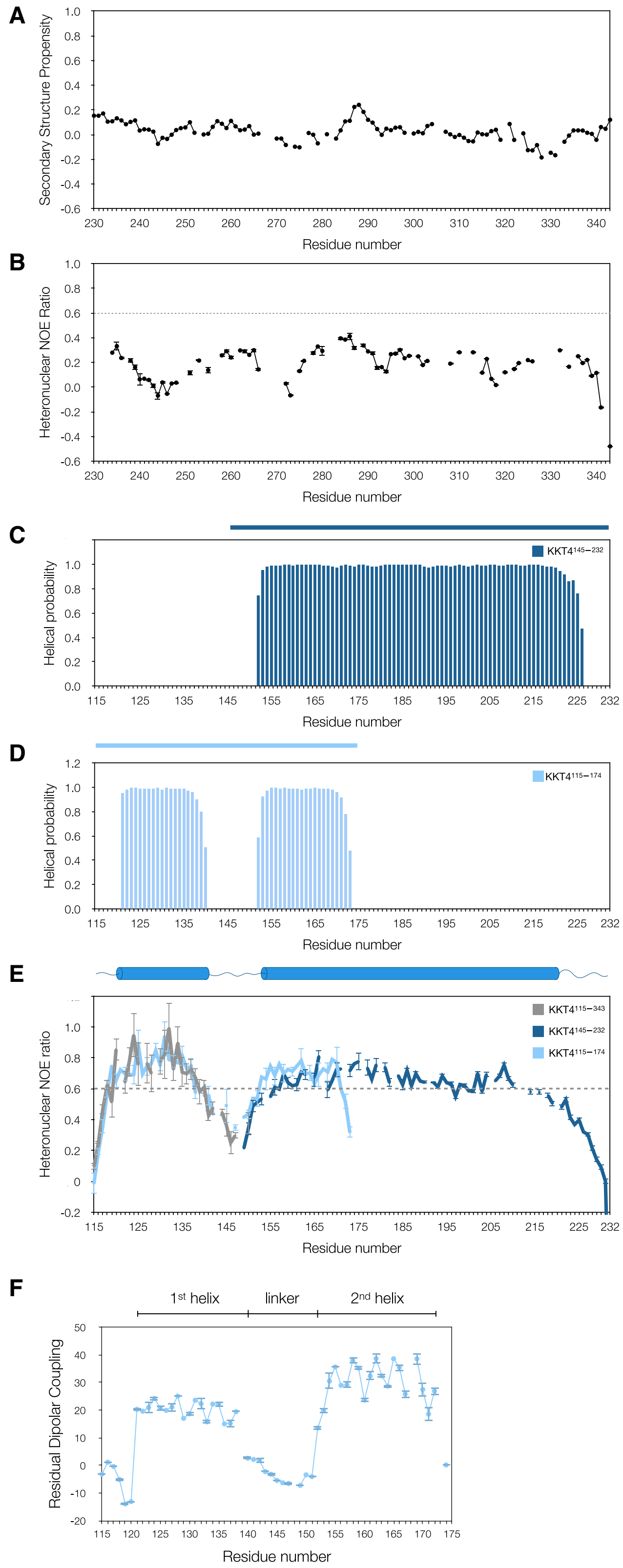

Figure 3. NMR analysis of the KKT4 microtubule-binding domain

(A) Secondary structure propensity (SSP) scores (Marsh et al., 2006) for residues 230–343 of KKT4$^{115–343}$ show no helical (+) or sheet (−) propensity greater than 0.25.

(B) The {^{1}H}-^{15}N heteronuclear NOE ratios measured for KKT4$^{115–343}$ are less than 0.6 for residues 231–343, showing that this region is disordered. {^{1}H}-^{15}N NOE errors here and in (E) were estimated from 500 Monte Carlo simulations using baseline noise as a measure of peak height error.

(C) TALOS-N secondary structure prediction for KKT4$^{145–232}$ shows a continuous helix from 152 to 225.

(D) TALOS-N secondary structure prediction for KKT4$^{115–174}$ shows two regions of helical structure, 121–139 and 152–172, separated by an unstructured linker. The blue bars above the graphs in (C and D) indicate the length of the KKT4 fragment used for data collection and TALOS-N analysis.

(E) {^{1}H}-^{15}N Heteronuclear NOE ratios are plotted for ^{15}N-KKT4$^{115–174}$ (light blue), ^{15}N-KKT4$^{145–232}$ (dark blue), and ^{15}N-KKT4$^{115–343}$ (gray). Values for the C terminus of KKT4$^{115–343}$ are shown in (B). The heteronuclear NOE values in the region of the first helix for KKT4$^{115–343}$ and KKT4$^{115–174}$ agree well, suggesting that removal of the disordered C terminus did not affect the properties of the KKT4$^{115–174}$ N terminus. The lower ratios at the beginnings and ends of the helices may indicate helix fraying. The C terminus of KKT4$^{115–174}$ has significantly lower heteronuclear NOE ratios compared with the same residues in KKT4$^{145–232}$ because G174 is the artificially designed C terminus of KKT4$^{115–174}$. The value for the C-terminal residue Y232 (−1.16) in KKT4$^{145–232}$ is not shown for clarity of the figure. The top panel shows a summary cartoon of the overall secondary structure of KKT4$^{115–232}$ predicted by TALOS-N.

(F) Experimental ^{1}H-^{15}N residual dipolar couplings (RDCs) for KKT4$^{115–174}$, measured in 5% C12E6/n-hexanol, are plotted as a function of sequence. The RDCs for both helices show periodic variation that is consistent with a coiled-coil structure. The RDCs close to 0 for the linker region indicate that this region is dynamic. Error bars are standard deviations from three RDC measurements. See also Figures S5 and S6.

significant amounts of secondary structure (Figures 3C and 3D). For KKT4$^{145–232}$, a continuous helix was observed from 152 to 225 (Figure 3C). For KKT4$^{115–174}$, two helices, encompassing residues 121–139 and 152–172, separated by an unstructured linker were observed (Figure 3D).

The {^{1}H}-^{15}N NOE ratios for KKT4$^{115–174}$ and KKT4$^{145–232}$ indicate dynamics that are consistent with the predicted secondary structure (Figure 3E). {^{1}H}-^{15}N NOE ratios of greater than ~0.6, characteristic of a structured backbone, were found for most residues that were predicted to be helical. In KKT4$^{115–174}$, residues 140–151, which were not predicted to be helical, had lower {^{1}H}-^{15}N NOE ratios, confirming the presence of a disordered dynamic linker between the two helices (Figure 3E).

The helical regions identified in *T. brucei* KKT4 by NMR match those observed in the crystal structures of *Tc*KKT4$^{117–218}$ and *Lm*KKT4$^{184–284}$. However, in both crystal structures, we did not find a flexible linker within the coiled coils that we identified in *T. brucei* KKT4. Instead, we observed elevated B factors (Figure S4A) for the region where we might expect to find an unstructured linker in the *Tc*KKT4 structure based on the sequence alignment (Figure S3). We speculate that the lack of a flexible linker in *T. cruzi* and *L. mexicana* crystal structures may be either due to the stabilizing contacts within the crystal lattice or structural differences of KKT4 between *T. brucei* and the other two kinetoplastids.

In summary, the microtubule-binding domain of *T. brucei* KKT4 is composed of two helices, encompassing residues 121–139 and 152–225, separated by a 12-residue flexible linker,

CellPress
OPEN ACCESS

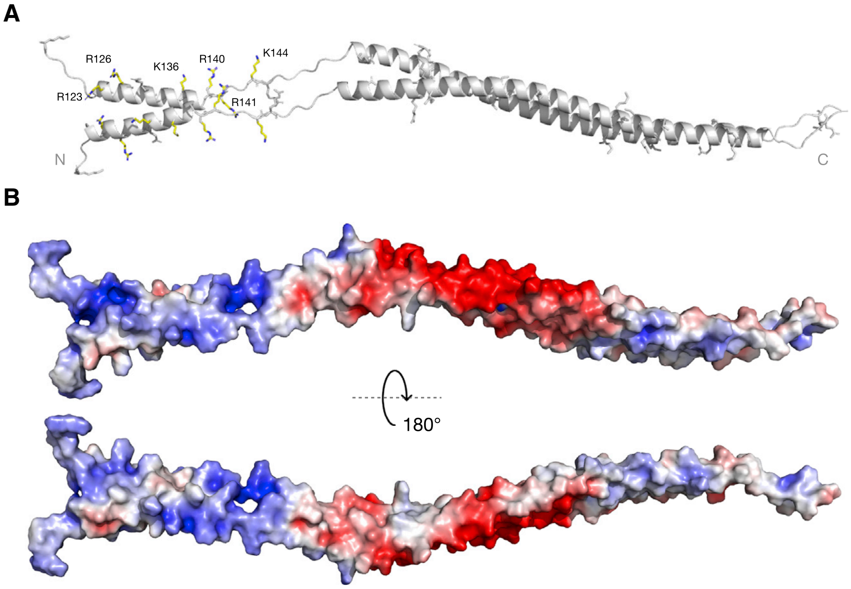

Figure 4. Homology model of *T. brucei* KKT4$^{115–232}$

(A) Ribbon representation of the *T. brucei* KKT4$^{115–232}$ structural model generated using MODELLER (Webb and Sali, 2016). The model, based on NMR, X-ray crystallography, and sequence alignment between *T. brucei* and *T. cruzi* KKT4 shows two coiled-coil segments separated by an unstructured linker and flanked by small disordered regions at the N and C termini of the molecule. Lysine and arginine side chains are presented as sticks. Residues for which charge-reversal mutations affected the microtubule-binding activity of KKT4 (see Figure 6) are shown in yellow and labeled.

(B) Homology model of KKT4$^{115–232}$ colored by surface electrostatic potential, showing positively charged patches in the N-terminal helix. Red to blue, −5 kbT to +5 kbT, as calculated using the APBS electrostatic plugin in PyMOL (Jurrus et al., 2018).

followed by a ~120-residue positively charged disordered region (predicted isoelectric point for residues 226–343 is 10.1).

Modeling of the *T. brucei* KKT4 coiled coil

We next aimed to determine if the helices identified by NMR in *T. brucei* KKT4 are organized into dimeric coiled coils as observed in the crystal structures of the *T. cruzi* and *L. mexicana* homologs. Residual dipolar couplings (RDCs), measured for partially aligned protein samples, are sensitive to N-H bond vector orientation and can be used to distinguish between undistorted and supercoiled helices. In the latter, the helical turns at the packing interface (residues *a*, *d*, *e*, *g* in the heptad repeat) are slightly compressed, while those facing outside (*b*, *c*, *f*) are stretched; this leads to a periodic variation in the RDCs within the heptad repeat (Schnell et al., 2005). Both helices in KKT4$^{115–174}$ show large positive RDCs with the periodic variation that is consistent with a coiled-coil structure (Figure 3F). In contrast, the N and C termini and the flexible linker are dynamic, which leads to averaging of their RDCs to values close to 0.

Using the *T. cruzi* X-ray structure as a model, we tested different ways of fitting the helices identified by NMR into a coiled-coil structure by optimizing the fit between RDCs predicted from the X-ray structure and the experimental RDCs (Figures S6A and S6B). For both helices in *T. brucei*, good fits were found when they were placed within the first half of the *T. cruzi* sequence, corresponding to the regular coiled-coil structure while poorer agreement was obtained using the less supercoiled C-terminal half of the *T. cruzi* structure. For the first helix, the fit of the experimental RDCs suggests an offset of -3 residues between the *Tb* and *Tc* sequences (Figure S6A), while for the second helix the fit of the RDCs suggests an offset of -6 residues (Figure S6B). These offsets are in agreement with the alignment of the *Tb* and *Tc* sequences (Figure 2A) and place hydrophobic residues in *T. brucei* in positions *a*/*d* of the heptad repeat in the *T. cruzi* structure.

A homology model for the two coiled-coil regions of *T. brucei* KKT4$^{115–232}$ was built using the *T. cruzi* coiled-coil X-ray structure, and the sequence alignments confirmed using the RDC data (Webb and Sali, 2016). Random extended structures, which represent possible conformations that might be sampled, for the flexible N and C termini and inter-helix linker were generated. These coordinates were merged with the coiled-coil homology models to generate an overall model for *T. brucei* KKT4$^{115–232}$ (Figure 4A).

Positively charged disordered tail enhances microtubule-binding activity

Our structural analysis suggested that the KKT4 microtubule-binding domain in *T. brucei* consists of two regions: the N-terminal coiled coil and C-terminal unstructured basic tail (Figures 2 and 3). To evaluate their contribution to KKT4's affinity for microtubules, the coiled-coil region (KKT4$^{115–232}$), the basic unstructured tail (KKT4$^{233–343}$), and the full microtubule-binding domain (KKT4$^{115–343}$) were purified and tested in microtubule co-sedimentation assays (Figures 5A and 5B). The basic disordered region (KKT4$^{233–343}$) did not co-sediment with Taxol-stabilized microtubules, consistent with our previous finding using KKT4$^{168–343}$ (Llauro et al., 2018). In contrast, the coiled coil alone (KKT4$^{115–232}$) co-sedimented, albeit with a lower affinity compared with KKT4$^{115–343}$. These results suggest that, although the disordered tail cannot interact strongly with microtubules on its own, it enhances the microtubule-binding activity of the coiled-coil domain. To compare the binding affinities between KKT4$^{115−343}$ and KKT4$^{115−232}$, we varied the microtubule concentration and calculated their dissociation constants by quantifying the percentage of co-sedimented KKT4 fragments (Figures 5C and 5D). This analysis confirms that KKT4$^{115−343}$ has higher affinity for microtubules ($K_D \sim 0.65$ μM) than the coiled-coil region ($K_D \sim 1.1$ μM).

We previously showed that the KKT4 microtubule-binding domain from different kinetoplastid species (*T. cruzi*, *L. mexicana*, *T. congolence*, and *Phytomonas*) co-sediments with Taxol-stabilized microtubules (Llauro et al., 2018). Unlike *T. brucei* KKT4, the coiled-coil fragments from *T. cruzi* and *L. mexicana*

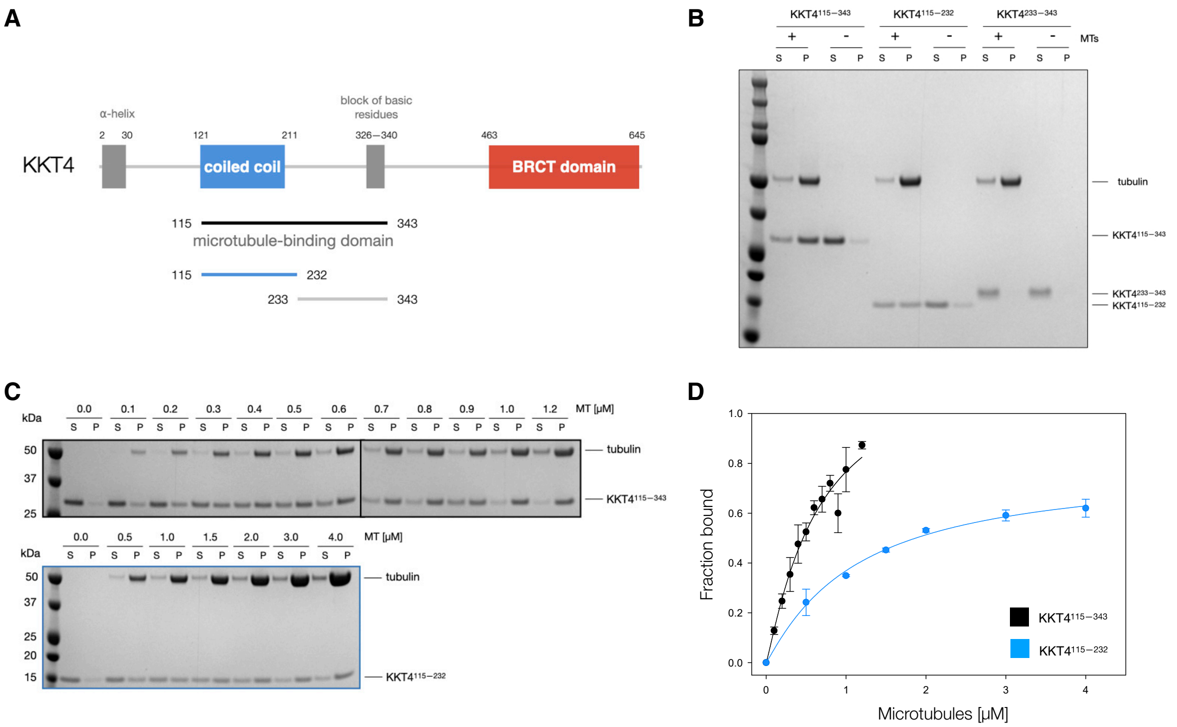

Figure 5. *T. brucei* KKT4 coiled coil is sufficient to interact with microtubules *in vitro*

(A) Cartoon representation of KKT4 fragments used for quantitative analysis of microtubule-binding activity.

(B) Microtubule co-sedimentation assay of KKT4$^{115–343}$, KKT4$^{115–232}$, and KKT4$^{233–343}$, showing that the KKT4 coiled coil (KKT4$^{115–232}$) is sufficient to interact with microtubules (although with weaker affinity compared with KKT4$^{115–343}$). The unstructured basic region (KKT4$^{233–343}$) does not co-sediment with microtubules, but its presence in KKT4$^{115–343}$ enhances the microtubule-binding activity of KKT4. S and P correspond to supernatant and pellet fractions, respectively.

(C) Microtubule co-sedimentation assay of KKT4$^{115–343}$ and KKT4$^{115–232}$ with increasing concentrations of microtubules.

(D) Plot showing the fraction bound against the concentration of microtubules. Error bars are standard deviations from three independent measurements. See also Figure S7.

that were used in our structural analysis (*Tc*KKT4$^{117–218}$ and *Lm*KKT4$^{184–284}$) failed to bind microtubules in our co-sedimentation assay (Figure S7). This suggests that the KKT4 coiled-coil region from different kinetoplastid species may interact with microtubules with different affinities. Indeed, weaker microtubule binding was observed in our previous work of KKT4 microtubule-binding domains from these species (Llauro et al., 2018), which could be explained by minor differences in their structure or surface charges. Further work needs to be done to examine the differences in the microtubule-binding activity of KKT4 in other kinetoplastid species.

Mapping the microtubule-binding interface of KKT4

Microtubule interaction is often mediated by the electrostatic effects of surface charges (Ciferri et al., 2008). In fact, we previously showed that a charge-reversal mutant that replaced three basic residues with acidic residues (R123E, K132E, and R154E) severely reduced the microtubule-binding activity of *T. brucei* KKT4$^{115–343}$ (Llauro et al., 2018). To understand the charge distribution of the coiled coil in *T. brucei* KKT4, we used our homology model to calculate the electrostatic surface potential (Jurrus et al., 2018). This revealed positively charged regions in the N terminus of the coiled-coil structure (Figure 4B), with basic residues exposed on the protein surface (e.g., R123, R126, R130, K132, K136, and R140) (Figure 4A). To test the importance of positively charged residues for microtubule binding, we systematically generated single mutants of KKT4$^{115–343}$, where lysine and arginine within the N-terminal region (115–232) were replaced with glutamic or aspartic acid. Microtubule binding of these mutants was compared with that of wild-type KKT4$^{115–343}$. We found that mutating any of residues R123, R126, K136, R140, R141, and K144 significantly reduced the microtubule-binding affinity (Figures 6A and 6B). These residues are located within KKT4$^{115–174}$, which was previously identified as the minimal microtubule-binding domain (Llauro et al., 2018). In contrast, mutating basic residues located in the second, longer helix in the coiled coil of the protein (K154, R164, K166, R167, K179, K198, K199, K204, K206, R217, K218, and R230) had only mild effects on the microtubule-binding activity (Figures 6A and 6B). To test if mutations affected the stability of the structure, 1D ^{1}H NMR spectra were collected for mutants that had reduced affinity to microtubules (R126, K132, K136, R140, R141, and K144); these confirmed that the mutations did not disrupt the coiled-coil structure (data not shown). Together with our previous analysis (Llauro et al., 2018), these results confirmed the importance of positively charged residues for microtubule-binding activities and revealed that the primary microtubule-binding interface of *T. brucei* KKT4 is likely the basic surface located in the N-terminal coiled coil.

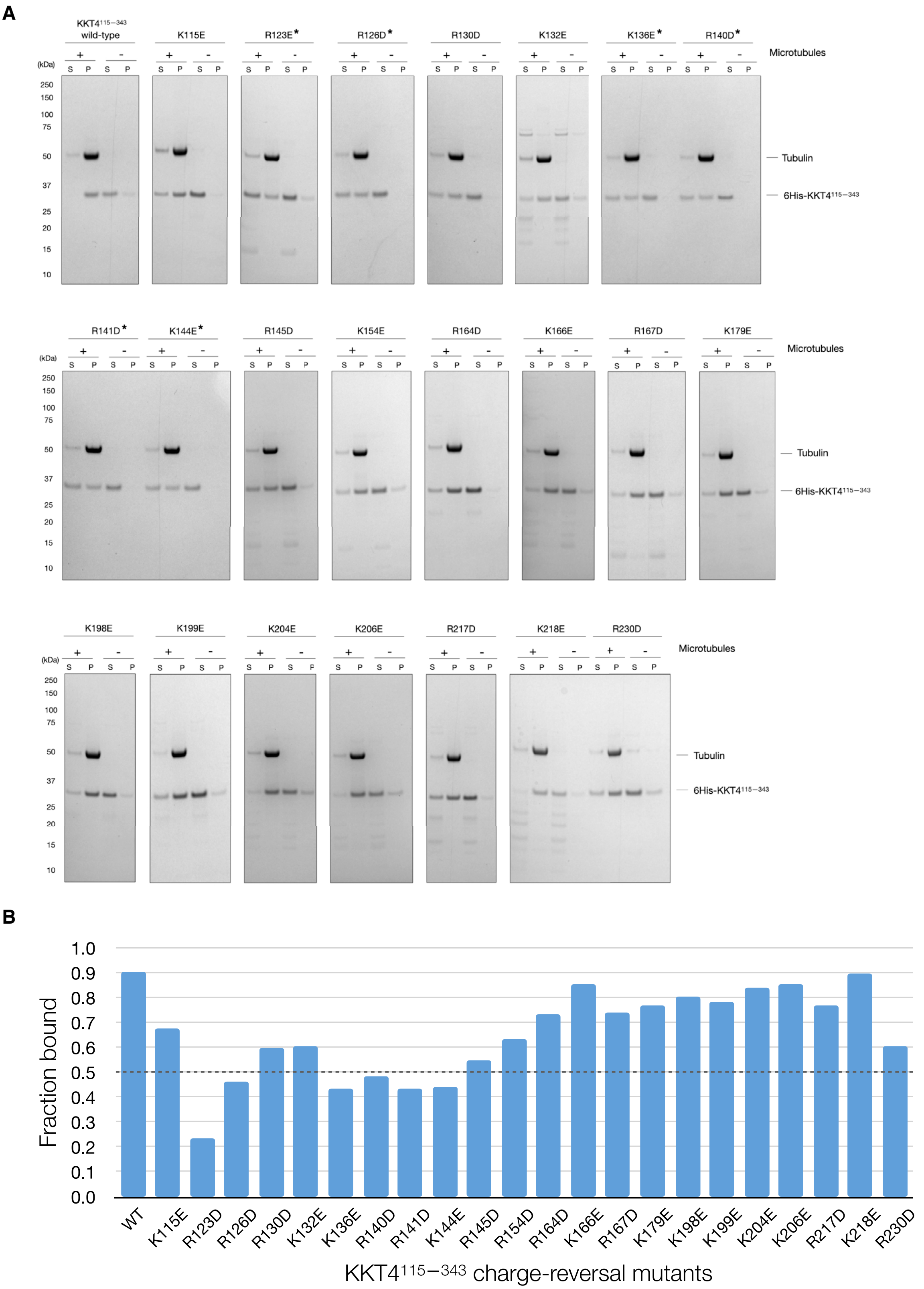

(legend on next page)

KKT4 has DNA-binding activity

In many species, kinetochore assembly is regulated during the cell cycle. In humans, the constitutive centromere-associated network (CCAN) is composed of 16 chromatin-proximal kinetochore proteins that act as a platform for kinetochore assembly by interacting with CENP-A nucleosomes (Cheeseman and Desai, 2008). Most of the CCAN components constitutively localize at kinetochores and some of them have DNA-binding activities. In contrast, microtubule-binding kinetochore components localize only during mitosis. In *T. brucei*, kinetochore assembly is regulated during the cell cycle. However, the microtubule-binding protein KKT4 localizes at kinetochores in a constitutive manner (Akiyoshi and Gull, 2014). Interestingly, the C terminus of KKT4 is predicted to be a tandem BRCT domain (Akiyoshi and Gull, 2014). BRCT domains are found in a number of prokaryotic and eukaryotic proteins with various functions including DNA or RNA binding (Zhang et al., 1998; Yu et al., 2003; Leung and Glover, 2011). We previously observed significant DNA contamination during the purification of full-length KKT4 from insect cells (Llauro et al., 2018), suggesting that KKT4 might have DNA-binding activity, possibly via its BRCT domain. To test this, we employed fluorescence anisotropy assays (Rossi and Taylor, 2011) using a fluorescently labeled 50-bp double-stranded DNA. We found that full-length KKT4 ($KKT4^{2–645}$) strongly bound DNA (K_D = 11 nM) (Figure 7A), while the KKT4 BRCT domain ($KKT4^{463–645}$) failed to saturate the DNA signal under the same conditions (Figure 7A). These results suggest that the BRCT domain of KKT4 does not bind DNA tightly and that the high-affinity DNA-binding site is located elsewhere.

Crystal structure of the *T. brucei* KKT4 BRCT domain

To understand the function of the KKT4 BRCT domain, we solved its structure using X-ray crystallography. $KKT4^{463–645}$ yielded crystals that diffracted to a resolution of 1.6 Å (Table 1). BRCT domains typically comprise ~90–100 residues with the βαββαβα secondary structure topology (Leung and Glover, 2011). The structure of $KKT4^{463–645}$ revealed tandem BRCT domains (Figure 7B). The N-terminal full domain (BRCT1) consists of a central four-stranded β sheet flanked by two α helices on one side of the sheet and one α helix on the opposite side. The smaller domain (BRCT2) consists of two α helices and three β strands, missing a β strand and an α helix in the C terminus (Figure 7B). No electron density was observed for residues 463–473, 519–523, and 617–625, suggesting that these regions are flexible. A search for structural homologs using DALI (Holm, 2019) revealed similarity to several BRCT-containing proteins with breast cancer-associated protein 1 (BRCA1) as one of the top hits (Table S3). The tandem BRCT domains in BRCA1 have a highly conserved phosphopeptide-binding pocket (Clapperton et al., 2004; Shiozaki et al., 2004; Williams et al., 2004). Superposition of KKT4 BRCT1 with the N-terminal BRCT domain of *H. sapiens* BRCA1 showed a good structural match with an RMSD of 1.11 Å (for 65 Cα) (Figure 7C), suggesting that the KKT4 BRCT domain may bind phosphopeptides. In fact, we observed additional electron density in our structure, likely arising from a sulfate ion that may mimic a bound phosphate group. The sulfate ion is coordinated by three residues in the pocket, T494, S495, and K543 (Figure 7D), which correspond to the key residues known to interact with phosphopeptides in other BRCT domains (e.g., S1655, G1656, and K1702 in human BRCA1) (Williams et al., 2004). These results suggest that the BRCT domain of KKT4 likely functions as a phosphorylation-dependent protein-protein interaction domain rather than a DNA-binding domain.

The KKT4 BRCT domain is a phosphopeptide-binding domain

To identify potential binding partners for $KKT4^{BRCT}$ domain (KKT4463, sequences of kinetochore proteins were searched for the BRCT consensus motif (pS/pT)-x-x-(F/Y/I/L) (Manke et al., 2003; Yu et al., 2003). Among those proteins that co-purified with KKT4 (Akiyoshi and Gull, 2014), we identified possible motifs in KKT7 (SVTF, residues 65–68), KKT8 (SVRY, residues 381–384), and KKT12 (SILL, residues 192–195), which are highly conserved among kinetoplastids (Figure 7E and data not shown). Fluorescently labeled phosphopeptides derived from these proteins were tested for $KKT4^{BRCT}$ binding using a fluorescence anisotropy assay. The peptide derived from KKT8 bound $KKT4^{BRCT}$ with a K_D of ~30 μM (Figure 7F), while the other two peptides failed to bind $KKT4^{BRCT}$ with a similar affinity (data not shown). Importantly, we found that the non-phosphorylated KKT8 peptide bound $KKT4^{BRCT}$ with significantly weaker affinity (Figure 7F). Furthermore, replacement of K543, located in the putative phosphopeptide-binding site in $KKT4^{BRCT}$ with alanine, decreased the binding affinity by roughly an order of magnitude (Figure 7F). These results show that $KKT4^{BRCT}$ is a phosphopeptide-binding domain and identify KKT8 as a potential interaction partner.

The KKT4 BRCT domain interacts with the microtubule-binding region

To obtain further structural information on KKT4, we used crosslinking mass spectrometry (XL-MS), which can identify interaction surfaces between partner proteins or within the same molecule (Mattson et al., 1993; Leitner et al., 2016). Crosslinking on full-length KKT4 was carried out using two different crosslinkers: (1) BS^3, a homo-bifunctional crosslinker that reacts with primary amines and covalently links pairs of lysines that are within 26–30 Å on the protein surface and (2) zero-length EDC and Sulfo-NHS that activates carboxyl groups for reaction with primary amines. XL-MS of KKT4 resulted in numerous crosslinks across the molecule, and similar patterns of crosslinks were observed for both crosslinkers (Figure 7G). Interestingly, a number of crosslinks were identified between the BRCT domain and the

Figure 6. Mapping the microtubule-binding interface of KKT4

(A) Microtubule co-sedimentation assay of 6His-$KKT4^{115–343}$ charge-reversal mutants, showing that mutations in the N-terminal basic coiled-coil region reduce the microtubule-binding affinity. The mutants that show ~50% reduction in the microtubule binding are indicated with an asterisk (*). S and P correspond to supernatant and pellet fractions, respectively.

(B) Histogram showing the ratio between bound and unbound fraction for different mutants of $KKT4^{115–343}$ using gels shown in (A). The mutations located in the domain N terminus show the most severe defects in microtubule binding.

CellPress
OPEN ACCESS

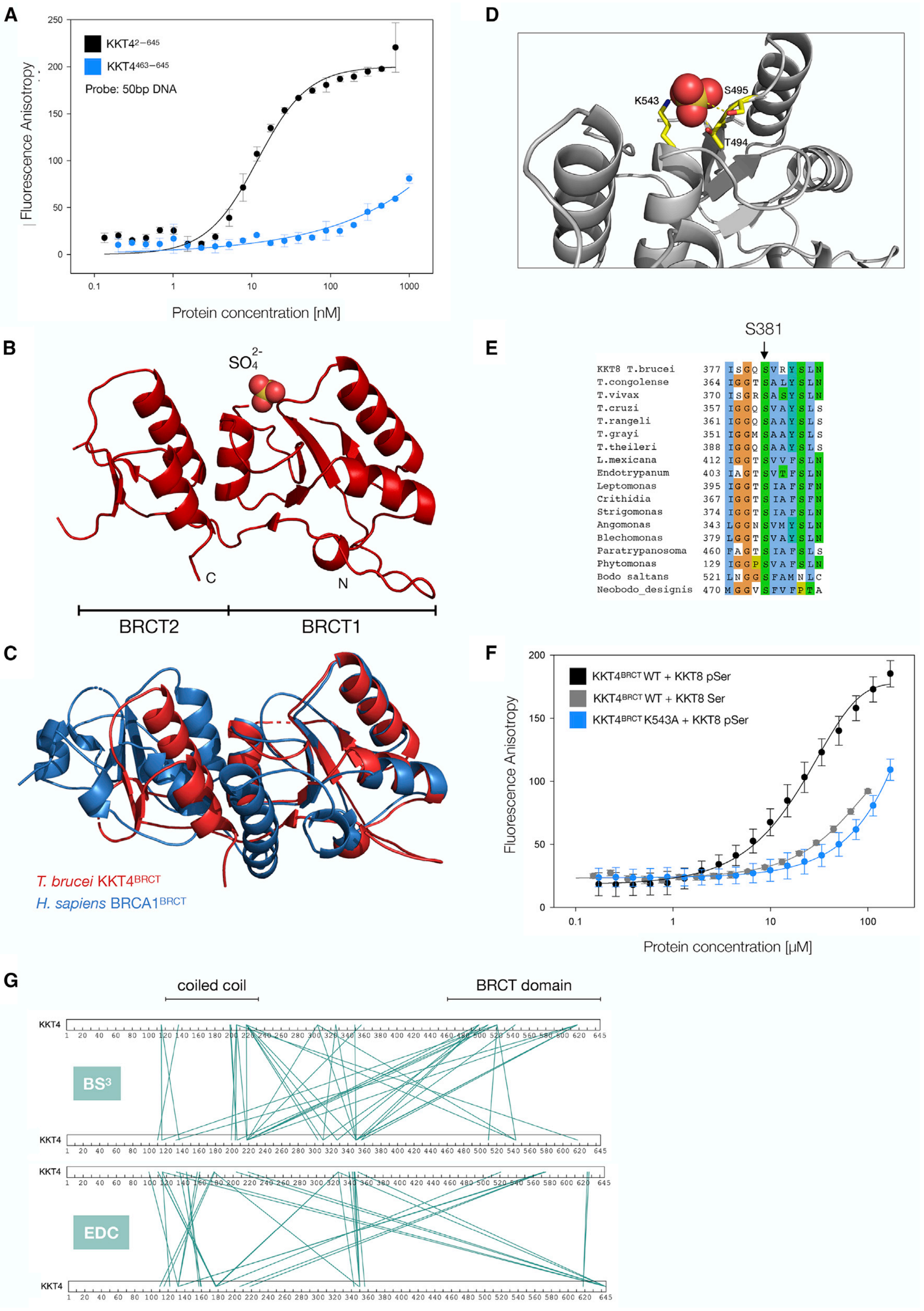

Figure 7. KKT4 BRCT domain is a phosphopeptide-binding domain

(A) Measured anisotropy is plotted against KKT4$^{2–645}$ and KKT4$^{463–645}$ protein concentrations in the fluorescence anisotropy assay using a 50-bp DNA probe, showing that full-length KKT4, but not the BRCT domain, binds DNA. The DNA sequence (~36% GC content) used in this assay is part of the centromeric

(legend continued on next page)

microtubule-binding domain using both BS^3 (K115/K499, K115/K521, K115/K543, K132/K618, K199/K521, K206/K521, K218/K499, K218/K510, K218/K521, K218/K618) and EDC/Sulfo-NHS (K115/D645, K132/E575, K132/D645, E178/K521, K204/D645, K206/E573, K206/D645 K218/E573, K218/D645). These results suggested that the KKT4 BRCT domain interacts with the microtubule-binding domain.

To confirm this, we monitored the effect of adding unlabeled KKT4 BRCT domain (Figures S8A and S8B) to ^{15}N-KKT4$^{115–232}$ using 2D NMR. Several residue-specific chemical shifts changes were observed, allowing identification of the BRCT interaction site on KKT4$^{115–232}$ (Figure S8B). The largest perturbations are observed for residues 115–123 (Figures S8A and S8B). It is interesting that one of the perturbed residues, K115, crosslinked with K543 from the BRCT domain. We repeated the experiment with a shorter KKT4 construct, KKT4$^{115–174}$, and obtained similar results (Figure S8B). Although crosslinks were also observed between the BRCT domain and residues E178, K199, K204, K206, and K218, these residues did not give observable peaks in the spectra of KKT4$^{115–232}$, so we could not confirm the interaction by NMR using KKT4$^{115–232}$. When similar experiments were performed with ^{15}N-KKT4$^{145–232}$ and KKT4^{BRCT}, no significant changes in chemical shift were observed (Figure S8B). These results suggest that an interaction between the BRCT domain and the microtubule-binding domain of KKT4 involves residues at the N terminus of the microtubule-binding domain.

The observed interaction between the BRCT domain and the microtubule-binding domain could be a potential regulation mechanism for the microtubule-binding activity of KKT4. To test this possibility, we purified KKT4$^{115–645}$, which contains both domains. We found that KKT4$^{115–645}$ and KKT4$^{115–343}$ interacted with microtubules with a similar affinity (Figures S8C–S8E). This suggests that the BRCT domain does not influence the interaction between KKT4 and Taxol-stabilized microtubules, at least in this assay. In the future, it will be interesting to analyze whether the observed interaction can regulate other activities of KKT4.

DISCUSSION

Many kinetochore-localized microtubule-binding proteins, such as the Ndc80, Ska, and Dam1 complexes, SKAP/Astrin, CENP-E, CENP-F, MCAK, INCENP, XMAP215, and dyneins, have been characterized in other model organisms (Maiato et al., 2004; Foley and Kapoor, 2013; Musacchio and Desai, 2017). Besides folded domains, many microtubule-binding proteins have predicted disordered regions that enhance their binding affinity (Guimaraes et al., 2008; Friese et al., 2016; Volkov, 2020). It is noteworthy that the predicted disorder has not been experimentally confirmed in most cases. Similarly, KKT4 has a predicted disordered segment at the microtubule-binding domain C terminus, which is not sufficient to bind microtubules but enhances the binding affinity (Llauro et al., 2018). In this study, we used NMR to confirm that this region is indeed disordered and also found that the N-terminal half of the KKT4 microtubule-binding domain has an elongated coiled-coil fold (Figure 8). Although a number of kinetochore proteins have coiled coils, microtubule-binding domains are typically located elsewhere, such as the calponin-homology domain for Ndc80/Nuf2 (Wei et al., 2007; Ciferri et al., 2008). In the case of SKAP/Astrin, which also has predicted coiled coils, it has been shown that the coiled-coil segment is unable to interact with microtubules on its own and that microtubule binding requires the N-terminal disordered fragment (Friese et al., 2016). Our mutagenesis analysis suggested that, in *T. brucei*, KKT4 binds microtubules through the N-terminal basic surface of the coiled coil. In the future, it will be important to directly visualize the microtubule-binding interface using methods such as electron microscopy.

It remains unknown whether (and how) microtubule-binding activities of KKT4 are regulated. Interestingly, we found that the KKT4 BRCT domain interacts with the N-terminal part of the microtubule-binding domain but does not modulate the microtubule-binding activities of KKT4, at least in the absence of phosphorylation (Figure S8D,E). Alternatively, the observed interaction might regulate other activities of KKT4. KKT4 co-purifies with the APC/C subunits (Akiyoshi and Gull, 2014), so we speculate that the interaction between the BRCT domain and the microtubule-binding domain might be governed by the attachment status, which in turn controls APC/C activities and cell-cycle progression.

In other eukaryotes, the Aurora B kinase plays an important role in regulating kinetochore-microtubule attachment by phosphorylating microtubule-binding kinetochore proteins, including Ndc80 and the Ska complexes (Cheeseman et al., 2002; Tien et al., 2010; Chan et al., 2012; Redli et al., 2016). Although Aurora B is conserved in kinetoplastids, it remains unclear whether it

sequence (CIR147) in *T. brucei*. The K_D for KKT4$^{2–645}$ (11 nM) was calculated using non-linear regression using SigmaPlot (Monks, 2002); the fit is shown as a solid line. Error bars are standard deviations from three independent measurements.
(B) Ribbon representation of KKT4 BRCT domain (KKT4$^{463–645}$). The N and C termini are indicated by N and C, respectively. The residues for which the electron density was not visible are not shown.
(C) Superposition of the KKT4 BRCT domain (red, PDB: 6ZPK) with the BRCA1 BRCT domain (blue, PDB: 3FA2), highlighting the absence of a β strand and an α helix in the C terminus of KKT4 BRCT. The RMSD (1.11 Å) was calculated using the *super* function in PyMOL for the N-terminal domain only (Delano, 2002).
(D) Close-up view showing coordination of a sulfate ion by T494, S495, and K543 (side chains of these residues are shown as yellow sticks).
(E) Multiple sequence alignment of KKT8 from various kinetoplastids, showing the conservation of *T. brucei* S381.
(F) Fluorescence anisotropy assay showing the KKT4 BRCT domain binding to a KKT8 phosphopeptide (DICGISGQ(pS)VRYSLND) (KKT4BRCT wild type in black and K543A mutant in blue) and non-phosphorylated peptide (gray). The K_D (30 μM) for the wild-type BRCT domain was calculated using non-linear regression using SigmaPlot (Monks, 2002); the fits are shown as solid lines. Error bars are standard deviations from three independent measurements.
(G) Crosslinking mass spectrometry of full-length KKT4 using BS^3 and EDC/Sulfo-NHS. The green lines indicate pairs of crosslinked residues. For the purposes of clarity, two molecules of KKT4 are shown. Note that crosslinks between the top and bottom KKT4 do not necessarily mean that crosslinks formed in between two separate molecules because it was not possible to distinguish between inter- and intra-molecule crosslinks in this experiment (except for those inter-molecule crosslinks formed between the same residues). A complete list of identified crosslinks is shown in Tables S4 and S5.
See also Figure S8 and Table S3, S4 and S5.

CellPress
OPEN ACCESS

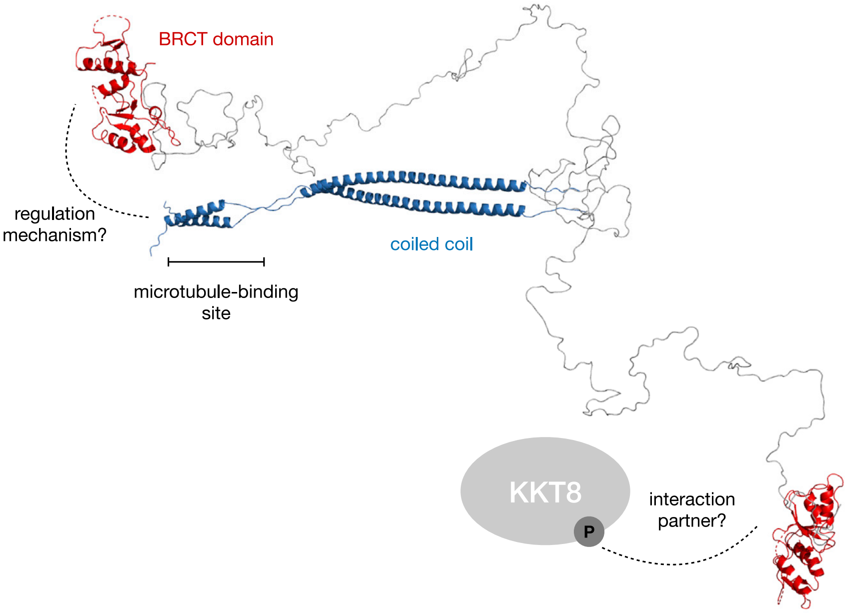

Figure 8. Structural and functional model of *T. brucei* KKT4$^{115–645}$

This model was generated using X-ray crystallography, NMR data, and modeling. The residues that are most important for the KKT4 microtubule-binding activity are located in the region indicated by the bar. The model shows two possible orientations of the BRCT domain (shown in red) with respect to the coiled coil (shown in blue) based on random conformations for the disordered region between Q233 and T473. These disordered conformations allow the BRCT domain to interact with the N-terminal region of the coiled coil. However, alternative conformations in which the BRCT domain is distant from the coiled coil are also possible, which might favor interactions with other kinetochore proteins such as phosphorylated KKT8 (shown as a gray ellipsoid).

regulates kinetochore-microtubule attachments (Tu et al., 2006). Our preliminary *in vitro* kinase assay failed to find evidence that *T. brucei* Aurora B phosphorylates KKT4 (data not shown). In contrast, we previously showed that KKT4 is phosphorylated by the KKT10 kinase, which localizes at kinetochores until the onset of anaphase and promotes the metaphase-to-anaphase transition (Ishii and Akiyoshi, 2020). A phospho-deficient KKT4 S477A mutant failed to rescue the growth defect caused by KKT4 RNAi (Ishii and Akiyoshi, 2020). Although the underlying molecular mechanism remains unknown, it is noteworthy that S477 is located just before the BRCT domain. In this study, we identified KKT8 as a putative binding partner for the KKT4 BRCT domain. It will be important to identify which kinase(s) phosphorylates the KKT8 S381 site to promote the interaction. Because kinetochore localization of the KKT10 kinase depends on the KKT8 complex (composed of KKT8, KKT9, KKT11, and KKT12) (Ishii and Akiyoshi, 2020), it is possible that KKT10's role in regulating the metaphase-to-anaphase transition is controlled by the KKT4-KKT8 interaction. These hypotheses will need to be tested in the future to better understand the mechanism of chromosome segregation in trypanosomes.

STAR★METHODS

Detailed methods are provided in the online version of this paper and include the following:

- KEY RESOURCES TABLE
- RESOURCE AVAILABILITY
  - Lead contact
  - Material availability
  - Data and code availability
- EXPERIMENTAL MODEL AND SUBJECT DETAILS
  - Bacterial culture
  - Cell culture
- METHOD DETAILS
  - Plasmids
  - Protein expression and purification
  - Expression and purification of isotopically labelled KKT4 fragments
  - Expression and purification of full length KKT4 from insect cells
  - Size Exclusion Chromatography with Multi-Angle Light Scattering (SEC-MALS)
  - Crystallization
  - Diffraction data collection and structure determination
  - NMR spectroscopy and analysis of NMR data
  - Modelling of *T. brucei* KKT4$^{115–232}$ and KKT4$^{115–645}$
  - Microtubule co-sedimentation assay
  - Fluorescence anisotropy assay
  - Chemical crosslinking mass spectrometry (XL-MS)
  - Interaction of the microtubule-binding and BRCT domains
  - Multiple sequence alignment
- QUANTIFICATION AND STATISTICAL ANALYSIS

SUPPLEMENTAL INFORMATION

Supplemental information can be found online at https://doi.org/10.1016/j.str.2021.04.004.

ACKNOWLEDGMENTS

We thank Krzysztof Kuś for his help with structural work and Richard Wheeler for providing *Leishmania mexicana* genomic DNA. We thank David Staunton for assistance with SEC-MALS experiments and Svenja Hester in the Advanced Proteomics Facility for processing mass spectrometry samples. We also thank Elspeth Garman, Midori Ishii Kanazawa, Hanako Hayashi, and Lucy Cornell for comments and suggestions on the manuscript. P.L. was supported by the Boehringer Ingelheim Fonds. B.A. was supported by a Wellcome Trust Senior Research Fellowship (grant no. 210622/Z/18/Z) and the European Molecular Biology Organisation Young Investigator Program. The Department of Biochemistry NMR Facility has benefitted from funding

provided by the Edward Penley Abraham Fund, the John Fell Fund and the Wellcome Trust.

AUTHOR CONTRIBUTIONS

P.L. purified recombinant proteins, solved crystal structures, and performed all experiments and data analysis. E.D.L. and G.M. assisted in solving crystal structures of KKT4$^{463–645}$ and *Lm*KKT4$^{184–284}$. S.M. analyzed crosslinking mass spectrometry data. P.L. and C.R. performed and analyzed NMR experiments. P.L., C.R., and B.A. designed experiments and wrote the manuscript.

DECLARATION OF INTERESTS

The authors declare no competing interests.

Received: October 21, 2020
Revised: February 17, 2021
Accepted: April 8, 2021
Published: April 28, 2021

REFERENCES

Abad, M.A., Medina, B., Santamaria, A., Zou, J., Plasberg-Hill, C., Madhumalar, A., Jayachandran, U., Redli, P.M., Rappsilber, J., Nigg, E.A., et al. (2014). Structural basis for microtubule recognition by the human kinetochore Ska complex. Nat. Commun. *5*, 2964. https://doi.org/10.1038/ncomms3964.

Akiyoshi, B., and Gull, K. (2014). Discovery of unconventional kinetochores in kinetoplastids. Cell *156*, 1247–1258. https://doi.org/10.1016/j.cell.2014.01.049.

Aslett, M., Aurrecoechea, C., Berriman, M., Brestelli, J., Brunk, B.P., Carrington, M., Depledge, D.P., Fischer, S., Gajria, B., Gao, X., et al. (2010). TriTrypDB: a functional genomic resource for the Trypanosomatidae. Nucleic Acids Res. *38*, 457–462. https://doi.org/10.1093/nar/gkp851.

Beger, R.D., and Bolton, P.H. (1997). Protein phi and psi dihedral restraints determined from multidimensional hypersurface correlations of backbone chemical shifts and their use in the determination of protein tertiary structures. J. Biomol. NMR *10*, 129–142. https://doi.org/10.1023/a:1018302105638.

Berriman, M., Ghedin, E., Hertz-Fowler, C., Blandin, G., Renauld, H., Bartholomeu, D.C., Lennard, N.J., Caler, E., Hamlin, N.E., Haas, B., et al. (2005). The genome of the African trypanosome *Trypanosoma brucei*. Science *309*, 416–422. https://doi.org/10.1126/science.1112642.

Biggins, S. (2013). The composition, functions, and regulation of the budding yeast kinetochore. Genetics *194*, 817–846. https://doi.org/10.1534/genetics.112.145276.

Brünger, A.T. (1992). X-PLOR, Version 3.1 : A System for X-Ray Crystallography and NMR (Yale University Press).

Brusini, L., D'Archivio, S., Mcdonald, J., and Wickstead, B. (2019). Ndc80/Nuf2-like protein KKIP1 connects a stable kinetoplastid outer kinetochore complex to the inner kinetochore and responds to metaphase tension. bioRxiv, 76482910.1101/764829.

Butenko, A., Opperdoes, F.R., Flegontova, O., Horak, A., Hampl, V., Keeling, P., Gawryluk, R.M.R., Tikhonenkov, D., Flegontov, P., and Lukes, J. (2020). Evolution of metabolic capabilities and molecular features of diplonemids, kinetoplastids, and euglenids. BMC Biol. *18*, https://doi.org/10.1186/s12915-020-0754-1.

Cavalier-Smith, T. (2010). Kingdoms Protozoa and Chromista and the eozoan root of the eukaryotic tree. Biol. Lett. *6*, 342–345. https://doi.org/10.1098/rsbl.2009.0948.

Chan, Y.W., Jeyaprakash, A.A., Nigg, E.A., and Santamaria, A. (2012). Aurora B controls kinetochore-microtubule attachments by inhibiting Ska complex-KMN network interaction. J. Cell Biol. *196*, 563–571. https://doi.org/10.1083/jcb.201109001.

Cheeseman, I.M. (2014). The kinetochore. Cold Spring Harb Perspect. Biol. *6*, a015826. https://doi.org/10.1101/cshperspect.a015826.

Cheeseman, I.M., Anderson, S., Jwa, M., Green, E.M., Kang, J., Yates, J.R., 3rd, Chan, C.S., Drubin, D.G., and Barnes, G. (2002). Phospho-regulation of kinetochore-microtubule attachments by the Aurora kinase Ipl1p. Cell *111*, 163–172. https://doi.org/10.1016/s0092-8674(02)00973-x.

Cheeseman, I.M., Brew, C., Wolyniak, M., Desai, A., Anderson, S., Muster, N., Yates, J.R., Huffaker, T.C., Drubin, D.G., and Barnes, G. (2001). Implication of a novel multiprotein Dam1p complex in outer kinetochore function. J. Cell Biol. *155*, 1137–1145. https://doi.org/10.1083/jcb.200109063.

Cheeseman, I.M., Chappie, J.S., Wilson-Kubalek, E.M., and Desai, A. (2006). The conserved KMN network constitutes the core microtubule-binding site of the kinetochore. Cell *127*, 983–997. https://doi.org/10.1016/j.cell.2006.09.039.

Cheeseman, I.M., and Desai, A. (2008). Molecular architecture of the kinetochore-microtubule interface. Nat. Rev. Mol. Cell Biol. *9*, 33–46. https://doi.org/10.1038/nrm2310.

Chen, Z.L., Meng, J.M., Cao, Y., Yin, J.L., Fang, R.Q., Fan, S.B., Liu, C., Zeng, W.F., Ding, Y.H., Tan, D., et al. (2019). A high-speed search engine pLink 2 with systematic evaluation for proteome-scale identification of cross-linked peptides. Nat. Commun. *10*, 3404. https://doi.org/10.1038/s41467-019-11337-z.

Ciferri, C., Pasqualato, S., Screpanti, E., Varetti, G., Santaguida, S., Dos Reis, G., Maiolica, A., Polka, J., De Luca, J.G., De Wulf, P., et al. (2008). Implications for kinetochore-microtubule attachment from the structure of an engineered Ndc80 complex. Cell *133*, 427–439. https://doi.org/10.1016/j.cell.2008.03.020.

Clapperton, J.A., Manke, I.A., Lowery, D.M., Ho, T., Haire, L.F., Yaffe, M.B., and Smerdon, S.J. (2004). Structure and mechanism of BRCA1 BRCT domain recognition of phosphorylated BACH1 with implications for cancer. Nat. Struct. Mol. Biol. *11*, 512–518. https://doi.org/10.1038/nsmb775.

Combe, C.W., Fischer, L., and Rappsilber, J. (2015). xiNET: cross-link network maps with residue resolution. Mol. Cell. Proteomics *14*, 1137–1147. https://doi.org/10.1074/mcp.O114.042259.

Cornilescu, G., Delaglio, F., and Bax, A. (1999). Protein backbone angle restraints from searching a database for chemical shift and sequence homology. J. Biomol. NMR *13*, 289–302. https://doi.org/10.1023/a:1008392405740.

Cornilescu, G., Marquardt, J.L., Ottiger, M., and Bax, A. (1998). Validation of protein structure from anisotropic carbonyl chemical shifts in a dilute liquid crystalline phase. J. Am. Chem. Soc. *120*, 6836–6837. https://doi.org/10.1021/ja9812610.

Cowtan, K. (2006). The Buccaneer software for automated model building. 1. Tracing protein chains. Acta Crystallogr. Section D: Biol. Crystallogr. *62*, 1002–1011. https://doi.org/10.1107/S0907444906022116.

D'archivio, S., and Wickstead, B. (2017). Trypanosome outer kinetochore proteins suggest conservation of chromosome segregation machinery across eukaryotes. J. Cell Biol. *216*, 379–391. https://doi.org/10.1083/jcb.201608043.

Delaglio, F., Grzesiek, S., Vuister, G.W., Zhu, G., Pfeifer, J., and Bax, A. (1995). NMRPipe: a multidimensional spectral processing system based on UNIX pipes. J. Biomol. NMR *6*, 277–293. https://doi.org/10.1007/bf00197809.

Delano, W.L. (2002). PyMOL: an open-source molecular graphics tool. CCP4 Newsl. Protein Crystallogr. *40*, 82–92.

Desai, A., and Mitchison, T.J. (1997). Microtubule polymerization dynamics. Annu. Rev. Cell Dev. Biol. *13*, 83–117. https://doi.org/10.1146/annurev.cellbio.13.1.83.

Emsley, P., Lohkamp, B., Scott, W.G., and Cowtan, K. (2010). Features and development of coot. Acta Crystallogr. D *66*, 486–501. https://doi.org/10.1107/S0907444910007493.

Foley, E.A., and Kapoor, T.M. (2013). Microtubule attachment and spindle assembly checkpoint signalling at the kinetochore. Nat. Rev. Mol. Cell Biol. *14*, 25–37. https://doi.org/10.1038/nrm3494.

Friese, A., Faesen, A.C., Huis in 'T Veld, P.J., Fischbock, J., Prumbaum, D., Petrovic, A., Raunser, S., Herzog, F., and Musacchio, A. (2016). Molecular requirements for the inter-subunit interaction and kinetochore recruitment of SKAP and Astrin. Nat. Commun. *7*, 11407. https://doi.org/10.1038/ncomms11407.

Gileadi, O., Burgess-Brown, N.A., Colebrook, S.M., Berridge, G., Savitsky, P., Smee, C.E.A., Loppnau, P., Johansson, C., Salah, E., and Pantic, N.H. (2008). High throughput production of recombinant human proteins for crystallography. Methods Mol. Biol. (Clifton, N.J.) *426*, 221–246. https://doi.org/10.1007/978-1-60327-058-8_14.

Guimaraes, G.J., Dong, Y., Mcewen, B.F., and Deluca, J.G. (2008). Kinetochore-microtubule attachment relies on the disordered N-terminal tail domain of Hec1. Curr. Biol. *18*, 1778–1784. https://doi.org/10.1016/j.cub.2008.08.012.

Hanisch, A., Sillje, H.H., and Nigg, E.A. (2006). Timely anaphase onset requires a novel spindle and kinetochore complex comprising Ska1 and Ska2. EMBO J. *25*, 5504–5515. https://doi.org/10.1038/sj.emboj.7601426.

Holm, L. (2019). Benchmarking fold detection by DaliLite v.5. Bioinformatics (Oxford, England) *35*, 5326–5327. https://doi.org/10.1093/bioinformatics/btz536.

Ishii, M., and Akiyoshi, B. (2020). Characterization of unconventional kinetochore kinases KKT10 and KKT19 in *Trypanosoma brucei*. J. Cell Sci. *133*, https://doi.org/10.1242/jcs.240978.

Jurrus, E., Engel, D., Star, K., Monson, K., Brandi, J., Felberg, L.E., Brookes, D.H., Wilson, L., Chen, J., Liles, K., et al. (2018). Improvements to the APBS biomolecular solvation software suite. Protein Sci. *27*, 112–128. https://doi.org/10.1002/pro.3280.

Katoh, K., Rozewicki, J., and Yamada, K.D. (2019). MAFFT online service: multiple sequence alignment, interactive sequence choice and visualization. Brief Bioinform *20*, 1160–1166. https://doi.org/10.1093/bib/bbx108.

Kay, L.E., Torchia, D.A., and Bax, A. (1989). Backbone dynamics of proteins as studied by ^{15}N inverse detected heteronuclear NMR spectroscopy: application to staphylococcal nuclease. Biochemistry *28*, 8972–8979. https://doi.org/10.1021/bi00449a003.

Kern, D.M., Monda, J.K., Su, K.C., Wilson-Kubalek, E.M., and Cheeseman, I.M. (2017). Astrin-SKAP complex reconstitution reveals its kinetochore interaction with microtubule-bound Ndc80. eLife *610*, https://doi.org/10.7554/eLife.26866.

Leitner, A., Faini, M., Stengel, F., and Aebersold, R. (2016). Crosslinking and mass spectrometry: an integrated technology to understand the structure and function of molecular machines. Trends Biochem. Sci. *41*, 20–32. https://doi.org/10.1016/j.tibs.2015.10.008.

Lescop, E., Schanda, P., and Brutscher, B. (2007). A set of BEST triple-resonance experiments for time-optimized protein resonance assignment. J. Magn. Reson. *187*, 163–169. https://doi.org/10.1016/j.jmr.2007.04.002.

Leung, C.C., and Glover, J.N. (2011). BRCT domains: easy as one, two, three. Cell Cycle *10*, 2461–2470. https://doi.org/10.4161/cc.10.15.16312.

Liebschner, D., Afonine, P.V., Baker, M.L., Bunkoczi, G., Chen, V.B., Croll, T.I., Hintze, B., Hung, L.W., Jain, S., Mccoy, A.J., et al. (2019). Macromolecular structure determination using X-rays, neutrons and electrons: recent developments in Phenix. Acta Crystallogr. D Struct. Biol. *75*, 861–877. https://doi.org/10.1107/S2059798319011471.

Linding, R., Jensen, L.J., Diella, F., Bork, P., Gibson, T.J., and Russell, R.B. (2003). Protein disorder prediction: implications for structural proteomics. Structure *11*, 1453–1459. https://doi.org/10.1016/j.str.2003.10.002.

Llauro, A., Hayashi, H., Bailey, M.E., Wilson, A., Ludzia, P., Asbury, C.L., and Akiyoshi, B. (2018). The kinetoplastid kinetochore protein KKT4 is an unconventional microtubule tip-coupling protein. J. Cell Biol. *217*, 3886–3900. https://doi.org/10.1083/jcb.201711181.

Ludzia, P., Akiyoshi, B., and Redfield, C. (2020). (1)H, (13)C and (15)N resonance assignments for the microtubule-binding domain of the kinetoplastid kinetochore protein KKT4 from *Trypanosoma brucei*. Biomol. NMR Assign. *14*, 309–315. https://doi.org/10.1007/s12104-020-09968-1.

Lupas, A., Van Dyke, M., and Stock, J. (1991). Predicting coiled coils from protein sequences. Science *252*, 1162–1164. https://doi.org/10.1126/science.252.5009.1162.

Mackay, J.P., Shaw, G.L., and King, G.F. (1996). Backbone dynamics of the c-jun leucine Zipper: ^{15}N NMR relaxation studies. Biochemistry *35*, 4867–4877. https://doi.org/10.1021/bi952761y.

Maiato, H., Deluca, J., Salmon, E.D., and Earnshaw, W.C. (2004). The dynamic kinetochore-microtubule interface. J. Cell Sci. *117*, 5461–5477. https://doi.org/10.1242/jcs.01536.

Manke, I.A., Lowery, D.M., Nguyen, A., and Yaffe, M.B. (2003). BRCT repeats as phosphopeptide-binding modules involved in protein targeting. Science *302*, 636–639. https://doi.org/10.1126/science.1088877.

Marsh, J.A., Singh, V.K., Jia, Z., and Forman-Kay, J.D. (2006). Sensitivity of secondary structure propensities to sequence differences between alpha- and gamma-synuclein: implications for fibrillation. Protein Sci. *15*, 2795–2804. https://doi.org/10.1110/ps.062465306.

Mattson, G., Conklin, E., Desai, S., Nielander, G., Savage, M.D., and Morgensen, S. (1993). A practical approach to crosslinking. Mol. Biol. Rep. *17*, 167–183. https://doi.org/10.1007/BF00986726.

Mcdonnell, A.V., Jiang, T., Keating, A.E., and Berger, B. (2006). Paircoil2: improved prediction of coiled coils from sequence. Bioinformatics *22*, 356–358. https://doi.org/10.1093/bioinformatics/bti797.

Mcintosh, J.R. (2016). Mitosis. Cold Spring Harb Perspect. Biol. *8*, https://doi.org/10.1101/cshperspect.a023218.

Meraldi, P., Mcainsh, A.D., Rheinbay, E., and Sorger, P.K. (2006). Phylogenetic and structural analysis of centromeric DNA and kinetochore proteins. Genome Biol. *7*, R23. https://doi.org/10.1186/gb-2006-7-3-r23.

Monks, S. (2002). SigmaPlot 8.0. Biotech. Softw. Internet Rep. *3*, 141–145. https://doi.org/10.1089/152791602321105816.

Musacchio, A., and Desai, A. (2017). A molecular view of kinetochore assembly and function. Biology (Basel) *6*, https://doi.org/10.3390/biology6010005.

Nerusheva, O.O., and Akiyoshi, B. (2016). Divergent polo box domains underpin the unique kinetoplastid kinetochore. Open Biol. *6*, https://doi.org/10.1098/rsob.150206.

Nerusheva, O.O., Ludzia, P., and Akiyoshi, B. (2019). Identification of four unconventional kinetoplastid kinetochore proteins KKT22-25 in *Trypanosoma brucei*. Open Biol. *9*, 190236. https://doi.org/10.1098/rsob.190236.

Nicklas, R.B. (1997). How cells get the right chromosomes. Science *275*, 632–637. https://doi.org/10.1126/science.275.5300.632.

Obado, S.O., Bot, C., Nilsson, D., Andersson, B., and Kelly, J.M. (2007). Repetitive DNA is associated with centromeric domains in *Trypanosoma brucei* but not *Trypanosoma cruzi*. Genome Biol. *8*, https://doi.org/10.1186/gb-2007-8-3-r37.

Perez-Riverol, Y., Csordas, A., Bai, J., Bernal-Llinares, M., Hewapathirana, S., Kundu, D.J., Inuganti, A., Griss, J., Mayer, G., Eisenacher, M., et al. (2019). The PRIDE database and related tools and resources in 2019: improving support for quantification data. Nucleic Acids Res. *47*, 442–450. https://doi.org/10.1093/nar/gky1106.

Potter, S.C., Luciani, A., Eddy, S.R., Park, Y., Lopez, R., and Finn, R.D. (2018). HMMER web server: 2018 update. Nucleic Acids Res. *46*, W200–W204. https://doi.org/10.1093/nar/gky448.

Redli, P.M., Gasic, I., Meraldi, P., Nigg, E.A., and Santamaria, A. (2016). The Ska complex promotes Aurora B activity to ensure chromosome biorientation. J. Cell Biol. *215*, 77–93. https://doi.org/10.1083/jcb.201603019.

Rodríguez, D., Sammito, M., Meindl, K., De Ilarduya, Ĩ.M., Potratz, M., Sheldrick, G.M., and Usón, I. (2012). Practical structure solution with ARCIMBOLDO. Acta Crystallogr. Section D Biol. Crystallogr. *68*, 336–343. https://doi.org/10.1107/S0907444911056071.

Rodríguez, D.D., Grosse, C., Himmel, S., González, C., De Ilarduya, I.M., Becker, S., Sheldrick, G.M., and Usón, I. (2009). Crystallographic ab initio protein structure solution below atomic resolution. Nat. Methods *6*, 651–653. https://doi.org/10.1038/nmeth.1365.

Rossi, A.M., and Taylor, C.W. (2011). Analysis of protein-ligand interactions by fluorescence polarization. Nat. Protoc. *6*, 365–387. https://doi.org/10.1038/nprot.2011.305.

Rückert, M., and Otting, G. (2000). Alignment of biological macromolecules in novel nonionic liquid crystalline media for NMR experiments. J. Am. Chem. Soc. *122*, 7793–7797.

Schmidt, J.C., Arthanari, H., Boeszoermenyi, A., Dashkevich, N.M., Wilson-Kubalek, E.M., Monnier, N., Markus, M., Oberer, M., Milligan, R.A., Bathe,

M., et al. (2012). The kinetochore-bound Ska1 complex tracks depolymerizing microtubules and binds to curved protofilaments. Dev. Cell *23*, 968–980. https://doi.org/10.1016/j.devcel.2012.09.012.

Schneider, C.A., Rasband, W.S., and Eliceiri, K.W. (2012). NIH Image to ImageJ: 25 years of image analysis. Nat. Methods *9*, 671–675. https://doi.org/10.1038/nmeth.2089.

Schnell, J.R., Zhou, G.P., Zweckstetter, M., Rigby, A.C., and Chou, J.J. (2005). Rapid and accurate structure determination of coiled-coil domains using NMR dipolar couplings: application to cGMP-dependent protein kinase Ialpha. Protein Sci. *14*, 2421–2428. https://doi.org/10.1110/ps.051528905.

Schulte-Herbruggen, T., and Sorensen, O.W. (2000). Clean TROSY: compensation for relaxation-induced artifacts. J. Magn. Reson. *144*, 123–128. https://doi.org/10.1006/jmre.2000.2020.

Shen, Y., and Bax, A. (2013). Protein backbone and sidechain torsion angles predicted from NMR chemical shifts using artificial neural networks. J. Biomol. NMR *56*, 227–241. https://doi.org/10.1007/s10858-013-9741-y.

Shiozaki, E.N., Gu, L., Yan, N., and Shi, Y. (2004). Structure of the BRCT repeats of BRCA1 bound to a BACH1 phosphopeptide: implications for signaling. Mol. Cell *14*, 405–412. https://doi.org/10.1016/s1097-2765(04)00238-2.

Spera, S., and Bax, A. (1991). Empirical correlation between protein backbone conformation and C.alpha. and C.beta. ^{13}C nuclear magnetic resonance chemical shifts. J. Am. Chem. Soc. *113*, 5490–5492. https://doi.org/10.1021/ja00014a071.

Strelkov, S.V., and Burkhard, P. (2002). Analysis of alpha-helical coiled coils with the program TWISTER reveals a structural mechanism for stutter compensation. J. Struct. Biol. *137*, 54–64. https://doi.org/10.1006/jsbi.2002.4454.

Strong, M., Sawaya, M.R., Wang, S., Phillips, M., Cascio, D., and Eisenberg, D. (2006). Toward the structural genomics of complexes: crystal structure of a PE/PPE protein complex from *Mycobacterium tuberculosis*. Proc. Natl. Acad. Sci. U S A. *103*, 8060–8065. https://doi.org/10.1073/pnas.0602606103.

Tien, J.F., Umbreit, N.T., Gestaut, D.R., Franck, A.D., Cooper, J., Wordeman, L., Gonen, T., Asbury, C.L., and Davis, T.N. (2010). Cooperation of the Dam1 and Ndc80 kinetochore complexes enhances microtubule coupling and is regulated by aurora B. J. Cell Biol. *189*, 713–723. https://doi.org/10.1083/jcb.200910142.

Tu, X., Kumar, P., Li, Z., and Wang, C.C. (2006). An aurora kinase homologue is involved in regulating both mitosis and cytokinesis in *Trypanosoma brucei*. J. Biol. Chem. *281*, 9677–9687. https://doi.org/10.1074/jbc.M511504200.

Uniprot, C. (2019). UniProt: a worldwide hub of protein knowledge. Nucleic Acids Res. *47*, D506–D515. https://doi.org/10.1093/nar/gky1049.

Van Hooff, J.J., Tromer, E., Van Wijk, L.M., Snel, B., and Kops, G.J. (2017). Evolutionary dynamics of the kinetochore network in eukaryotes as revealed by comparative genomics. EMBO Rep. *18*, 1559–1571. https://doi.org/10.15252/embr.201744102.

Volkov, V.A. (2020). Microtubules pull the strings: disordered sequences as efficient couplers of microtubule-generated force. Essays Biochem. https://doi.org/10.1042/EBC2019007810.1042/EBC20190078.

Vranken, W.F., Boucher, W., Stevens, T.J., Fogh, R.H., Pajon, A., Llinas, M., Ulrich, E.L., Markley, J.L., Ionides, J., and Laue, E.D. (2005). The CCPN data model for NMR spectroscopy: development of a software pipeline. Proteins *59*, 687–696. https://doi.org/10.1002/prot.20449.

Waterhouse, A.M., Procter, J.B., Martin, D.M.A., Clamp, M., and Barton, G.J. (2009). Jalview Version 2-A multiple sequence alignment editor and analysis workbench. Bioinformatics *25*, 1189–1191. https://doi.org/10.1093/bioinformatics/btp033.

Webb, B., and Sali, A. (2016). Comparative protein structure modeling using MODELLER. Curr. Protoc. Bioinformatics *54*, 5.6.1–5.6.30. https://doi.org/10.1002/cpbi.3.

Wei, R.R., Al-Bassam, J., and Harrison, S.C. (2007). The Ndc80/HEC1 complex is a contact point for kinetochore-microtubule attachment. Nat. Struct. Mol. Biol. *14*, 54–59. https://doi.org/10.1038/nsmb1186.

Wen, J., Arakawa, T., and Philo, J.S. (1996). Size-exclusion chromatography with on-line light-scattering, absorbance, and refractive index detectors for studying proteins and their interactions. Anal. Biochem. *240*, 155–166. https://doi.org/10.1006/abio.1996.0345.

Williams, R.S., Lee, M.S., Hau, D.D., and Glover, J.N. (2004). Structural basis of phosphopeptide recognition by the BRCT domain of BRCA1. Nat. Struct. Mol. Biol. *11*, 519–525. https://doi.org/10.1038/nsmb776.

Wishart, D.S., Sykes, B.D., and Richards, F.M. (1991). Simple techniques for the quantification of protein secondary structure by 1H NMR spectroscopy. FEBS Lett. *293*, 72–80. https://doi.org/10.1016/0014-5793(91)81155-2.

Yu, X., Chini, C.C.S., He, M., Mer, G., and Chen, J. (2003). The BRCT domain is a phospho-protein binding domain. Science *302*, 639–642. https://doi.org/10.1126/science.1088753.

Zhang, X., Moréra, S., Bates, P.A., Whitehead, P.C., Coffer, A.I., Hainbucher, K., Nash, R.A., Sternberg, M.J.E., Lindahl, T., and Freemont, P.S. (1998). Structure of an XRCC1 BRCT domain: a new protein-protein interaction module. EMBO J. *17*, 6404–6411. https://doi.org/10.1093/emboj/17.21.6404.

Zhu, G., Xia, Y., Nicholson, L.K., and Sze, K.H. (2000). Protein dynamics measurements by TROSY-based NMR experiments. J. Magn. Reson. *143*, 423–426. https://doi.org/10.1006/jmre.2000.2022.

STAR★METHODS

KEY RESOURCES TABLE

REAGENT or RESOURCE	SOURCE	IDENTIFIER
Chemicals, peptides, and recombinant proteins		
1-Hexanol	Sigma-Aldrich	H1,330-3
Ammonium Chloride (^{15}N, 99%)	Goss Scientific	NLM-467
Anti-FLAG M2 affinity gel	Sigma-Aldrich	A2220
Benzonase Nuclease	Sigma-Aldrich	E1014
BS3-d0	Thermo Fisher	SK256507
Cellfectin II	Invitrogen	10362-100
D-Glucose ($^{13}C_6$, 99%)	Goss Scientific	CLM-1396
Deuterium oxide	Sigma-Aldrich	151882
E-64	Peptide Institute Inc.	4096
EDC	Thermo Fisher	PG82079
EDTA	Sigma-Aldrich	324503
EGTA	Sigma-Aldrich	E3889
3xFLAG peptide	Sigma-Aldrich	F4799
Glycerol	Sigma-Aldrich	G5516
Heparin 1 ml chromatography column	GE-Healthcare	17-0406-01
HEPES	Sigma-Aldrich	H3375
Hexaethylene glycol monododecyl ether (C12E6)	Sigma-Aldrich	52044
HiLoad 16/600 Superdex 200 pg	GE-Healthcare	28-9893-35
HiLoad 16/600 Superdex 75 pg	GE-Healthcare	28-9893-33
Imidazole	Sigma-Aldrich	56750
Index crystallisation screen	Hampton Research	HR2-134
KCl	Sigma-Aldrich	P9541
Leupeptin	EMD Millipore Corp.	3158107
$MgCl_2$	Sigma-Aldrich	M8266
Morpheus II HT-96 crystallisation screen	Molecular Dimensions	MD1-92
Na_2HPO_4	Sigma-Aldrich	S0876
NaCl	Sigma-Aldrich	S9888
NaH_2PO_4	Sigma-Aldrich	S0751
Pepstatin A	EMD Millipore Corp.	516481
PIPES	Sigma-Aldrich	P6757
PMSF	Sigma-Aldrich	P7626
Porcine brain tubulin	Cytoskeleton, Inc	T240
ProPlex crystallisation screen	Molecular Dimensions	MD1-42
Resource S 6 ml cation exchange chromatography column	GE-Healthcare	17-1180-01
Sf-900 II SFM media	Invitrogen	10902104
SimplyBlue Safe Stain	Invitrogen	46-5034
Sulfo-NHS	Thermo Fisher	24510
Superdex 200 10/300	GE-Healthcare	17-5175-01
Superose 6 10/300	GE-Healthcare	17-5172-01
Synthetic KKT8 phosphopeptide (BA_peptide_2 (DBS1831-1)): 5-FAM/DDICGISGQ(pSer)VRYSLND-NH_2	Designer Bioscience, Cambridge	N/A
Synthetic KKT8 peptide (BA_peptide_5 (DBS1838-1)): 5-FAM/DDICGISGQSVRYSLND-NH_2	Designer Bioscience, Cambridge	N/A

(Continued on next page)

Continued

REAGENT or RESOURCE	SOURCE	IDENTIFIER
TALON Metal Affinity Resin	Takara	635503
TCEP	Sigma-Aldrich	C4706
Zeba Spin Desalting Columns, 7K MWCO, 5 ml	Thermo Fisher	89891
Deposited data		
Trypanosoma cruzi KKT4$^{117–218}$ crystal structure	This study	PDB: 6ZPM
Leishmania mexicana KKT4$^{184–284}$ crystal structure	This study	PDB: 6ZPJ
Trypanosoma brucei KKT4$^{463–645}$ crystal structure	This study	PDB: 6ZPK
KKT4$^{115–343}$ chemical shifts	(Ludzia et al., 2020)	BMRB: 50229
KKT4$^{115–174}$ chemical shifts	(Ludzia et al., 2020)	BMRB: 50215
KKT4$^{145–232}$ chemical shifts	(Ludzia et al., 2020)	BMRB: 50228
Crosslinking mass spectrometry raw data	This study	PRIDE: PDXD020229
Experimental models: organisms/strains		
Escherichia coli: BL21(DE3)	Novagen	69450
Spodoptera frugiperda: Sf9	Thermo Fisher	12659017
Recombinant DNA		
Synthetic dsDNA (BA3098): 6-FAM/ CAATATGTAAGGTGTTTTGGTGTAA AACACGCATTCTTGCATAACATGCA	Custom synthesis by Integrated DNA Technologies, Inc	N/A
Plasmid: pRSFDuet-1	Novagen	71341
Plasmid: pNIC28-Bsa4	(Gileadi et al., 2008)	N/A
Plasmid: pACEBac1	Geneva Biotech	MultiBac
Plasmid: pACEBac2	Geneva Biotech	MultiBac
Plasmid: pIDK	Geneva Biotech	MultiBac
Plasmid: pIDS	Geneva Biotech	MultiBac
Plasmid: KKT15 in pIDK (pBA336)	This study	N/A
Plasmid: KKT14 in pIDK (pBA485)	This study	N/A
Plasmid: KKT14, KKT15 in pIDK (pBA515)	This study	N/A
Plasmid: 3FLAG-KKT4 in pACEBac2 (pBA818)	(Ishii and Akiyoshi, 2020)	N/A
Bacmid: 3FLAG-KKT4 (pBA826)	(Ishii and Akiyoshi, 2020)	N/A
Plasmid: SNAP-6HIS-3FLAG-KKT4 (codon optimised for expression in *Spodoptera frugiperda*) in pACEBac2 (pBA925)	(Llauro et al., 2018)	N/A
Plasmid: 6HIS-KKT4$^{115–645}$ (pBA987)	This study	N/A
Plasmid: 6HIS-KKT4$^{115–343}$ (pBA1065)	(Llauro et al., 2018)	N/A
Synthesised gene: KKIP1 (codon optimised for expression in *Spodoptera frugiperda*) (pBA1166)	This study	N/A
Plasmid: 6HIS-KKT4$^{115–174}$ (pBA1171)	(Llauro et al., 2018)	N/A
Plasmid: KKIP1 (codon optimised for expression in *Spodoptera frugiperda*) in pIDS (pBA1207)	This study	N/A
Plasmid: 6HIS-KKT4$^{115–343\ R123E}$ (pBA1328)	This study	N/A
Plasmid: 6HIS-KKT4$^{115–343\ K132E}$ (pBA1329)	This study	N/A
Plasmid: 6HIS-KKT4$^{115–343\ K154E}$ (pBA1330)	This study	N/A
Plasmid: 6HIS-KKT4$^{101–352}$ (pBA1393)	(Llauro et al., 2018)	N/A
Plasmid: 3FLAG-KKT4, KKT14, KKT15 in pACEBac2 (pBA1371)	This study	N/A
Bacmid: 3FLAG-KKT4, KKT14, KKT15 (pBA1388)	This study	N/A
Plasmid: 6HIS-KKT4$^{2–114}$ (pBA1413)	(Llauro et al., 2018)	N/A
Plasmid: 6HIS-KKT4$^{145–232}$ (pBA1441)	This study	N/A

(*Continued on next page*)

CellPress
OPEN ACCESS

Continued

REAGENT or RESOURCE	SOURCE	IDENTIFIER
Plasmid: KKIP1 (codon optimised for expression in *Spodoptera frugiperda*) in pACEBac1 (pBA1469)	This study	N/A
Plasmid: 6HIS-KKT4$^{463–645}$ (pBA1513)	(Ishii and Akiyoshi, 2020)	N/A
Bacmid: KKIP1 (pBA1540)	This study	N/A
Plasmid: 6HIS-KKT4$^{115–232}$ (pBA1601)	This study	N/A
Plasmid: 6HIS-*Lm*KKT4$^{184–284}$ (pBA1618)	This study	N/A
Plasmid: 6HIS-*Tc*KKT4$^{117–328}$ (codon optimised for expression in *E. coli*) (pBA1753)	(Llauro et al., 2018)	N/A
Plasmid: 6HIS-KKT4$^{115–343\ R167D}$ (pBA2036)	This study	N/A
Plasmid: 6HIS-KKT4$^{115–343\ K218E}$ (pBA2037)	This study	N/A
Plasmid: 6HIS-KKT4$^{115–343\ R230D}$ (pBA2038)	This study	N/A
Plasmid: 6HIS-KKT4$^{115–343\ K204E}$ (pBA2042)	This study	N/A
Plasmid: 6HIS-KKT4$^{115–343\ R145D}$ (pBA2043)	This study	N/A
Plasmid: 6HIS-KKT4$^{115–343\ K166E}$ (pBA2044)	This study	N/A
Plasmid: 6HIS-KKT4$^{115–343\ K206E}$ (pBA2045)	This study	N/A
Plasmid: 6HIS-*Tc*KKT4$^{117–218}$ (codon optimised for expression in *E. coli*) (pBA2151)	This study	N/A
Plasmid: 6HIS-KKT4$^{115–343\ R126D}$ (pBA2244)	This study	N/A
Plasmid: 6HIS-KKT4$^{115–343\ R130D}$ (pBA2245)	This study	N/A
Plasmid: 6HIS-KKT4$^{115–343\ K136E}$ (pBA2246)	This study	N/A
Plasmid: 6HIS-KKT4$^{115–343\ R140D}$ (pBA2247)	This study	N/A
Plasmid: 6HIS-KKT4$^{115–343\ R141D}$ (pBA2248)	This study	N/A
Plasmid: 6HIS-KKT4$^{115–343\ K144E}$ (pBA2249)	This study	N/A
Plasmid: 6HIS-KKT4$^{115–343\ K115E}$ (pBA2252)	This study	N/A
Plasmid: 6HIS-KKT4$^{115–343\ R164D}$ (pBA2253)	This study	N/A
Plasmid: 6HIS-KKT4$^{115–343\ K198E}$ (pBA2254)	This study	N/A
Plasmid: 6HIS-KKT4$^{115–343\ R217D}$ (pBA2255)	This study	N/A
Plasmid: 6HIS-KKT4$^{115–343\ K179E}$ (pBA2256)	This study	N/A
Plasmid: 6HIS-KKT4$^{115–343\ K199E}$ (pBA2257)	This study	N/A
Plasmid: 6HIS-KKT4$^{463–645\ K543A}$ (pBA2264)	This study	N/A
Plasmid: 6HIS-KKT4$^{233–343}$ (pBA2380)	This study	N/A
Software and algorithms		
ARCIMBOLDO LITE	(Rodríguez et al., 2009)	http://www.ccp4.ac.uk/
ASTRA	Wyatt Technology	https://store.wyatt.com/shop/viscostar/viscostar-iii/astra-software/
Buccaneer	(Cowtan, 2006)	http://www.ccp4.ac.uk/
CCPNmr	(Vranken et al., 2005)	https://www.ccpn.ac.uk
COILS server	Lupas et al., 1991	https://embnet.vital-it.ch/software/COILS_form.html
COOT	(Emsley et al., 2010)	http://www2.mrc-lmb.cam.ac.uk/Personal/pemsley/coot/
DALI server	(Holm, 2019)	http://ekhidna2.biocenter.helsinki.fi/dali/
Diffraction Anisotropy Server	(Strong et al., 2006)	http://services.mbi.ucla.edu/anisoscale/
DisEMBL	(Linding et al., 2003)	http://dis.embl.de
HMMER web server	(Potter et al., 2018)	https://www.ebi.ac.uk/Tools/hmmer/
ImageJ	(Schneider et al., 2012)	https://imagej.nih.gov/ij/
Jalview	(Waterhouse et al., 2009)	http://www.jalview.org/
MAFFT	(Katoh et al., 2019)	https://mafft.cbrc.jp/alignment/server/
MODELLER	(Webb and Sali, 2016)	https://salilab.org/modeller/

(*Continued on next page*)

Continued

REAGENT or RESOURCE	SOURCE	IDENTIFIER
NMRPipe	(Delaglio et al., 1995)	https://spin.niddk.nih.gov/NMRPipe/
PHENIX	(Liebschner et al., 2019)	http://www.phenix-online.org/
Paircoil2	(Mcdonnell et al., 2006)	http://cb.csail.mit.edu/cb/paircoil2/
pLink	(Chen et al., 2019)	http://pfind.ict.ac.cn/software/pLink1/index.html
PRIDE database	(Perez-Riverol et al., 2019)	http://www.proteomexchange.org
PyMOL	(Delano, 2002)	http://www.pymol.org/
SigmaPlot	(Monks, 2002)	https://systatsoftware.com/products/sigmaplot/
SSP	(Marsh et al., 2006)	http://pound.med.utoronto.ca/software.html
TALOS-N	(Shen and Bax, 2013)	https://spin.niddk.nih.gov/bax/software/TALOS-N/
TOPSPIN 3.2	Bruker Biospin	https://www.bruker.com/service/support-upgrades/software-downloads/nmr.html
TriTryp database	(Aslett et al., 2010)	https://tritrypdb.org
TWISTER	(Strelkov and Burkhard, 2002)	https://pharm.kuleuven.be/apps/biocryst/twister.php
	(UniProt, 2019)	https://www.uniprot.org
xiNET	(Combe et al., 2015)	http://crosslinkviewer.org
X-PLOR	(Brünger, 1992)	Version 3.1

RESOURCE AVAILABILITY

Lead contact

Further information and requests for resources and reagents should be directed to and will be fulfilled by the Lead contact, Bungo Akiyoshi (bungo.akiyoshi@bioch.ox.ac.uk).

Material availability

Plasmids generated in the course of this study can be requested from the Lead contact, Bungo Akiyoshi (bungo.akiyoshi@bioch.ox.ac.uk).

Data and code availability

Data generated during this study are included in the manuscript and supplemental information. Protein coordinates have been deposited in the RCSB Protein Data Bank (http://www.rcsb.org/) with accession codes PDB: 6ZPM (*Trypanosoma cruzi* Sylvio X10 KKT4$^{117–218}$), PDB: 6ZPJ (*Leishmania mexicana* KKT4$^{184–284}$) and PDB: 6ZPK (*Trypanosoma brucei* KKT4463–645). The chemical shift assignments for KKT4 have been deposited in the BioMagResBank (http://www.bmrb.wisc.edu) under the accession numbers 50215 (*Trypanosoma brucei* KKT4$^{115–174}$), 50228 (*Trypanosoma brucei* KKT4$^{145–232}$) and 50229 (*Trypanosoma brucei* KKT4$^{115–343}$). All raw files relating to crosslinking mass-spectrometry have been deposited to the ProteomeXchange Consortium via the PRIDE partner repository (Perez-Riverol et al., 2019) with the dataset identifier PXD020229.

EXPERIMENTAL MODEL AND SUBJECT DETAILS

Bacterial culture

Bacterial strains and insect cell lines used in this study are listed in the key resources table. Bacterial growth conditions can be found in method details.

Cell culture

Spodoptera frugiperda Sf9 cells were cultured in SF-900 SFM media (Gibco) at 27°C with shaking (160 rpm).

METHOD DETAILS

Plasmids

KKT4 fragments used in this study were amplified from *Trypanosoma brucei* genomic DNA and cloned into the pNIC28-Bsa4 expression vector using ligation-independent cloning (Gileadi et al., 2008) or cloned into the RSFDuet-1 vector (Novagen) using NEBuilder HiFi DNA Assembly Kit (New England Biolabs). All constructs were sequence verified. *Lm*KKT4$^{184–284}$ was cloned from *Leishmania mexicana* genomic DNA (kindly provided by Richard Wheeler), which contained an R218Q mutation. Due to a cloning error, which

failed to place a stop codon after the *Lm*KKT4$^{184–284}$ coding sequence, an additional 23 residues (EFELGAPAGRQACGRIMLKSNRK) from the vector were inserted at the C terminus. *Tc*KKT4$^{117–218}$ was cloned from a synthetic *Trypanosoma cruzi* KKT4 gene fragment, codon optimised for expression in *E. coli* (Llauro et al., 2018). Point mutants of the microtubule-binding domain were created using site-directed mutagenesis using PrimeSTAR Max DNA polymerase (Takara Bio).

pBA1371 (3FLAG-KKT4, KKT14, KKT15 in pACEBac2) was made as follows. First, KKT15 was amplified from genomic DNA with BA724/BA725 and cloned into pIDK using NcoI/NheI, making pBA336. KKT14 was amplified from genomic DNA with BA747/BA748 into pIDK (Geneva Biotech) using XhoI/NsiI, making pBA485. Then the KKT15 expression module from pBA336 cut with PI-SceI and BstXI was ligated into pBA485 cut with PI-SceI, making pBA515. Finally, pBA818 (FLAG-KKT4 in the pACEBac2 acceptor plasmid (Ishii and Akiyoshi, 2020) and pBA515 (KKT14, KKT15 in the pIDK donor plasmid) were fused using Cre recombinase with gentamycin-kanamycin selection, making pBA1371. pBA1469 (KKIP1 in pACEBac1) was made as follows. KKIP1 (codon optimised for expression in *Spodoptera frugiperda*) was subcloned from pBA1166 into pIDS (Geneva Biotech) using NheI/KpnI, making pBA1207. Then pBA1207 and pACEBac1 (Geneva Biotech) were fused using Cre recombinase with gentamycin-spectinomycin selection, making pBA1469. These plasmids (pBA1371 and pBA1469) were integrated into the DH10EmBacY baculoviral genome in DH10EmBacY *E. coli* cells to make bacmids (pBA1388 and pBA1540). Bacmids were purified from *E. coli* using a PureLink HiPure Plasmid Miniprep Kit (Thermo Fisher) and used to transfect Sf9 cells using Cellfectin II transfection reagent (Thermo Fisher), and baculovirus was amplified through three rounds of amplification (Llauro et al., 2018).

Protein expression and purification

Expression and purification of KKT4 fragments used for SEC-MALS, crystallographic studies and fluorescence anisotropy assays was done as follows. Transformed *E. coli* BL21(DE3) cells were inoculated into 5 ml of 2xTY medium containing 50 μg/ml kanamycin and grown overnight at 37°C. The next morning, 1 l of 2xTY medium with 50 μg/ml of kanamycin was inoculated with 5 ml of the overnight culture and grown at 37°C with shaking (200 rpm) until the OD_{600} reached ~0.6. Protein expression was induced with 0.2 mM IPTG for ~16 hr at 16°C. Cells were spun down at 3,400 g at 4°C and resuspended in lysis buffer (50 mM sodium phosphate, pH 7.5, 500 mM NaCl, and 10% glycerol) supplemented with protease inhibitors (20 μg/ml leupeptin, 20 μg/ml pepstatin, 20 μg/ml E-64 and 0.4 mM PMSF), benzonase nuclease (500 U/1 l culture), and 0.5 mM TCEP. All subsequent steps were performed at 4°C. Bacterial cultures were mechanically disrupted using a French press (1 passage at 20,000 psi) and the soluble fraction was separated by centrifugation at 48,000 g for 30 min. Supernatants were loaded on TALON beads (Takara Bio) pre-equilibrated with lysis buffer (1 ml of beads per 1 l of bacterial culture). Next, the beads were washed with lysis buffer without protease inhibitors and proteins were eluted with 50 mM sodium phosphate pH 7.5, 500 mM NaCl, 10% glycerol, 250 mM imidazole and 0.5 mM TCEP. To cleave off the His-tagged, samples were incubated with TEV protease in 1:50 w/w ratio overnight while being buffer-exchanged into 50 mM sodium phosphate, 500 mM NaCl, 10% glycerol, 5 mM imidazole, and 0.5 mM TCEP by dialysis. To increase the sample purity and remove the His-tag, samples were re-loaded on TALON beads pre-equilibrated with dialysis buffer and the flow-through was collected. Next, the samples were further purified using either two-step (ion exchange and size exclusion chromatography) or one-step (size exclusion chromatography) purification. To promote binding of proteins to the ion exchange column, samples were diluted with buffer A (25 mM HEPES pH 7.5 and 0.5 mM TCEP) to achieve the final NaCl concentration of 50 mM. Ion exchange chromatography was performed using either a 6 ml RESOURCE S or RESOURCE Q column (GE Healthcare) pre-equilibrated with 5% of buffer B (25 mM HEPES pH 7.5, 1 M NaCl and 0.5 mM TCEP). Proteins were eluted with a linear gradient from 5% to 100% of buffer B, concentrated using 3- or 10-kD MW Amicon concentrators (Millipore), and loaded on Superdex 75 or Superdex 200 16/60 (GE Healthcare) columns to further purify and buffer exchange into 25 mM HEPES pH 7.5, 150 mM NaCl with 0.5 mM TCEP. Fractions containing KKT4 were pooled, concentrated using a 3- or 10-kD MW Amicon concentrator (Millipore), and flash-frozen in liquid nitrogen for −80°C storage.

Expression of KKT4 mutants used in microtubule co-sedimentation assays was done as described above with the following modifications. After overnight expression at 16°C, cells were spun down at 3,400 g at 4°C and resuspended in lysis buffer (50 mM sodium phosphate, pH 7.5, 500 mM NaCl, and 10% glycerol) supplemented with protease inhibitors (20 μg/ml leupeptin, 20 μg/ml pepstatin, 20 μg/ml E-64 and 0.4 mM PMSF), benzonase nuclease (500 U/1 l culture), and 0.5 mM TCEP. All subsequent steps were performed at 4°C. Bacterial cultures were sonicated on ice (three rounds of 15 sec pulse and 1 min pause) and the soluble fraction was separated by centrifugation at 48,000 g for 30 min. Supernatants were incubated with TALON beads (Takara Bio) pre-equilibrated with lysis buffer (0.75 ml of beads per 1 l of bacterial culture). Next, the beads were washed 5 times with 10 ml of lysis buffer without protease inhibitors and proteins were eluted with 50 mM sodium phosphate pH 7.5, 500 mM NaCl, 10% glycerol, 250 mM imidazole and 0.5 mM TCEP. Samples were buffer exchanged using Zeba columns (Thermo Fisher) into BRB80 buffer (80 mM PIPES-KOH, pH 6.9, 1 mM EGTA, 1 mM $MgCl_2$) with 100 mM KCl and flash-frozen in liquid nitrogen for −80°C storage.

Expression and purification of isotopically labelled KKT4 fragments

Transformed *E. coli* BL21(DE3) cells were plated on agar plates containing 50 μg/ml kanamycin and incubated at 37°C overnight. After overnight incubation, a few colonies were inoculated into 5 ml of 2xTY medium containing 50 μg/ml kanamycin and grown at 37°C for 6 hr. Next, 50 ml of M9 minimal medium containing 50 μg/ml kanamycin supplemented with 1g/l $^{15}NH_4Cl$ and 4g/L [^{13}C]-*D*-glucose (CIL) as the sole nitrogen source was inoculated with 500 μl of bacterial culture. Cell growth was continued overnight at 37°C. Next, 5 ml of overnight culture was inoculated into 1l of M9 minimal medium supplemented with 1g/l $^{15}NH_4Cl$, 4g/L [^{13}C]-*D*-glucose and 50 μg/ml kanamycin. Cells were grown at 37°C to an OD_{600} of ~ 0.8. Protein expression was induced by 0.4 mM IPTG

and incubated overnight at 16°C with shaking (200 rpm). To purify isotopically labelled proteins, we followed the same protocol as for samples used in crystallography, which is described above.

Expression and purification of full length KKT4 from insect cells

To express full-length SNAP-6HIS-3FLAG-KKT4 (Llauro et al., 2018), 500 ml of Sf9 cell culture at 1–1.2 million cells/ml was infected with P3 baculovirus for ~72 hr before harvesting. Subsequent steps were performed at 4°C. Cells were pelleted at 700 *g* for 10 min, washed once with PBS, and resuspended in 10 ml BH0.25 (25 mM HEPES, pH 7.5, 0.2 % NP-40, 2 mM $MgCl_2$, 0.1 mM EDTA, 0.5 mM EGTA, 10% glycerol, and 250 mM NaCl) supplemented with 2× protease inhibitors (20 μg/ml leupeptin, 20 μg/ml pepstatin, 20 μg/ml E-64, and 0.4 mM PMSF) and benzonase nuclease (1000 U/1 l culture). Cells were lysed on ice using sonicator (three rounds of 15 sec pulse and 1 min pause) followed by centrifugation for 30 min at 45,000 *g*. The supernatant was incubated with 2 ml of anti-FLAG M2 affinity gel (Sigma) for 3 hr with constant rotation, followed by five washes with BH0.25 supplemented with 0.5 mM TCEP (10 ml each). Proteins were eluted from the beads with gentle agitation of beads in 2 ml BH0.25 containing 0.5 mg/ml 3FLAG peptide (Sigma) and 1× protease inhibitors. The sample was further purified using 1 ml HiTrap Heparin HP column preequilibrated with 5% of buffer B (buffer A: 25 mM HEPES, pH 7.5, with 0.5 mM TCEP; buffer B: 25 mM HEPES, pH 7.5, and 1 M NaCl with 0.5 mM TCEP) and eluted with a linear gradient from 5% to 100% of buffer B. Fractions containing SNAP-tagged KKT4 were pooled and concentrated by a 10-kD MW Amicon concentrator (Millipore). For crosslinking experiments with BS^3, FLAG-KKT4 was immunoprecipitated from insect cells transfected with baculovirus prepared from pBA1388 (3FLAG-KKT4, KKT14, KKT15), whereas crosslinking with EDC/Sulfo-NHS was performed on FLAG-KKT4 that was purified from insect cells transfected with baculoviruses prepared from pBA826 (3FLAG-KKT4) and pBA1540 (KKIP1), both purified according to the protocol described above.

Size Exclusion Chromatography with Multi-Angle Light Scattering (SEC-MALS)

MALS experiments were performed during size exclusion chromatography on analytical Superose 6 or Superdex 200 HR10/300 columns (GE Healthcare) equilibrated with 25 mM HEPES pH 7.5, 150 mM NaCl and 0.5 mM TCEP (for SNAP-6HIS-3FLAG-KKT4, 25 mM HEPES pH 7.5, 340 mM NaCl and 0.5 mM TCEP was used). Elution was monitored via online static light-scattering (DAWN HELEOS 8+, Wyatt Technology), differential refractive index (Optilab T-rEX, Wyatt Technology) and UV (SPD-20A, Shimadzu) detectors. Data were analysed using the ASTRA software package (Wyatt Technology).

Crystallization

All crystals were obtained in sitting drop vapour diffusion experiments in 96-well plates, using drops of 200 nl overall volume, mixing protein and mother liquor in a 1:1 ratio. Crystals of *Trypanosoma cruzi* (Sylvio X10) $KKT4^{117–218}$ (10.0 mg/ml) were grown at 18°C in Morpheus II HT-96 G3 solution (Molecular Dimensions) containing 0.1 M buffer system 4 (MOPSO, Bis-Tris) pH 6.5, 50% v/v precipitant mix 7 (20% w/v PEG 8000, 40% v/v 1,5-Pentanediol) and 100 mM amino acids II (0.2 M DL-Arginine hydrochloride, 0.2 M DL-Threonine, 0.2M DL-Histidine monohydratochloride monohydrate, 0.2 M DL-Hydroxylysine hydrochloride, 0.2 M trans-4-hydroxy-L-proline). Mother liquor served as a cryoprotectant. Crystals of *Leishmania mexicana* $KKT4^{184–284}$ (13.5 mg/ml) were grown at 4°C in ProPlex crystallisation screen (Molecular Dimensions) solution containing 0.1 M imidazole pH 7.0 and 50% v/v MPD. Mother liquor served as a cryoprotectant. Crystals of *Trypanosoma brucei* $KKT4^{463–645}$ (26.5 mg/ml) were grown at 4°C in Index crystallisation screen (Hampton Research) solution containing 0.1 M bis-Tris pH 5.5 and 2.0 M ammonium sulphate. Crystals were briefly transferred into mother liquor prepared with addition of 23% glycerol prior to flash-cooling by plunging into liquid nitrogen.

Diffraction data collection and structure determination

X-ray diffraction data from *Trypanosoma cruzi* (Sylvio X10) $KKT4^{117–218}$ and *Leishmania mexicana* $KKT4^{184–284}$ were collected at the I03 and I24 beamlines respectively, at the Diamond Light Source (Harwell, UK). The structures were solved using *ab initio* macromolecular phasing software, ARCIMBOLDO LITE optimised for coiled coils (Rodríguez et al., 2009, 2012) followed by initial model building with BUCCANEER (Cowtan, 2006). Further manual model building and refinement were completed iteratively using COOT (Emsley et al., 2010) and PHENIX (Liebschner et al., 2019). The data sets used for the final refinement were scaled to the high-resolution limit of 1.9 Å and processed using anisotropic scaling (Strong et al., 2006).

X-ray diffraction data from *Trypanosoma brucei* $KKT4^{463–645}$ were collected at the I24 beamline at the Diamond Light Source (Harwell, UK). The structure was solved using ARCIMBOLDO LITE (Rodríguez et al., 2009, 2012) followed by initial model building with BUCCANEER (Cowtan, 2006). The further model building and refinement were completed using COOT (Emsley et al., 2010) and PHENIX (Liebschner et al., 2019).

The final refinement statistics for three structures are summarised in Table 1. All structure figures were prepared using PyMOL (Delano, 2002). Protein coordinates have been deposited in the RCSB Protein Data Bank (http://www.rcsb.org/) with accession codes: PDB: 6ZPM (*Trypanosoma cruzi* (Sylvio X10) $KKT4^{117–218}$), PDB: 6ZPJ (*Leishmania mexicana* $KKT4^{184–284}$) and PDB: 6ZPK (*Trypanosoma brucei* $KKT4^{463–645}$).

NMR spectroscopy and analysis of NMR data

All NMR samples were prepared in 25 mM HEPES pH 7.2, 150 mM NaCl, 0.5 mM TCEP and 95% H_2O/5% D_2O. All NMR spectra were acquired using a 750 MHz spectrometer equipped with a Bruker Avance III HD console and a 5 mm TCI CryoProbe. All NMR data were processed using NMRPipe (Delaglio et al., 1995) and analysed using CCPN Analysis (Vranken et al., 2005).

^{1}H, ^{13}C and ^{15}N chemical shifts of $KKT4^{115–174}$, $KKT4^{145–232}$ and $KKT4^{115–343}$ were analysed using TALOS-N (Shen and Bax, 2013) and SSP (Marsh et al., 2006) to predict secondary structure propensities.

The $\{^{1}H\}$-^{15}N heteronuclear NOE was measured for 0.2–0.5 mM samples of $KKT4^{115–174}$, $KKT4^{145–232}$ and $KKT4^{115–343}$ using the TROSY-based heteronuclear NOE experiment recorded with and without ^{1}H saturation for 4.5 sec at 750 MHz (Zhu et al., 2000). The data sets were acquired using 128 complex t_1 increments, 96 scans per increment and with a ^{15}N sweep width of 1597.444 Hz for $KKT4^{115–174}$ and 1901.141 Hz for $KKT4^{145–232}$ and $KKT4^{115–343}$. 1K complex data points were recorded in the F_2 dimension with a sweep width of 9259.259 Hz. Data were collected at 20°C for $KKT4^{115–174}$ and $KKT4^{115–343}$ and at 30°C for $KKT4^{145–232}$. The $\{^{1}H\}$-^{15}N NOE was calculated as the ratio of the peak intensities in the spectra recorded with and without ^{1}H saturation. Peak heights were determined using CCPN Analysis (Vranken et al., 2005). Uncertainties in the $\{^{1}H\}$-^{15}N NOE values were estimated from 500 Monte Carlo simulations using the baseline noise as a measure of the error in the peak heights.

Partial alignment of the $KKT4^{115–174}$ fragment was achieved using C12E6/*n*-hexanol liquid crystals prepared as described by Rückert and Otting (Rückert and Otting, 2000). A 10% C12E6/*n*-hexanol solution was prepared in HEPES buffer and added to the protein sample to achieve the desired final concentration of 5% for $KKT4^{115–174}$.

^{15}N-^{1}HN RDCs were measured at 20°C for using BEST TROSY and BEST semi-TROSY experiments (Schulte-Herbruggen and Sorensen, 2000; Lescop et al., 2007). 128 complex points and a sweep width of 1597.444 Hz was collected in F_1 (^{15}N). Residual dipolar couplings were measured as the difference between the splitting observed in the isotropic and aligned data sets. Error bars were derived from three measurements of the RDCs.

The principle components (A_{xx}, A_{yy} and A_{zz}) and orientation (φ, θ and ψ) of the molecular alignment tensor were fitted to minimise the $\chi 2$ between the experimental and calculated RDCs using the *T. cruzi* X-ray coordinates. The sequence of $KKT4^{115–174}$ was aligned with different positions in the *T. cruzi* X-ray structure in order to find the best fit of the *T. brucei* residues into the *T. cruzi* heptad repeats. Residues with $\{^{1}H\}$-^{15}N heteronuclear NOE values of less than 0.6 were excluded from the fitting procedure. Q values were calculated to assess the quality of the fits between experimental and calculated RDCs using the method of (Cornilescu et al., 1998).

Modelling of *T. brucei* $KKT4^{115–232}$ and $KKT4^{115–645}$

Homology models for the two coiled-coil regions of *T. brucei* $KKT4^{115–232}$ were generated using Modeller 9 v24 (Webb and Sali, 2016), the X-ray structure of the *T. cruzi* $KKT4^{117–218}$ coiled coil, and the sequence alignments of *T. brucei* and *T. cruzi* derived from the RDC data collected for $KKT4^{115–174}$. Modeller was run using the fully automated comparative modelling mode. Random extended structures for the N and C termini of $KKT4^{115–232}$ and for the inter-helix linker were generated using X-PLOR (Brünger, 1992); these represent possible conformations that these residues might sample in solution. These coordinates were merged with the two coiled-coil dimer models to generate an overall model for *T. brucei* $KKT4^{115–232}$. The model for $KKT4^{115–645}$ was created by merging the model for $KKT4^{115–232}$, the X-ray structure of the BRCT domain ($KKT4^{463–645}$), and two random structures for disordered residues 233–473 generated using X-PLOR (Brünger, 1992). The two random structures selected to represent 233–473 were chosen from a group of ten structures to illustrate possible conformations in which the BRCT domain is in close proximity to the N-terminal coiled coil and is more distant from this region.

Microtubule co-sedimentation assay

Taxol-stabilised microtubules were prepared by mixing 2.5 μl of 100 μM porcine tubulin (Cytoskeleton) resuspended in BRB80 (80 mM Pipes-KOH pH 6.9, 1 mM EGTA, and 1 mM $MgCl_2$) with 1 mM GTP, 1.25 μl of BRB80, 0.5 μl of 40 mM $MgCl_2$, 0.5 μl of 10 mM GTP, and 0.25 μl DMSO, and incubated for 20 min at 37°C. Then, 120 μl of pre-warmed BRB80 containing 12.5 μM Taxol (paclitaxel; Sigma) was added to the sample to achieve 2 μM microtubule solution. To achieve higher concentrations of microtubules, the protocol described above was scaled up. Prior to the assay, KKT4 fragments were buffer-exchanged into BRB80 with 100 mM KCl using Zeba desalting spin columns (Thermo Fisher). For the microtubule co-sedimentation assay, 20 μl of KKT4 fragments (4 μM) were mixed with 20 μl of microtubules (2 μM) and incubated for 45 min at room temperature. For a no-microtubule control, KKT4 fragments were mixed with BRB80 with 12.5 μM Taxol. The samples were spun at 20,000 g at room temperature for 10 min, and the supernatant was collected. To the tube with a pellet, we added 40 μl of chilled BRB80 with 5 mM $CaCl_2$ and incubated on ice for 5 min to depolymerise microtubules. Following the incubation, samples were boiled for 5 min before analysis by SDS-PAGE gels stained with SimplyBlue Safe Stain (Invitrogen).

Fluorescence anisotropy assay

The DNA-binding analysis of KKT4 was performed in binding buffer (25 mM HEPES pH 7.5, 50 mM NaCl and 0.5 mM TCEP) using a 50-bp DNA probe BA3098 (~36% GC content), which is part of the *Trypanosoma brucei* centromere CIR147 sequence (Obado et al., 2007), labelled at the 5' end with 6-carboxyfluorescein (6-FAM). Prior to the assay, KKT4 proteins were buffer-exchanged into the binding buffer using Zeba spin desalting columns (Thermo Fisher). $KKT4^{2–645}$ (0.67 μM) and $KKT4^{463–645}$ (1 μM) samples were mixed with DNA probe in the binding buffer to a final DNA concentration of 1 nM. Next, the proteins were serially diluted in the binding buffer containing 1 nM DNA in the 2:3 v/v ratio. The binding reactions were incubated for 30 min at room temperature and fluorescence anisotropy was measured using a PHERAstar FS next-generation microplate reader (BMG LABTECH). Equilibrium dissociation constants (K_D) were calculated by fitting the data in SigmaPlot (Monks, 2002). The phosphopeptide-binding experiments were carried out using fluorescently-labelled phosphorylated (DICGISGQ(pS)VRYSLND) and unphosphorylated peptide probes

(DICGISGQSVRYSLND). KKT4$^{463-645}$ (170 μM) was mixed with the probe (100 nM) and serially diluted in a 2:3 v/v ratio. Incubation of the samples and measurements were done as described above.

Chemical crosslinking mass spectrometry (XL-MS)

Prior to the experiment, bis(sulfosuccinimidyl)suberate, BS^3 (Thermo Fisher), crosslinker was equilibrated at room temperature for 2 hr and then resuspended to 0.87 mM in distilled water. Immediately after, 2 μl of the crosslinker was mixed with 18 μl of ~5 μM KKT4 in 25 mM HEPES pH 8.0, 2 mM $MgCl_2$, 0.1 mM EDTA, 0.5 mM EGTA-KOH, 10% glycerol, 250 mM NaCl, and 0.1% NP40. The crosslinking reaction was incubated on ice for 60 min. 1-ethyl-3-(3-dimethylaminopropyl)carbodiimide hydrochloride (EDC) and *N*-Hydroxysulfosuccinimide sodium salt (Sulfo-NHS) were resuspended in distilled water to 4 mM and 10 mM respectively. Immediately after, 1 μl of EDC and 1 μl of Sulfo-NHS were mixed with 18 μl of ~5 μM KKT4. The crosslinking reaction was incubated at room temperature for 60 min. Following the incubation, all the crosslinking samples were boiled for 10 min and resolved on a NuPAGE 4–12% gradient polyacrylamide gel (Invitrogen). Gels were stained using SimplyBlue (Invitrogen) and bands corresponding to crosslinked KKT4 were cut out and subjected to mass spectrometry. Gel bands were destained by cycles of incubation with 50% acetonitrile in 100 mM triethylammonium bicarbonate (TEAB) and 100% acetonitrile. Subsequently, dried gel bands were incubated with 10 mM TCEP in 100 mM TEAB for 30 min at room temperature followed by centrifugation. Supernatant was removed. Subsequently, 50 mM 2-chloroacetamide in 100 mM TEAB was added to the gel and incubated for 30 min at room temperature in the dark. Gel bands were subsequently washed with 100% acetonitrile 3 times followed by addition of 100 ng of trypsin and digested overnight at 37°C. Subsequently, the supernatant was extracted. The gel pieces were then washed with an 5% formic acid and then acetonitrile. Both washes were combined with the initial supernatant and the mixture was then dried down.

Peptides were resuspended in 5% formic acid and 5% DMSO and analysed by LC-MS with an Ultimate 3000 UHPLC system (Thermo Fischer Scientific) coupled to an QExactive mass spectrometer (Thermo Fischer Scientific) through an EASY-Spray nano-electrospray ion source (Thermo Fischer Scientific). The peptides were trapped on a C18 PepMap 100 pre-column (300 μm i.d. x 5 mm, 100Å, Thermo Fisher Scientific) using solvent A (0.1% formic acid in water). The peptides were separated on an in-house packed analytical column (75 μm i.d. x 50 cm packed with ReproSil-Pur 120 C18-AQ, 1.9 μm, 120 Å, Dr. Maisch GmbH) using a linear gradient (length: 45 min, 15% to 50% solvent B (acetonitrile with 0.1% formic acid)). Acquisition was performed in data-dependent mode (DDA). Full scan MS spectra were acquired in the Orbitrap (scan range 350–1,500 m/z, resolution 70,000, AGC target 3 x 106, maximum injection time 50 ms) followed by 10 MS/MS events at 30% NCE (resolution 17,500, AGC target 5 x 104, maximum injection time 120 ms, isolation window 1.5 m/z) with first fixed mass at 180 m/z. Charge exclusion was selected for unassigned 1+ and 2+ ions.

MS data were converted into mgf format using pParse and searched by the pLink software (Chen et al., 2019) (version 1 for BS^3 dataset and version 2 for EDC dataset) using FASTA databases containing KKT1–4, 6, 7–11, 14, 15, 20, and α/β tubulins without KKIP1 (for BS3 dataset) or with KKIP1 (for EDC dataset). Search parameters were as follows: maximum number of missed cleavages = 2, fixed modification = carbamidomethyl-Cys, variable modification 1 = Oxidation-Met, variable modification 2 = Glu to pyro-Glu. Crosslinks that have score < 1 x 10^{-7} were visualised using xiNET (Combe et al., 2015) (Tables S4 and S5).

Interaction of the microtubule-binding and BRCT domains

^{1}H-^{15}N BEST TROSY spectra were collected at 20°C for 0.2 mM KKT4$^{115–174}$, KKT4$^{115–232}$ and 30°C for 0.2 mM KKT4$^{145–232}$ alone and in the presence of 0.22 mM KKT4^{BRCT}. Following addition of KKT4^{BRCT} the samples were incubated at room temperature for ~30 min. Interaction was monitored by comparing peak positions; the combined chemical shift changes of ^{1}HN and ^{15}N are reported in Hz in Figures S8A and S8B.

Multiple sequence alignment

Protein sequences and accession numbers for KKT4 and KKT8 homologues used this study were retrieved from the TriTryp database (Aslett et al., 2010), UniProt (UniProt, 2019), or a published study (Butenko et al., 2020). Searches for homologous proteins were done using BLAST in the TriTryp database (Aslett et al., 2010) or Jackhmmer on the UniProtKB proteome database using a default setting (HMMER web version 2.24) (Potter et al., 2018). Multiple sequence alignment was performed with MAFFT (L-INS-i method, version 7) (Katoh et al., 2019) and visualised with the Clustalx colouring scheme in Jalview (version 2.10) (Waterhouse et al., 2009).

QUANTIFICATION AND STATISTICAL ANALYSIS

Intensities of gel bands in the microtubule co-sedimentation assays were calculated using ImageJ (Schneider et al., 2012). All measured P intensities were subtracted by the intensity of the P fraction at 0 μM microtubules. Fraction bound was calculated using the following equation: [P]/([S]+[P]). S and P stand for supernatant and pellet respectively. Standard deviation was used to generate error bars for all graphs in this study by evaluating data from at least N=3 experiments.

Structure, Volume *29*

Supplemental Information

Structural characterization of KKT4, an unconventional microtubule-binding kinetochore protein

Patryk Ludzia, Edward D. Lowe, Gabriele Marcianò, Shabaz Mohammed, Christina Redfield, and Bungo Akiyoshi

Figure S1

A

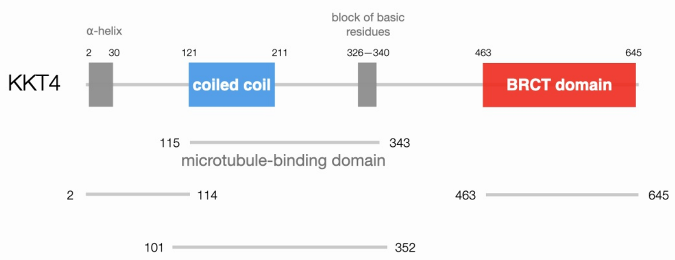

B

Protein	Concentration [μM]	Monomer molecular weight [kDa]	Observed molecular weight [kDa]	Estimated oligomerisation state
SNAP-6HIS-3FLAG-KKT4$^{2-645}$	20/17.5/10/6/4	92	334/275/240/195/170	Tetramer-dimer mixture
KKT4$^{463-645}$	100	20	19	Monomer
KKT4$^{101-352}$	35	28	55	Dimer
KKT4$^{2-114}$	1000/800/500/350/170	11	37.9/34.6/31.5/32.8/28.1	Tetramer–trimer or tetramer-dimer mixture

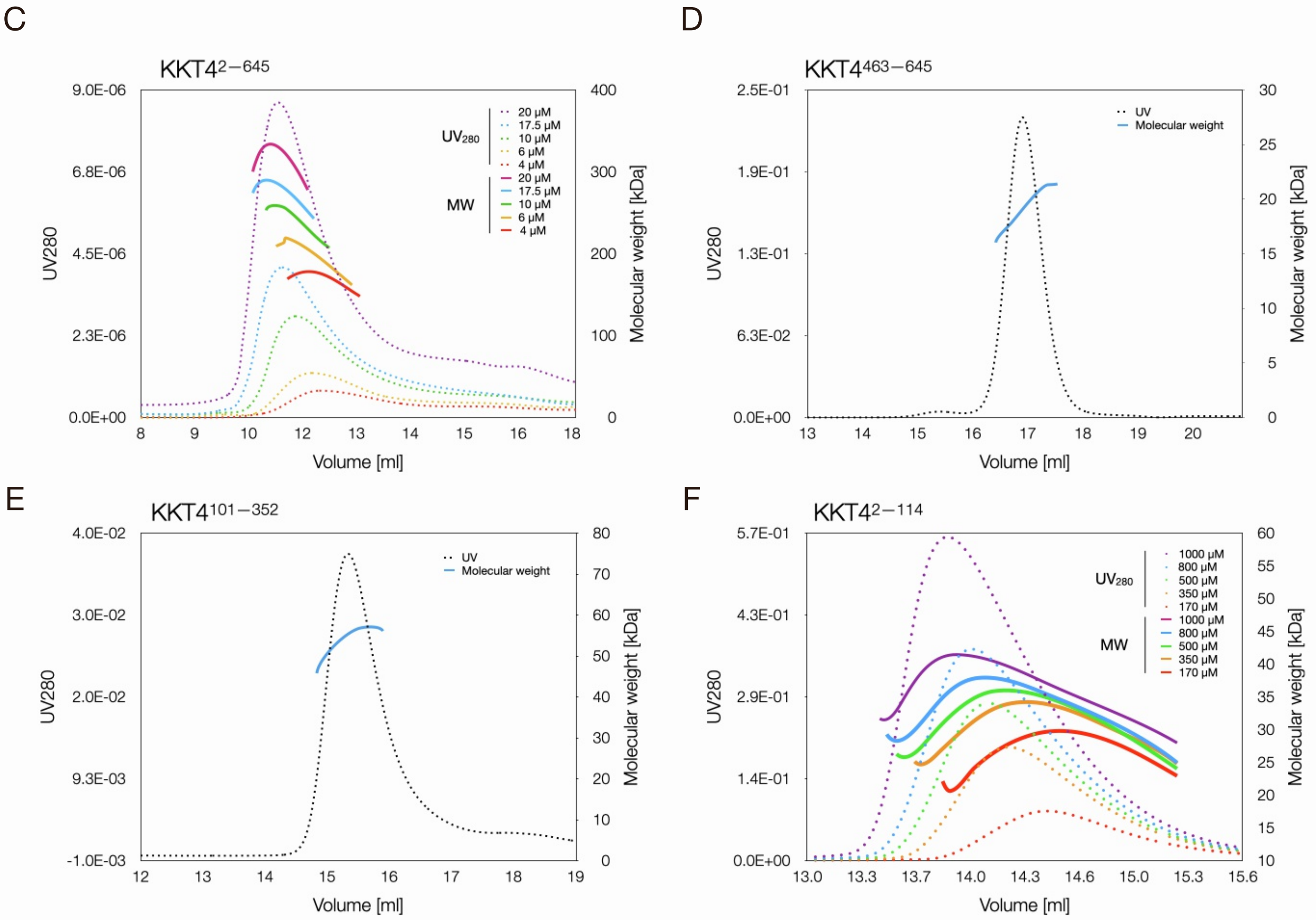

Figure S1. SEC-MALS analysis of KKT4 fragments, Related to Figure 1

A. Cartoon representation of KKT4 fragments used in SEC-MALS experiments

B. Summary table showing analysis of SEC-MALS results. Superose 6 HR10/300 was used for $KKT4^{2–645}$ and Superdex 200 HR10/300 was used for $KKT4^{463–645}$, $KKT4^{101–352}$ and $KKT4^{2–114}$. Reported sample concentration is the concentration before injecting the protein into the column. The actual concentration of the protein during the MALS experiment is ~10 fold lower due to the dilution factor during the SEC run.

C. SEC-MALS elution profile of SNAP-3FLAG-6HIS-$KKT4^{2–645}$ at different concentrations. The UV signal is plotted against the elution volume (dotted lines). Molecular weight is indicated as a thick line for each peak. The molecular weight at the highest concentration is estimated to be around 334 kDa, indicating a tetramer. The equilibrium shifts towards lower molecular weights, reaching 170 kDa (dimer) at the lowest concentration tested.

D. SEC-MALS elution profile of $KKT4^{463–645}$. The molecular weight is estimated to be around 19 kDa, indicating a monomer.

E. SEC-MALS elution profile of $KKT4^{101–352}$. The molecular weight is estimated to be around 55 kDa, indicating a dimer.

F. SEC-MALS elution profile of $KKT4^{2–114}$ samples at different concentrations. The lower the protein concentration, the larger the elution volume of the protein. This suggests that the oligomerisation state of $KKT4^{2–114}$ is concentration dependent, ranging from tetramer (high concentration) to trimer (low concentration).

Figure S2

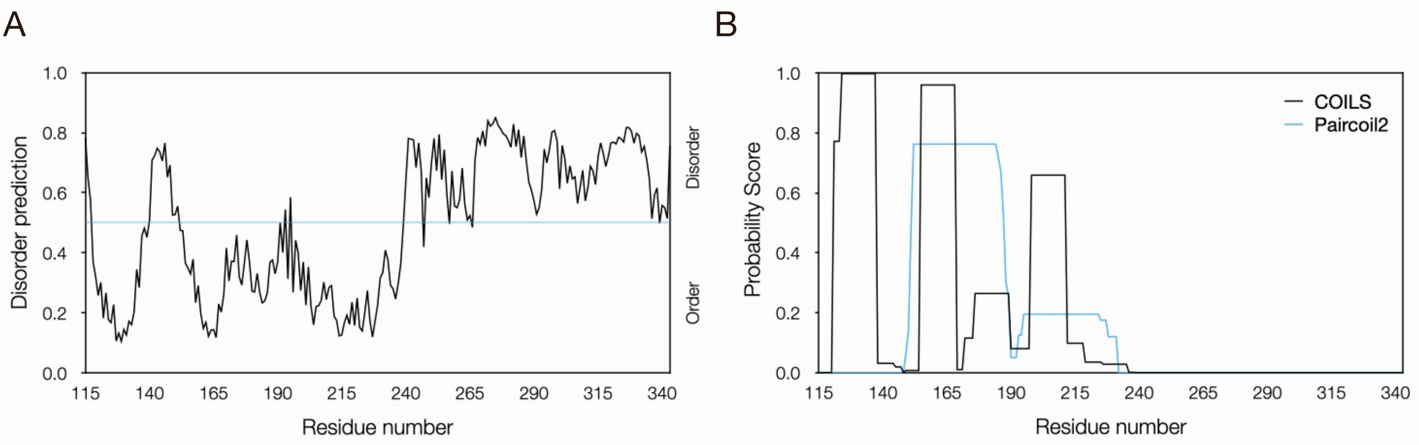

Figure S2. Structure predictions for *T. brucei* KKT4, Related to Figure 1

A. Disorder prediction for KKT4$^{115–343}$ using DisEMBL (Linding et al., 2003). The N-terminus of the microtubule-binding domain is predicted to be mainly ordered (118–239), while the C-terminus is predicted to be mostly disordered. The blue horizontal line at 0.5 indicates the boundary between predicted order (<0.5) and disorder (>0.5).

B. Coiled coil prediction for KKT4$^{115–343}$ using COILS (window of 14 residues) (Lupas et al., 1991) and Paircoil2 (window of 21/28 residues) (McDonnell et al., 2006). COILS predicts coiled coils from 121 to 211 with breaks between 138–154 and 169–189, while Paircoil2 does not predict coiled coil at the N-terminus of the domain between residues 121–137.

Figure S3

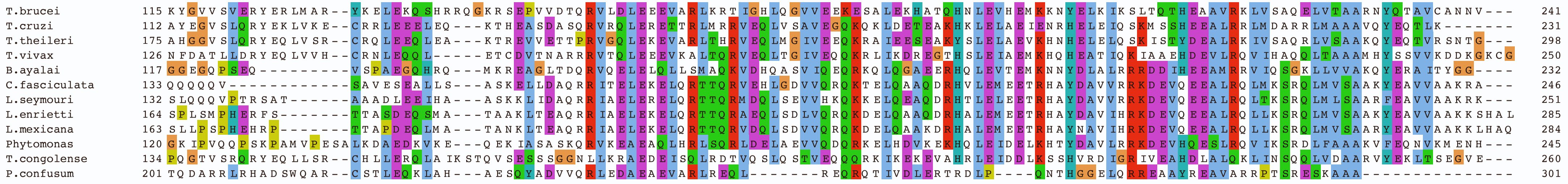

Figure S3. Multiple sequence alignment of the N-terminal part of KKT4 microtubule-binding domain, Related to Figure 2

KKT4 protein sequences from several kinetoplastid species were aligned using MAFFT (Katoh et al., 2019) and visualised with the CLUSTALX colouring scheme in Jalview (Waterhouse et al., 2009).

Figure S4

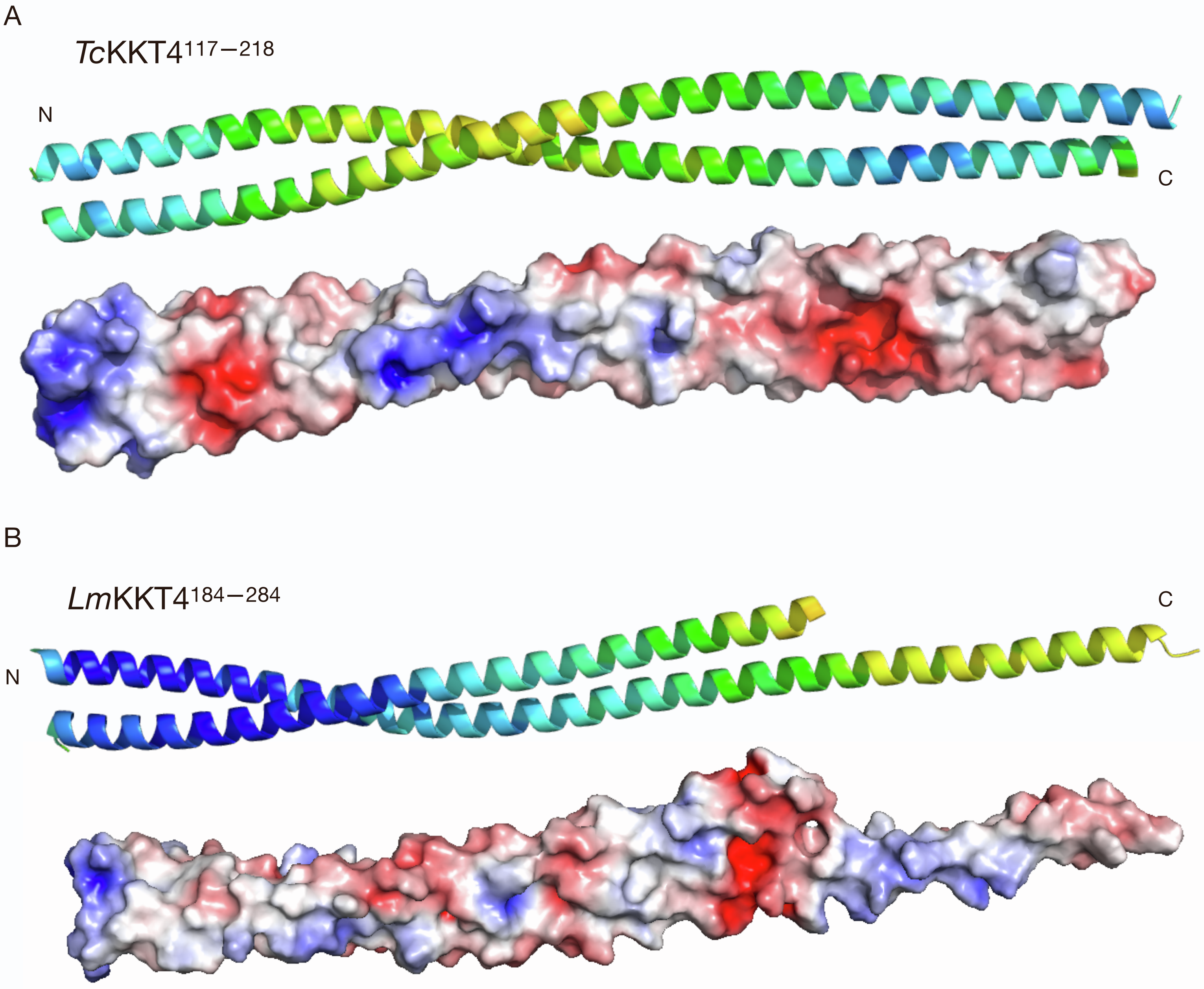

Figure S4. Ribbon models colour-coded by B-factors and the electrostatic surface potentials of *Tc*KKT4$^{117–218}$ and *Lm*KKT4$^{184–284}$, Related to Figure 2

B-factors for Cα atoms have been represented for *Tc*KKT4$^{117-218}$ (A) and *Lm*KKT4$^{184-284}$ (B) using a blue to red spectrum indicating low to high values, respectively. The figures were rendered using PyMol (DeLano, 2002). Below the ribbon models, a surface electrostatic potential is shown. Red to blue, -5 kbT to +5 kbT, as calculated by APBS electrostatic plugin in Pymol (Jurrus et al., 2018).

Figure S5

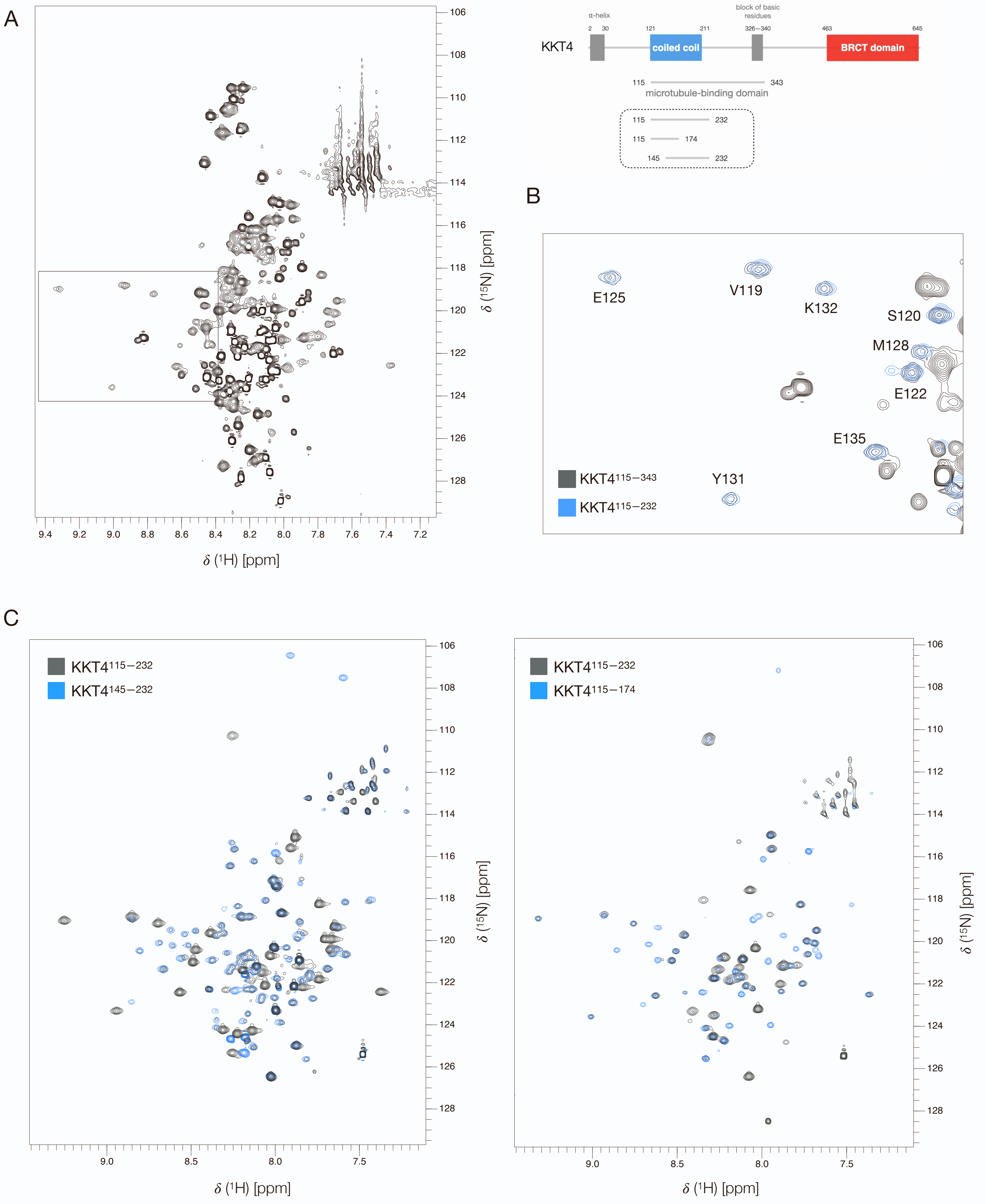

A
δ (¹H) [ppm]
δ (¹⁵N) [ppm]
KKT4
α-helix
coiled coil
block of basic residues
BRCT domain
2 30
121 211
326–340
463 645
115 343
microtubule-binding domain
115 232
115 174
145 232
B
E125
V119
K132
S120
M128
E122
E135
Y131
KKT4115–343
KKT4115–232
C
KKT4115–232
KKT4145–232
KKT4115–232
KKT4115–174

Figure S5. ^{1}H-^{15}N BEST-TROSY spectra of KKT4 fragments. , Related to Figure 3

A. 750 MHz ^{1}H-^{15}N BEST-TROSY spectrum of KKT4$^{115–343}$. The spectrum is contoured so that both weak and strong peaks are visible. Peaks in the region of 111–114 ppm and upfield of ~7.6 ppm are artefacts in the BEST-TROSY arising from incomplete cancellation of signals from the side chain amides of asparagine and glutamine.

B. Overlay of a small region of the ^{1}H-^{15}N BEST-TROSY spectra of KKT4$^{115–343}$ (indicated with a box in (A) and KKT4$^{115–232}$. N-terminal peaks overlay well between the two constructs, suggesting that the structure of the KKT4 N-terminus is similar in both fragments.

C. Overlay of 750 MHz ^{1}H-^{15}N BEST-TROSY spectra of KKT4$^{115–232}$ and KKT4$^{115–174}$ (right panel) or KKT4$^{115–232}$ and KKT4$^{145–232}$ (left panel). Most of the peaks in the spectra of KKT4$^{115–174}$ and KKT4$^{145–232}$ overlay with peaks observed for KKT4$^{115–232}$, indicating that the dissection approach used was justified. Peaks that do not overlay arise from the different C-terminal sequence in KKT4$^{115–174}$ and N-terminal sequence in KKT4$^{145–232}$. Some peaks visible in the spectra of the shorter KKT4 fragments are not visible in the longer construct, KKT4$^{115–232}$.

Figure S6

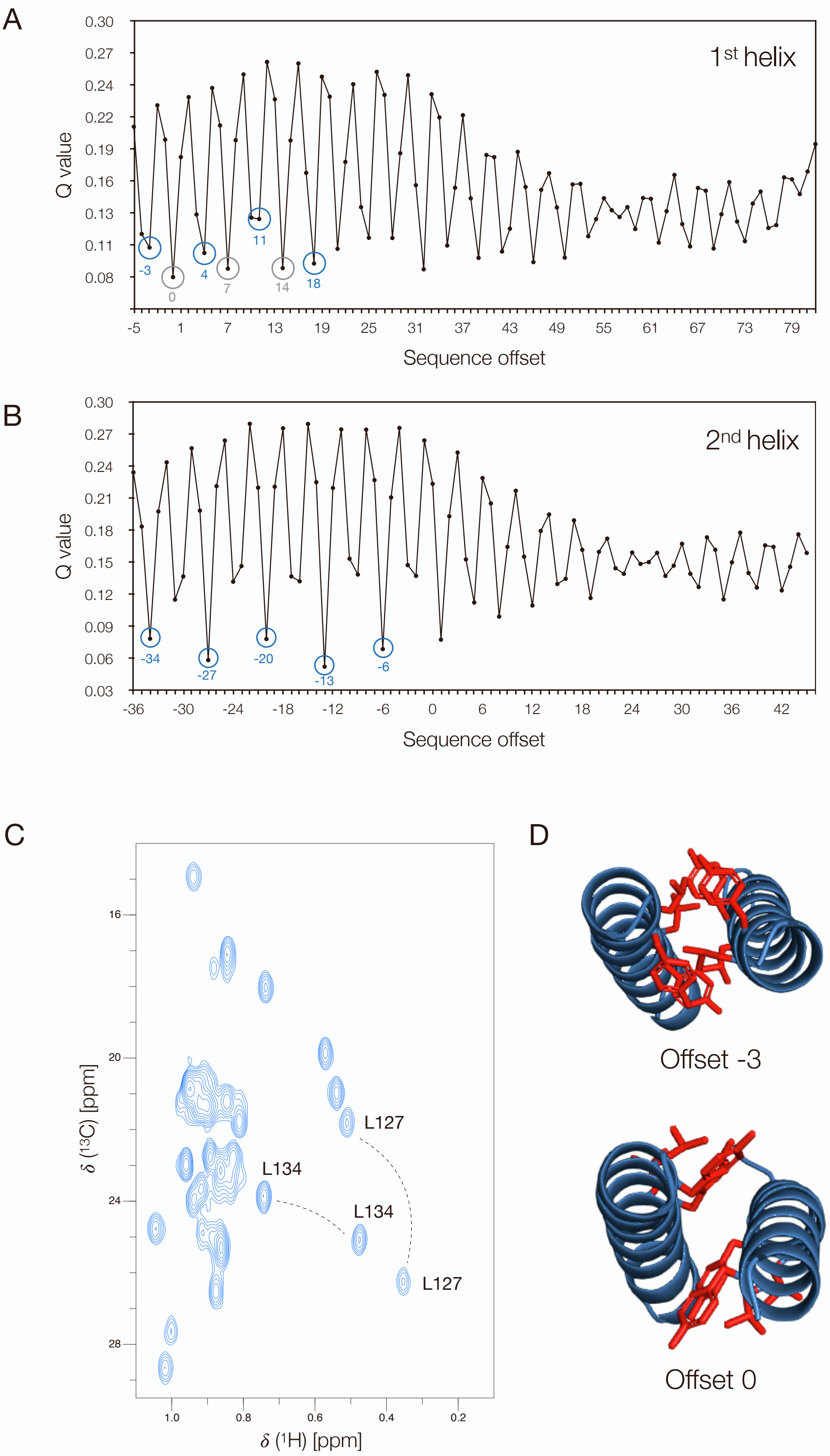

A
1st helix
Q value
Sequence offset
-3
0
4
7
11
14
18
B
2nd helix
Q value
Sequence offset
-34
-27
-20
-13
-6
C
L127
L134
L134
L127
δ (13C) [ppm]
δ (1H) [ppm]
D
Offset -3
Offset 0

Figure S6. Fitting the experimental RDC data using the *Tc*KKT4$^{117–218}$ crystal structure, Related to Figure 3

The experimental RDCs for residues 126–135 from the 1st helix (A) and 154–170 from the 2nd helix (B) from *T. brucei* KKT4$^{115–174}$ were fitted to the calculated RDCs based on the *T. cruzi* KKT4 X-ray structure. The relative alignment of the *T. brucei* and *T. cruzi* sequences was varied and the quality of the fit (Q value) of the experimental to predicted RDCs was measured. The Q value obtained is plotted as a function of the sequence offset (the number that is added to the *T. brucei* sequence before comparison with RDCs calculated for the *T. cruzi* sequence). For an ideal coiled-coil structure, the Q value should vary over a heptad repeat but the fits should be identical from one heptad repeat to the next. In contrast, for a more variable structure, where the interaction of the two helices varies along the length of the coiled coil, the quality of the fit may change along the sequence. For both helices, several good fits were found when the residues from *T. brucei* were aligned with the first half of the *T. cruzi* sequence, corresponding to the regular coiled-coil structure found between residues 121 and 176. Poorer agreement was obtained when the *T. brucei* helices were aligned with the C-terminal half of the *T. cruzi* structure, which shows less supercoiling. For the 2nd helix (residues 154–170, shown in (B) low Q values are found, at intervals of 7 residues (indicated by blue circles), when the residues from *T. brucei* were aligned with the residues in the N-terminal half of the *T. cruzi* sequence. The sequence alignment of these two proteins (Figure 2A) in the region of the 2nd helix suggests an offset of -6 residues, which is consistent with the fits of the RDC data, but offsets of -13, -20, -27 and -34 also produce good fits (indicated by blue circles). Predictions using the COILS server suggest that the hydrophobic residues identified as occupying the *a*/*d* positions in the *T. brucei* heptad align with the *a/d* residues in the *T. cruzi* structure for offsets of -6, -13, etc. For the 1st helix (residues 126–135, shown in (A)) low Q values are found for two sets of residues, at intervals of 7 residues. The lowest Q values are for sequence offsets of 0, 7, 14 (indicated by grey circles) but low Q values are also found for offsets of -3, 4, 11, 18 (indicated by blue circles); this type of degeneracy has been observed previously for the coiled-coil domain of cGK1$\alpha^{9\text{-}44}$ (Schnell et al., 2005). (C) The ^{1}H-^{13}C HSQC of KKT4$^{115\text{-}174}$ shows that both of the methyl groups of L127 and one of the methyl groups from L134 have ^{1}H peaks that are shifted upfield (to the right) of the peaks arising from most other L/V/I residues (around 0.7–1.05 ppm). These upfield shifts are due to the interaction of L127 and L134 with the aromatic rings of Y124 and Y131 and can be used to resolve the degeneracy between the two possible sequence offsets. Homology models for the 1st helix of *T. brucei* KKT4$^{115–174}$ have been built with Modeller using the *T. cruzi* X-ray structure and sequence alignments based on sequence offsets of -3 and 0 residues; these are shown in (D) with the side chains of Y124/L127/Y131/L134 shown as red sticks. The -3 sequence offset places Y124/Y131 and L127/L134 in positions *a* and *d* of the heptad, respectively, where they interact closely. The 0 sequence offset places these pairs of residues in positions *d* and *g*; L127/L134 in the *g* position are not closely packed at the inter-helix interface. ^{1}H chemical shifts for the L127 and L134 methyl groups were then predicted for the two alternative coiled-coil models. Only the homology model generated with a sequence offset of -3 residues predicts upfield shifts for the methyl groups of L127 and L134 due to their close proximity to Y124 and Y131 within the same helix and between helices in the packing interface. The sequence alignment of the *T. brucei* and *T. cruzi* proteins (Figure 2A) in the region of the 1st helix suggests an offset of -3 residues, which is consistent with the RDC data and chemical shift analysis of the homology models.

Figure S7

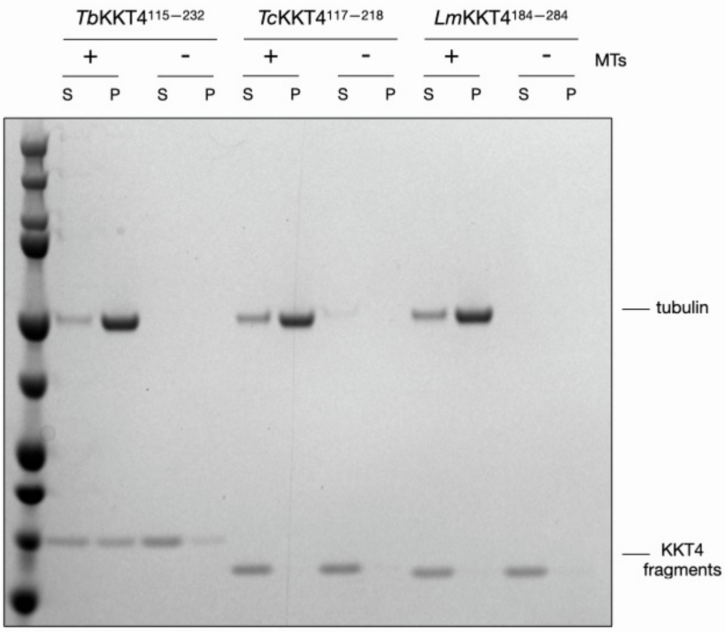

Figure S7. Microtubule-binding activity of KKT4 coiled coils, Related to Figure 5

Microtubule co-sedimentation assay of *Tb*KKT4$^{115-232}$, *Tc*KKT4$^{117-218}$ and *Lm*KKT4$^{184-284}$. Unlike *T. brucei* KKT4, coiled-coil regions from *T. cruzi* and *L. mexicana* did not co-sediment with taxol-stabilised microtubules at the concentration tested. S and P correspond to supernatant and pellet fractions, respectively.

Figure S8

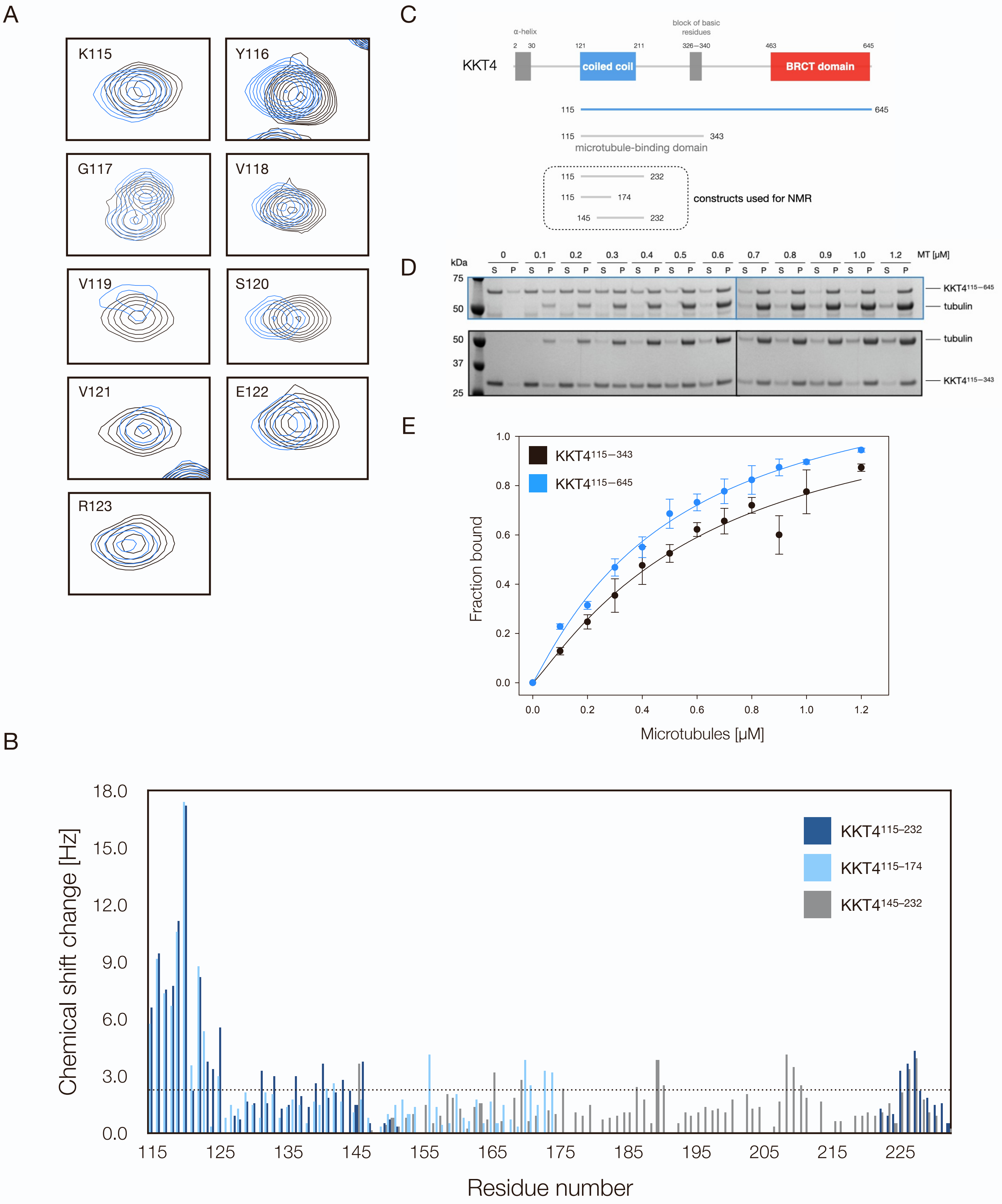

A
K115
Y116
G117
V118
V119
S120
V121
E122
R123
B
Chemical shift change [Hz]
18.0
15.0
12.0
9.0
6.0
3.0
0.0
115
125
135
145
155
165
175
185
195
205
215
225
Residue number
KKT4115–232
KKT4115–174
KKT4145–232
C
α-helix
block of basic residues
2
30
121
211
326−340
463
645
KKT4
coiled coil
BRCT domain
115
645
115
343
microtubule-binding domain
115
232
115
174
145
232
constructs used for NMR
D
kDa
0
0.1
0.2
0.3
0.4
0.5
0.6
0.7
0.8
0.9
1.0
1.2
MT [μM]
S
P
75
50
37
25
KKT4115−645
tubulin
tubulin
KKT4115−343
E
KKT4115−343
KKT4115−645
Fraction bound
1.0
0.8
0.6
0.4
0.2
0.0
0.0
0.2
0.4
0.6
0.8
1.0
1.2
Microtubules [μM]

Figure S8. N-terminus of microtubule-binding domain directly interacts with BRCT domain, Related to Figure 7

A. Magnification of peaks that showed chemical shift changes upon addition of the KKT4 BRCT domain to the sample. Peaks coming from ^{15}N-KKT4$^{115–232}$ and ^{15}N-KKT4$^{115–232}$/KKT4^{BRCT} spectra are coloured in black and blue respectively.

B. Chemical shift changes observed upon addition of KKT4^{BRCT} to KKT4$^{115–232}$ (dark blue), KKT4$^{115–174}$ (light blue), or KKT4$^{145–232}$ (grey) have been plotted against the KKT4 sequence. The dotted line represents the average value (2.2 Hz) of all chemical shift changes observed.

C. Cartoon representation showing KKT4 fragments used for NMR to study the interaction between the BRCT domain and the coiled coil-region. KKT4$^{115–645}$, analysed in panel D and E is shown as a blue line.

D. Microtubule co-sedimentation assay of KKT4$^{115−645}$ and KKT4$^{115−343}$, showing that the presence of the BRCT domain does not significantly affect the affinity to microtubules. S and P correspond to supernatant and pellet fractions, respectively.

E. Plot showing the fraction bound against the concentration of microtubules between KKT4$^{115–645}$ and KKT4$^{115–343}$. Error bars are standard deviations from three independent measurements.
